# Cytoskeletal protein KRT14 governs cisplatin resistance by modulating eIF4H-dependent ACOX2 translation and lipid metabolism in bladder cancer

**DOI:** 10.1038/s41419-025-08369-3

**Published:** 2025-12-24

**Authors:** Shenghua Liu, Tingting Liu, Chen Yang, Zezhong Mou, Lujia Zou, Haowen Jiang

**Affiliations:** Department of Urology, Huashan hospital, Fudan University, Shanghai, China

**Keywords:** Cancer, Cell biology

## Abstract

Cisplatin-based chemotherapy remains a mainstay for the treatment of bladder cancer (BLCA); however, its clinical efficacy is frequently compromised by the emergence of chemoresistance, which leads to poor patient outcomes. Although known mechanisms—such as alterations in drug efflux, DNA repair, and key signaling pathways—have been implicated, they fail to fully explain the clinical complexity of cisplatin resistance, indicating that additional molecular drivers remain undiscovered. Keratin 14 (KRT14), an intermediate filament protein associated with aggressive BLCA subtypes, is consistently upregulated in cisplatin-resistant tumors, yet its precise mechanistic role in resistance remains unclear. In this study, we elucidate the functional contribution of KRT14 to cisplatin resistance in BLCA using patient-derived tissues, established cell lines, xenograft mouse models, and a suite of molecular interaction assays. Our results demonstrate that KRT14 is significantly upregulated in BLCA tissues, correlates with poor clinical prognosis, and functionally drives cisplatin resistance both in vitro and in vivo. Mechanistically, we identify a novel and direct interaction between KRT14 and the translation initiation factor eIF4H, specifically through the N-terminal Head domain of KRT14. This interaction modulates the association of eIF4H with the core eIF4F complex, thereby selectively promoting the translation of Acyl-CoA Oxidase 2 (ACOX2) mRNA through its 5’ untranslated region. We further show that ACOX2 is essential for mediating the effects of KRT14 on lipid metabolism, cell proliferation, survival, and ultimately, cisplatin resistance. Collectively, our findings reveal that KRT14 contributes to chemoresistance in BLCA not only via its structural roles but also by directly regulating translational machinery through eIF4H, leading to upregulation of the metabolic enzyme ACOX2. The newly defined KRT14–eIF4H–ACOX2 axis orchestrates lipid metabolic reprogramming and cell survival, underscoring the KRT14–eIF4H interface as a promising therapeutic target for overcoming cisplatin resistance in BLCA.

## Introduction

Bladder cancer (BLCA) constitutes a major global health burden, distinguished by its high recurrence rate and significant morbidity and mortality, particularly in the muscle-invasive form (MIBC) [1, 2]. Although cisplatin-based combination chemotherapy remains the standard of care for MIBC and advanced BLCA and provides substantial initial benefit for many patients, the development of chemoresistance—whether intrinsic or acquired—represents a formidable clinical challenge [3, 4]. Resistance inevitably leads to treatment failure, tumor progression, and poor patient outcomes, underscoring the urgent need to unravel the molecular mechanisms driving this phenomenon [5].

Extensive research has revealed multiple mechanisms contributing to cisplatin resistance in BLCA, including altered drug transport [6], increased DNA repair capacity [7], evasion of apoptosis [8], and dysregulation of key signaling pathways [9, 10]. However, these established mechanisms fail to fully explain the clinical complexity of chemoresistance observed in BLCA patients [11]. Among emerging candidates, cytoskeletal proteins have gained attention as regulators that extend beyond mere structural support, influencing signaling networks and cellular stress responses [12, 13]. Keratin 14 (KRT14), a type I intermediate filament protein essential for epithelial integrity, is predominantly expressed in basal epithelial cells [14]. KRT14 is commonly used as a marker of basal-like or squamous differentiation in various cancers, including BLCA, and its expression is linked to aggressive tumor phenotypes and unfavorable prognosis; however, its direct functional contribution to chemoresistance remains poorly understood [15, 16].

Emerging evidence suggests that metabolic reprogramming is a key adaptive strategy that enables cancer cells to survive therapeutic stress [17]. In particular, lipid metabolism has attracted increasing attention, as it affects membrane dynamics, energy storage, and intracellular signaling pathways [18, 19]. Nevertheless, the specific lipid metabolic pathways that are essential for cisplatin resistance in BLCA, as well as their upstream regulatory factors, remain largely undefined. Key metabolic enzymes may be implicated, such as Acyl-CoA Oxidase 2 (ACOX2), which is known for its role in peroxisomal β-oxidation of specific fatty acids and in bile acid synthesis [20]. However, whether ACOX2 has a significant impact on cisplatin resistance in BLCA and the mechanisms underlying its regulation in this context remain to be elucidated [21, 22].

Moreover, precise control of protein synthesis at the level of mRNA translation provides an additional mechanism for rapid cellular adaptation to stress, enabling selective protein expression [23, 24]. While the central roles of core translation initiation factors, such as the eIF4F complex—which is often regulated by oncogenic pathways like mTOR—are well established [25], the contributions of accessory factors and non-canonical molecular interactions in mediating chemoresistance remain less well explored. Eukaryotic translation initiation factor 4H (eIF4H) is one such accessory factor, possessing RNA helicase activity that assists the eIF4F complex in resolving structured 5’ untranslated regions (UTRs) of target mRNAs [26, 27]. How the activity of eIF4H itself might be modulated by unconventional binding partners—such as cytoskeletal elements—to confer selective translational advantages during chemoresistance remains an intriguing and unanswered question [28, 29].

In this study, we uncover a critical mechanism in which KRT14 drives cisplatin resistance in BLCA through direct interaction with the translation factor eIF4H, thereby selectively enhancing the translation of the downstream metabolic enzyme ACOX2. Consequently, targeting this newly identified KRT14–eIF4H–ACOX2 axis may provide new therapeutic strategies and predictive biomarkers for overcoming clinical cisplatin resistance in bladder cancer.

## Materials and methods

### Bioinformatic analysis

RNA-sequencing data and associated clinical statistics for the bladder cancer cohort were obtained from in-house experiments. Raw RNA-seq reads from tumor and adjacent normal tissues, and from cisplatin-resistant and shKRT14 cisplatin-resistant BLCA cell lines, were aligned to the human genome (hg38) using STAR v2.7.9a. Differential expression between tumor versus normal tissues, and between NC versus shKRT14 cells, was analyzed using DESeq2 v1.34.0 in R v4.1.2. Genes with an adjusted P value < 0.05 and |log fold change| > 3 were deemed significantly differentially expressed. KEGG pathway analysis and Gene Set Enrichment Analysis (GSEA v4.2.3, Broad Institute) were conducted using clusterProfiler v4.2.2 in R, and the GSEA tool, respectively, with gene sets from MSigDB v7.5.1.

For survival and correlation analyses, processed RNA-sequencing data (TPM values) and corresponding clinical information for the bladder cancer cohort (TCGA-BLCA) were downloaded from the UCSC Xena platform. Patients were stratified into high- and low-expression groups based on the median expression value of KRT14 or ACOX2. Overall survival (OS) and progression-free survival (PFS) were visualized using Kaplan–Meier curves, and statistical significance was determined using the log-rank test. The correlation between KRT14 and ACOX2 expression was assessed using Pearson’s correlation coefficient. All analyses involving TCGA data were performed in R v4.1.2.

### Quantitative proteomic analysis

Proteomic analysis was performed on three biological replicates of T24-CR cells expressing either shNC or shKRT14. Cell lysates were reduced, alkylated, and digested overnight with trypsin. The resulting peptides were desalted and analyzed on a timsTOF HT mass spectrometer (Bruker) operating in data-independent acquisition (DIA) mode. The raw data were processed using Spectronaut software in library-free mode, searching against the UniProt human database. Protein intensities were normalized, and missing values were imputed. Differentially expressed proteins were identified using a two-tailed Student’s t-test (p < 0.05 and |fold change | ≥ 1.5). Pathway enrichment analyses for Gene Ontology (GO) and KEGG were performed using the clusterProfiler R package.

### Patient samples and immunohistochemistry (IHC)

Formalin-fixed, paraffin-embedded (FFPE) tissue samples from 63 bladder cancer patients. Inclusion criteria stipulated that all patients had histologically confirmed urothelial carcinoma and complete follow-up data. All tissue samples were obtained prior to patients receiving any cisplatin-based chemotherapy. The detailed clinicopathological characteristics of this patient cohort are summarized in Table 1. Sections (4 µm) were deparaffinized in xylene, rehydrated through graded ethanol, and subjected to heat-mediated antigen retrieval in citrate buffer (pH 6.0; Vector Laboratories, H-3300) for 20 min. Endogenous peroxidase activity was quenched with 3% H₂O₂ for 10 min, and sections were blocked with 5% normal goat serum (Vector Laboratories, S-1000) for 1 h at room temperature. Slides were then incubated overnight at 4 °C with primary antibodies against KRT14 (1:200; Abcam, ab181595), ACOX2 (1:150; Proteintech, 10143-1-AP), Ki67 (1:400; Abcam, ab16667), and eIF4H (1:100; CST, 3469). Detection was performed using the VECTASTAIN Elite ABC HRP Kit (Vector Laboratories, PK-6101) and DAB Substrate Kit (Vector Laboratories, SK-4100). After counterstaining with Mayer’s hematoxylin (Sigma-Aldrich, MHS16), slides were mounted and evaluated by two independent pathologists blinded to clinical data. Staining was quantified using the H-score method (intensity 0–3 × percentage of positive cells 0–100).

**Table 1:** Baseline clinicopathological characteristics of patients included in the IHC analysis (N = 63).

Characteristic	Category	Number of patients (%)
Age (years)	Median (range)	71 (42–84)
Sex	Male	54 (85.7%)
	Female	9 (14.3%)
Histological type	Invasive urothelial carcinoma	48 (76.2%)
	Non-invasive urothelial carcinoma	13 (20.6%)
	Other	2 (3.2%)
Clinical stage	Stage 0–I	12 (19.0%)
	Stage II	14 (22.2%)
	Stage III–IV	37 (58.7%)
T stage	T1	16 (25.4%)
	T2	15 (23.8%)
	T3	25 (39.7%)
	T4	7 (11.1%)
N stage	N0 (negative)	45 (71.4%)
	N+ (positive)	9 (14.3%)
	Unknown	9 (14.3%)
M stage	M0 (non-metastatic)	63 (100.0%)
Tumor grade	Low grade	11 (17.5%)
	High grade	52 (82.5%)

### Cell lines and culture

Human bladder cancer cell lines T24 (ATCC HTB-4) and 5637 (ATCC HTB-9) were purchased from ATCC. Cisplatin-resistant sublines (T24-CR and 5637-CR) were generated by exposing parental cells to stepwise increasing concentrations of cisplatin (Sigma-Aldrich, P4394), starting at 0.1 µg/mL and reaching 2 µg/mL over 6–8 months. Both parental and resistant lines were maintained in RPMI-1640 medium (Gibco, 11875093) supplemented with 10% heat-inactivated fetal bovine serum (Gibco, 10099141C) and 1% penicillin–streptomycin (Gibco, 15140122) at 37 °C in a humidified atmosphere containing 5% CO₂. Resistant sublines were cultured in medium containing 2 µg/mL cisplatin, which was removed 48–72 h before experiments unless otherwise indicated. All cell lines were authenticated by short tandem repeat (STR) profiling (ATCC) and routinely screened for mycoplasma contamination using the MycoAlert Mycoplasma Detection Kit (Lonza, LT07-318).

### Plasmid constructs and stable cell line generation

shRNAs targeting human KRT14 and a non-targeting control were cloned into pLKO.1-puro (Addgene #8453). Full-length human KRT14 cDNA (Origene RC201487) was subcloned into pCDH-CMV-MCS-EF1-Puro (System Biosciences CD510B-1) with a C-terminal 3×Flag tag. Domain deletion mutants corresponding to the Head (aa 1–100), Rod (aa 101–430), Tail (aa 431–472), and ΔHead were generated by PCR using specific primers (see Supplementary Table S1) and cloned into the same backbone. Human ACOX2 cDNA (Origene RC202901) was inserted into pCDH-CMV-MCS-EF1-Neo with a C-terminal HA tag, and human eIF4H cDNA (Origene RC201007) was cloned into pCMV-HA (Clontech). The ACOX2 5′ untranslated region was amplified from genomic DNA and ligated into pGL3-basic (Promega E1751). All constructs were verified by Sanger sequencing.

Lentiviral particles were produced by co-transfecting HEK293T cells (ATCC CRL-3216) with the lentiviral plasmid, psPAX2 (Addgene #12260), and pMD2.G (Addgene #12259) using Lipofectamine 3000 (Invitrogen L3000015). Viral supernatants were collected at 48 and 72 h post-transfection, filtered through 0.45 µm membranes, and used to transduce bladder cancer cells in the presence of 8 µg/mL polybrene (Sigma-Aldrich H9268). Stable pools were selected with puromycin (1–2 µg/mL; InvivoGen ant-pr-1) or G418 (500–800 µg/mL; InvivoGen ant-gn-1).

### RNA extraction and quantitative real-time PCR (RT-qPCR)

Total RNA was extracted with TRIzol Reagent (Invitrogen, 15596026) following the manufacturer’s instructions. One microgram of RNA was reverse transcribed using the High-Capacity cDNA Reverse Transcription Kit (Applied Biosystems, 4368814). Quantitative PCR was performed with PowerUp SYBR Green Master Mix (Applied Biosystems, A25742) on a QuantStudio 7 Flex Real-Time PCR System (Applied Biosystems). Relative mRNA levels were calculated by the 2^−ΔΔCT^ method, with GAPDH serving as the internal control. Primer sequences are provided in Supplementary Table S1.

### Protein extraction and Western blotting

Cells were lysed in ice-cold RIPA buffer (CST 9806S) supplemented with Halt Protease and Phosphatase Inhibitor Cocktail (Thermo Fisher Scientific 78440). Protein concentrations were determined by BCA assay (Thermo Fisher Scientific 23225). Equal amounts of protein (20–40 µg) were separated on 4–15% Mini-PROTEAN TGX gels (Bio-Rad 4561086) and transferred to 0.45 µm PVDF membranes (Millipore IPVH00010). Membranes were blocked in TBST containing 5% non-fat dry milk or 5% BSA for 1 h at room temperature, then incubated overnight at 4 °C with primary antibodies: KRT14 (1:1000; Abcam ab181595), ACOX2 (1:1000; Proteintech 10143-1-AP), eIF4H (1:1000; CST 3469), eIF4G (1:1000; CST 2498), eIF4E (1:1000; Proteintech 66655-1-Ig), HA tag (1:2000; Yeasen 30704ES60), Flag tag (1:1000; Sigma-Aldrich F1804), and GAPDH (1:5000; Bioss bsm-33033M). After washing, membranes were incubated for 1 h at room temperature with HRP-conjugated secondary antibodies (anti-rabbit CST #7074 and anti-mouse CST #7076; both 1:3000). Chemiluminescent signals were developed using SuperSignal West Pico PLUS Substrate (Thermo Fisher Scientific 34580) and imaged on a ChemiDoc MP System (Bio-Rad). Band intensities were quantified with Image Lab v6.1 (Bio-Rad). Uncropped blots are provided in the Supplementary Materials.

### Immunofluorescence (IF)

Cells were seeded on glass coverslips (Warner Instruments, 64-0715), fixed in 4% paraformaldehyde (Electron Microscopy Sciences, 15710) for 15 min at room temperature, and permeabilized with 0.25% Triton X-100 (Sigma-Aldrich, T8787) for 10 min. After blocking in PBS containing 3% BSA (Sigma-Aldrich, A7906) for 1 h, coverslips were incubated overnight at 4 °C with primary antibodies against KRT14 (1:200; Abcam, ab181595) and eIF4H (1:100; CST, 3469). The following day, samples were washed and incubated for 1 h at room temperature with Alexa Fluor 488 goat anti-rabbit IgG (Invitrogen, A-11034; 1:500) and Alexa Fluor 594 goat anti-mouse IgG (Invitrogen, A-11032; 1:500). Nuclei were counterstained with DAPI (1 µg/mL; Sigma-Aldrich, D9542), and coverslips were mounted in ProLong Diamond Antifade Mountant (Invitrogen, P36961). Images were acquired on a Leica SP8 confocal microscope using LAS X software v3.5.

### Co-Immunoprecipitation (Co-IP)

Cells were lysed in IP Lysis Buffer (Pierce, 87787) containing protease/phosphatase inhibitors. 1 mg of pre-cleared lysate was incubated with 2–4 µg of antibody (anti-KRT14, anti-eIF4H, anti-Flag M2 (Sigma, F1804), anti-HA (CST, #3724), or normal Rabbit/Mouse IgG (Santa Cruz Biotechnology, sc-2027/sc-2025)) overnight at 4 °C. Protein A/G Magnetic Beads (Thermo Fisher Scientific, 88803) were added for 2 h at 4 °C. The beads were washed four times with IP Lysis Buffer. Bound proteins were eluted with 2× Laemmli sample buffer and analyzed by Western blotting.

### GST pull-down assay

GST, GST-KRT14-FL, and GST-KRT14 fragments were expressed in E. coli BL21(DE3) (Novagen, 69450) and purified on Glutathione Sepharose 4B beads (Cytiva, 17075601). equal quantities of bead-bound GST proteins were incubated with 500 µg of cellular lysate from HEK293T cells overexpressing HA-eIF4H in binding buffer (50 mM Tris-HCl, pH 7.4, 150 mM NaCl, 1 mM EDTA, 0.5% NP-40, protease inhibitors) overnight at 4 °C. Beads were washed five times with binding buffer. bound proteins were eluted and analyzed by Western blotting with anti-HA antibody (CST, #3724, 1:1000).

### Cell viability assay

5000 cells per well were seeded in 96-well plates. After 24 h, cells were treated with indicated concentrations of cisplatin (0–50 µg/mL) for 48 or 72 h. For rescue experiments, cells were co-treated with cisplatin and GW501516 (10 µM; Selleckchem, S2262) or palmitic acid (100 µM complexed with 0.5% fatty acid-free BSA (Sigma, A8806); Sigma, P0500). Cell viability was measured using the Cell Counting Kit-8 (Dojindo, CK04) according to the manufacturer’s instructions. Absorbance was measured at 450 nm on a SpectraMax M5 plate reader (Molecular Devices). IC50 values were calculated the use of GraphPad Prism (v9.3.1).

### Colony formation assay

Cells were seeded at 1000 cells/well in 6-well plates and cultured for 10–14 days, with media changed every 3 days. where indicated, low-dose cisplatin (0.5–1 µg/mL) was included in the medium. Colonies were fixed with methanol, stained with zero.five% crystal violet (Sigma, C0775), and counted.

### Apoptosis assay

Apoptosis was assessed the use of the FITC Annexin V Apoptosis Detection Kit I (BD Biosciences, 556547). After treatment, 1 × 10^6^ cells were harvested, washed, and stained with Annexin V-FITC and Propidium Iodide (PI) consistent with the Kit protocol. Samples were analyzed on a BD LSRFortessa flow cytometer using FACSDiva software (v9.0). Data analysis was performed using FlowJo software (v10.8.1).

### Wound healing assay

Cells were grown to confluence in 6-well plates. A scratch was made with a P200 pipette tip. Debris was washed away, and cells were cultured in RPMI-1640 with 1% FBS. Images were taken at 0 h and 24 h using an EVOS M5000 microscope (Thermo Fisher). Wound area was measured using ImageJ (v1.53t) and percentage closure calculated.

### Transwell migration assay

5 × 10^4^ cells in serum-free medium had been added to the upper chamber of transwell inserts with 8.0 µm pores (Corning, 3422). Medium with 10% FBS was added to the lower chamber. After 24 h, non-migrated cells were eliminated, and migrated cells on the lower floor have been fixed, stained with crystal violet, and counted in 5 random fields per insert.

### Lipid droplet staining

Cells on coverslips were stained with 1 µg/mL Nile red (Invitrogen, N1142) in PBS for 10 min at 37 °C, washed, counterstained with DAPI, and mounted. Images were acquired immediately on a Leica SP8 confocal microscope. Lipid droplet fluorescence depth in line with cell become quantified the use of ImageJ (v1.53t).

### Cholesterol and triglyceride measurement

Intracellular total cholesterol and triglycerides were measured using the Cholesterol Ester Assay Kit (Abcam, ab65359) and Triglyceride Quantification Colorimetric Kit (Abcam, ab65336), respectively, following the manufacturer’s protocols. The intracellular concentration of Human Fatty Acid β-Oxidation Enzyme (FAβ O) was quantified using a commercial ELISA kit (COIBO, CB21069-Hu) according to the manufacturer’s instructions. Briefly, cell lysates were added to the pre-coated microplate, and the concentration of FAβ O was determined by measuring the absorbance at 450 nm. The final values were normalized to the total protein concentration of each sample.

### Cycloheximide (CHX) chase assay

Cells were treated with 200 µg/mL cycloheximide (Sigma, C7698). Cell lysates were collected at 0, 2, 4, and 8 h post-treatment. ACOX2 protein levels had been decided with the aid of Western blotting and quantified relative to time 0.

### Polysome profiling

Cells were treated with 100 µg/mL CHX for 10 min, washed with ice-cold PBS containing CHX, and lysed in polysome buffer (20 mM HEPES-KOH pH 7.4, 100 mM KCl, 5 mM MgCl2, 0.5% NP-40, 100 µg/mL CHX, 1 mM DTT, RNasin Plus RNase Inhibitor (Promega, N2611)). Lysates (approx. 10 A260 devices) were layered onto 10-50% linear sucrose gradients and centrifuged at 39,000 rpm for 2 h at 4 °C in an SW41Ti rotor (Beckman Coulter). Gradients were fractionated using an ISCO Density Gradient Fractionation machine, tracking A254nm. RNA from fractions turned into remoted the usage of TRIzol LS and analyzed by RT-qPCR for ACOX2 mRNA distribution.

### Luciferase reporter assay

HEK293T cells were seeded in 24-well plates and co-transfected using Lipofectamine 3000 with pGL3-ACOX2-5′UTR (100 ng), pRL-TK Renilla control vector (10 ng), and expression vectors for KRT14-WT/MUT (200 ng) or sh-eIF4H/shNC (200 ng). 48 h post-transfection, luciferase activity was measured using the Dual-Glo Luciferase Assay System (Promega, E2920) on a GloMax Discover luminometer (Promega). Firefly activity was normalized to Renilla activity.

### RNA immunoprecipitation (RIP) assay

RIP was performed using the Magna RIP Kit (Millipore, 17-700). 1 × 10^7^ cells were lysed, and 100 µL lysate was incubated with 5 µg of anti-KRT14 (Abcam, ab7800), anti-eIF4H (Abcam, ab154186), or Rabbit IgG control antibody complexed with Protein A/G magnetic beads. After overnight incubation and washes, RNA was extracted. Co-precipitated ACOX2 mRNA was quantified by RT-qPCR and expressed as fold enrichment over IgG relative to input.

### RNA pull-down assay

Biotinylated ACOX2 5′UTR RNA probe was transcribed in vitro the use of Biotin RNA Labeling mix (Roche, 11685597910) and T7 polymerase (Promega, P2075) from a PCR template. 2 µg of biotinylated RNA turned into incubated with 1 mg of mobile lysate for 4 h at 4 °C. Complexes have been captured the use of Dynabeads M-280 Streptavidin (Invitrogen, 11205D) for 1 h. Beads had been washed, and bound proteins were eluted and analyzed by way of Western blotting for KRT14 and eIF4H.

### Global protein synthesis assay (SUnSET)

To measure the rate of global protein synthesis, a non-radioactive SUnSET (Surface Sensing of Translation) assay was performed. T24-CR cells stably expressing the control vector (NC) or overexpressing KRT14 (KRT14 OE) were seeded in 6-well plates and grown to approximately 80% confluency. The culture medium was then replaced with fresh medium containing 10 µg/mL puromycin (Sigma-Aldrich, P8833) and incubated for exactly 10 min at 37 °C. For a positive control, control cells were serum-starved overnight and then stimulated with 20% FBS for 1 h prior to the puromycin pulse. Following incubation, cells were immediately placed on ice, washed twice with ice-cold PBS containing 100 µg/mL cycloheximide (Sigma, C7698), and then lysed in RIPA buffer. The incorporation of puromycin into nascent polypeptide chains was detected by Western blotting using a specific anti-puromycin antibody (Millipore, MABE343; 1:10,000).

### Animal model

#### In vivo xenograft studies

Female athymic nude mice (BALB/c; 5–6 weeks old; Charles River Laboratories) were employed. 5 × 10^6^ cells in 100 µL 1:1 PBS/Matrigel (Corning, 356234) were subcutaneously injected into the right flank (n = 8–10 mice/group). Upon reaching a volume of ~100 mm³, mice were randomized into treatment groups of similar average tumor volume and treated with vehicle (PBS) or cisplatin (3 mg/kg; intraperitoneal injection; twice a week) for 3–4 weeks. Body weight and volume of tumors (V = 0.5 × L × W²) were measured every 3 days. At endpoint, tumors were weighed, photographed, and halved for IHC or snap-frozen for biochemical analysis. All mice that developed tumors were included in the final analysis.

### Orthotopic bladder cancer models

An orthotopic bladder cancer model was established using the intravesical instillation method. Briefly, T24 or T24-CR cells engineered to express luciferase were used. Mice were anesthetized with isoflurane, and a lubricated polyethylene catheter was gently inserted through the urethra into the bladder. After emptying the residual urine, the bladder epithelium was mildly preconditioned with poly-L-lysine (0.1 mg/mL) for 15 min to facilitate tumor cell adherence. Subsequently, 5 × 10⁵ bladder cancer cells in 50 µL PBS were instilled into the bladder cavity and retained for 30 min by temporarily clamping the urethra. Tumor establishment was confirmed 1 week later by bioluminescence imaging (BLI). Only mice with successfully established tumors (positive BLI signal) were included in the final analysis. These mice were then randomly assigned to the treatment groups. All orthotopic experimental data shown are from groups with a final sample size of n = 5 mice per group.

### Clinical imaging snapshot

#### Patients and ethics

Six histologically proven urothelial-carcinoma cases were reviewed under IRB approval (Approval No.22KN209) with written informed consent. (Case 1: 71-year-old male with locally advanced left renal pelvis tumor; Case 2: 68-year-old male with local pelvic recurrence after radical cystectomy; Case 3: 70-year-old male with muscle-invasive bladder cancer undergoing neoadjuvant platinum-based chemotherapy; Case 4: 65-year-old male with muscle-invasive bladder cancer receiving neoadjuvant chemotherapy; Case 5: 79-year-old female with metastatic bladder cancer; Case 6: 70-year-old male with inguinal lymph node metastasis post-radical cystectomy.)

#### Chemotherapy

All patients received standard gemcitabine-cisplatin combination therapy (gemcitabine 1 g m^−^² days 1/8 + cisplatin 70 mg m⁻² day 1, q21d) and were given for three or four cycles.

#### CT imaging

Target lesions were assessed via CT or MRI obtained before treatment and within 2 weeks after the final treatment.

#### Response assessment

Two blinded radiologists independently evaluated the lesions and applied RECIST v1.1 criteria. Cases 1–3 showed no significant regression or progression after platinum-based chemotherapy, while cases 4–6 demonstrated marked reduction in target lesions. Representative axial slices were exported from PACS and annotated with red arrows (Adobe Illustrator 2024).

### Statistical analysis

All data are presented as the mean ± SD from at least three independent experiments unless otherwise stated. Statistical analyses were performed using GraphPad Prism (v9.3.1; GraphPad Software). Prior to parametric testing, data were assessed for normal distribution and homogeneity of variances. The two-tailed unpaired Student’s t-test was used for comparisons between two groups. One-way or two-way ANOVA with Tukey’s or Sidak’s multiple comparisons test was used for multiple group comparisons. The Pearson correlation coefficient was calculated for correlation assessment. Kaplan–Meier survival analysis was performed using the log-rank test. A P value < 0.05 was considered statistically significant (*P < 0.05, **P < 0.01, ***P < 0.001).

## Results

### Upregulation of KRT14 drives cisplatin resistance in bladder cancer

**Fig. 1:**
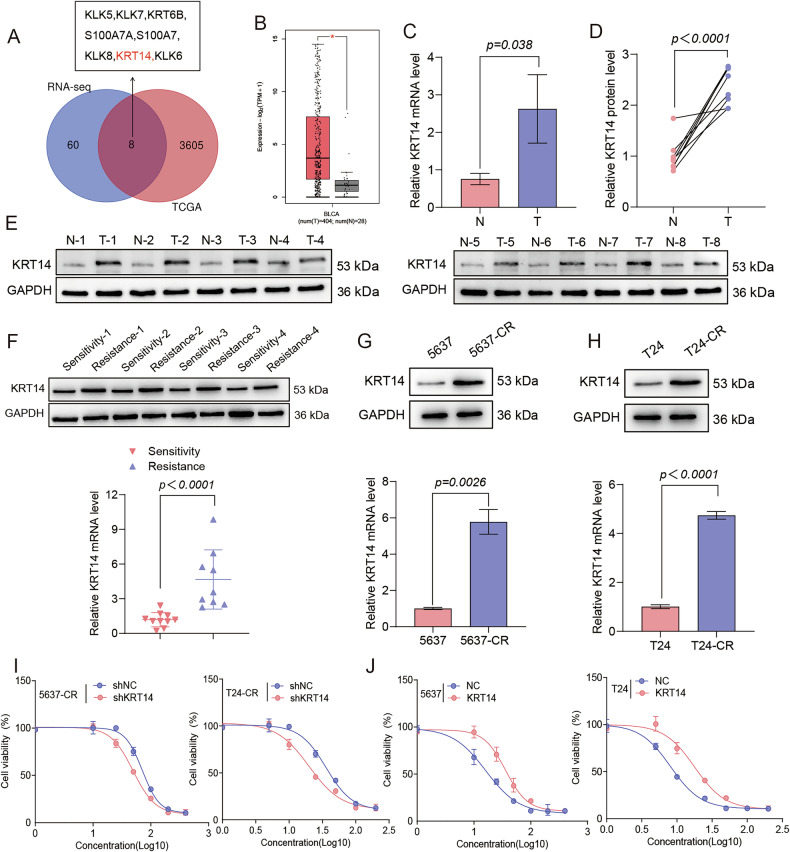
KRT14 is upregulated in BLCA, correlates with cisplatin resistance, and dictates drug sensitivity. **A** Venn diagram showing the overlap of significantly upregulated genes identified from two independent bladder cancer cohorts. The left circle represents differentially expressed genes from our in-house RNA-seq analysis (Tumor vs. Adjacent Normal Tissues), and the right circle represents those from the TCGA-BLCA dataset (Tumor vs. Normal Tissues). The eight commonly upregulated genes are listed. **B** KRT14 mRNA expression (TCGA-BLCA; log2(TPM + 1)) in normal (N, n = 28) vs. tumor (T, n = 404) tissues. ***P < 0.0001 (Wilcoxon rank-sum test). Box: IQR, line: median. **C** RT-qPCR of KRT14 mRNA (normalized to GAPDH) in paired N and T tissues (n = 3). P = 0.038 (Paired t-test). **D** Relative KRT14 protein quantification (WB normalized to GAPDH) in paired N/T tissues (n = 6). P < 0.0001 (Paired t-test). **E** Representative WB showing KRT14 protein in 8 paired N/T samples. **F** Top: Representative WB of KRT14 in cisplatin-sensitive vs. -resistant patient tumors. Bottom: RT-qPCR of KRT14 mRNA (normalized to GAPDH) in sensitive (n = 4) vs. resistant (n = 4) tumors. P < 0.0001 (Student’s t-test). Baseline KRT14 levels in parental vs. resistant (CR) cells. WB (Top) and RT-qPCR (Bottom, normalized to GAPDH) for 5637 (**G**) and T24 (**H**) lines. P-values shown (Student’s t-test). **I** Cisplatin viability curves (CCK-8, 48 h) for resistant 5637-CR and T24-CR cells expressing shNC or shKRT14. Knockdown increases sensitivity. **J** Cisplatin viability curves (CCK-8, 48 h) for parental 5637 and T24 cells expressing NC or KRT14. Overexpression decreases sensitivity.

Cisplatin resistance remains a formidable barrier to the effective treatment of BLCA [10]. To uncover the molecular drivers underpinning this resistance, we first sought to identify genes fundamentally dysregulated during tumorigenesis. RNA-sequencing profiles from both our in-house patient cohort and the public TCGA-BLCA dataset consistently identified KRT14 as one of the most significantly upregulated genes in tumor tissues compared to adjacent normal tissues (Fig. 1A, B). This finding was validated at both the mRNA and protein levels in our patient cohort, where KRT14 was markedly elevated in tumors (Fig. 1C–E). Given that KRT14 upregulation was robustly confirmed, and considering the role of keratins in maintaining cell integrity and stress responses, we hypothesized that KRT14 may be involved in mediating chemoresistance, a major clinical challenge in BLCA. Indeed, analysis of patient samples revealed a strong clinical correlation: KRT14 mRNA and protein levels were significantly higher in tumors from patients exhibiting resistance to cisplatin compared to those who remained sensitive (Fig. 1F). These data strongly implicated KRT14 upregulation as a potential driver of chemoresistance.

To mechanistically investigate this clinical correlation, we first established cisplatin-resistant BLCA cell lines, which, consistent with the patient data, displayed markedly higher endogenous levels of KRT14 mRNA and protein than their parental, sensitive counterparts (Figs. 1G, H and S1A). Having established this clinically relevant model, we directly tested if KRT14 was functionally responsible for the resistant phenotype. Indeed, shRNA-mediated silencing of KRT14 in the resistant 5637-CR and T24-CR cell lines was sufficient to fully restore their sensitivity to cisplatin, as demonstrated by significantly reduced cell viability and impaired colony-forming ability under drug treatment (Figs. 1I and S1B, C, F). Conversely, we performed the gain-of-function experiment. Ectopic overexpression of KRT14 in the parental, cisplatin-sensitive 5637 and T24 cells was sufficient to confer a robustly resistant phenotype, significantly decreasing their sensitivity to cisplatin-induced cell death (Figs. 1J and S1D, E). Taken together, these loss- and gain-of-function experiments establish that KRT14 is not merely correlated with, but is a key functional driver of, cisplatin resistance in BLCA cells.

### Upregulated KRT14 fuels BLCA malignancy and confers cisplatin resistance

**Fig. 2:**
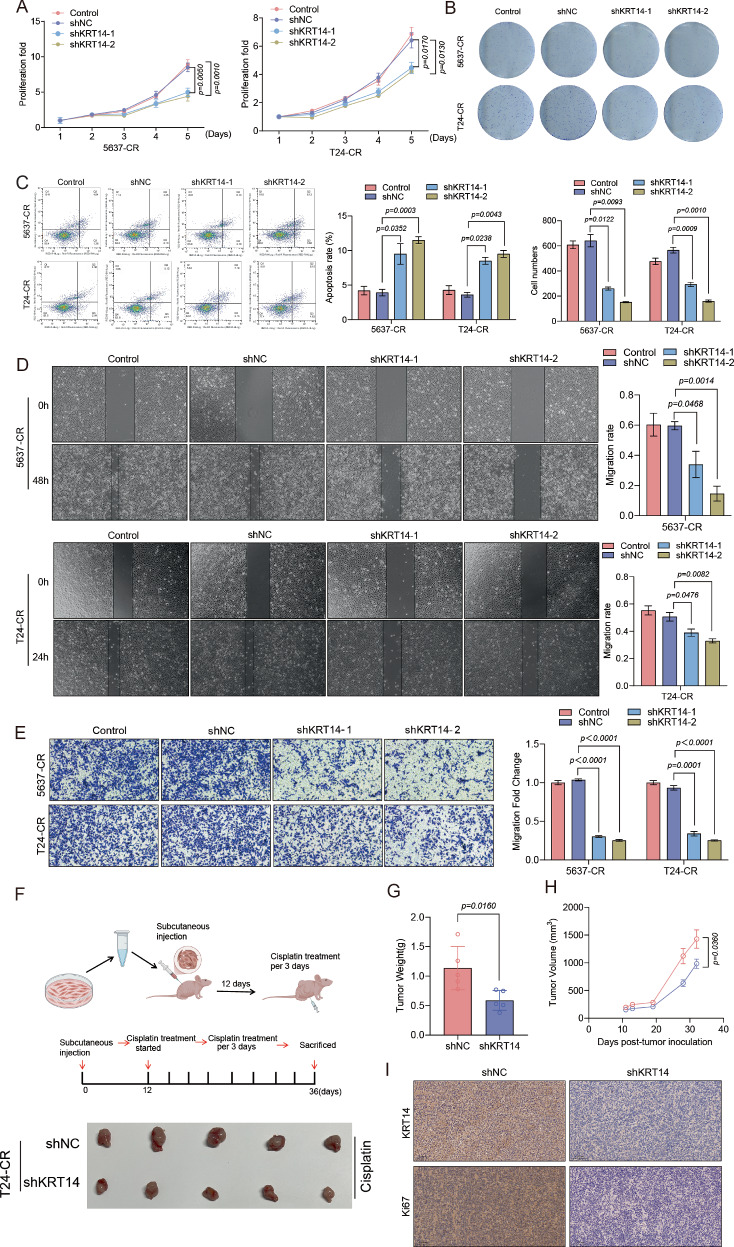
KRT14 knockdown inhibits malignant traits and enhances in vivo cisplatin efficacy. Resistant 5637-CR and T24-CR cells expressing Control, shNC, or shKRT14-1/-2 were assessed. **A** Proliferation rates (CCK-8, 5 days). **B** Representative colony formation assays (14 days). **C** Apoptosis assessed by Annexin V/PI flow cytometry. Representative plots (Left) and quantification (Right). **D** Wound healing assay. Representative images (0 h, 24/48 h; Scale bar = 200 µm) and migration rate quantification. **E** Transwell migration assay (24 h). Representative images (Magnification = 100×) and quantification. **F** T24-CR subcutaneous xenograft model schematic (Top) and representative excised tumors (Bottom) from mice treated with cisplatin (3 mg/kg, twice weekly) after shNC/shKRT14 cell injection. **G** Final tumor weights (n = 5 mice/group) from experiment (**F**). **H** Tumor growth curves (n = 5 mice/group) from experiment (**F**). **I** Representative IHC staining for KRT14 and Ki67 in xenograft tumors from (**F**). Magnification = 200×.

Having established KRT14 as a key driver of cisplatin resistance, we next sought to determine its impact on other core oncogenic properties. To this end, we silenced KRT14 in the resistant 5637-CR and T24-CR cell lines. KRT14 depletion significantly impaired cellular proliferation (Fig. 2A) and markedly reduced their long-term colony-forming ability (Fig. 2B). Furthermore, consistent with a pro-survival role, KRT14 knockdown also rendered these resistant cells more susceptible to apoptosis (Fig. 2C). Given that pathways driving chemoresistance often confer metastatic potential [30], we investigated whether KRT14 also promotes cell motility. Indeed, KRT14 silencing substantially impaired cell migration in both wound healing and transwell assays (Fig. 2D, E), indicating an additional role for KRT14 in promoting an invasive phenotype. Conversely, ectopic overexpression of KRT14 in parental, non-resistant cells was sufficient to confer these aggressive cellular behaviors, including enhanced proliferation, survival, and migration (Fig. S2A–E). Collectively, these data establish that KRT14 is not only necessary for maintaining the malignant state of resistant cells but is also sufficient to drive these aggressive phenotypes.

To determine whether the pivotal role of KRT14 in driving malignancy in vitro translates to a tangible impact on tumor growth and therapeutic response in vivo, we established a subcutaneous xenograft model. T24-CR cells stably expressing either shKRT14 or a control shRNA were implanted into immunodeficient mice, which were subsequently treated with cisplatin (Fig. 2F, schematic). Consistent with our in vitro data, targeting KRT14 in vivo dramatically suppressed tumor growth and enhanced sensitivity to cisplatin. This was manifested by a visibly reduced tumor burden (Fig. 2F), a significant reduction in final tumor weight (Fig. 2G), and markedly attenuated tumor progression kinetics over the course of treatment (Fig. 2H). Immunohistochemical analysis of excised tumors provided a direct molecular correlate for these observations, confirming effective KRT14 knockdown and revealing a concomitant, significant reduction in the proliferation marker Ki67 in the shKRT14 group (Fig. 2I). To corroborate these findings in a more physiologically relevant setting that mimics the native tumor microenvironment, we further established an orthotopic bladder cancer model. Consistent with our subcutaneous xenograft data, KRT14 knockdown significantly reduced the primary tumor burden, as measured by bioluminescence imaging. Critically, this model also revealed that silencing KRT14 dramatically suppressed the formation of distant lung metastases (Fig. S2F–J). These in vivo findings demonstrate that targeting KRT14 can suppress BLCA tumor growth and overcome chemoresistance, validating its potential as a therapeutic target.

### KRT14 drives cisplatin resistance by reprogramming cellular lipid metabolism

**Fig. 3:**
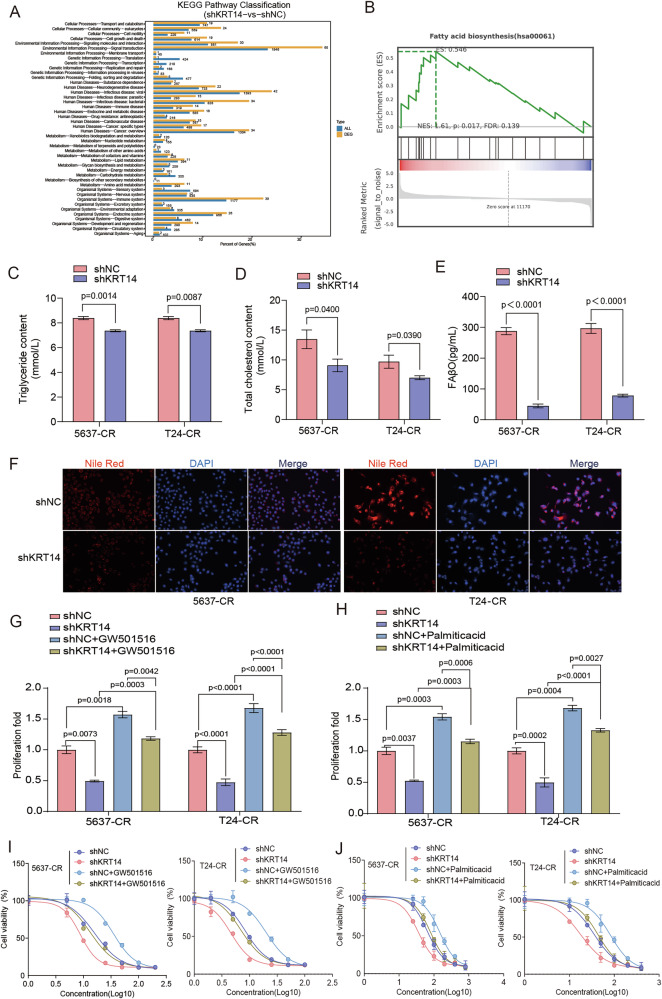
KRT14 modulates lipid metabolism; targeting metabolism rescues shKRT14 phenotypes. **A** KEGG pathway analysis (RNA-seq: shKRT14 vs. shNC resistant cells) highlighting altered metabolic pathways. **B** GSEA showing negative enrichment of Fatty Acid Biosynthesis pathway (hsa00061) in shKRT14 cells. NES, p, FDR indicated. Quantification of intracellular Triglycerides (**C**), Cholesterol (**D**), and FAβO marker (**E**) in resistant cells (shNC vs. shKRT14), normalized to protein. Knockdown reduces lipid components. **F** Representative Nile Red staining showing reduced lipid droplets in shKRT14-resistant cells. Scale bar = 50 µm. **G**, **H** Proliferation rescue (CCK-8, 72 h). Resistant cells (shNC/shKRT14) treated with GW501516 (10 µM, **G**) or Palmitic acid (100 µM, **H**). Both partially rescue shKRT14 effect. **I**, **J** Cisplatin viability rescue (CCK-8, 48 h). Resistant cells (shNC/shKRT14) ± GW501516 (**I**) or Palmitic acid (**J**). Both partially reverse shKRT14-induced sensitization. Data in (**C**–**E**, **G**–**J**) are mean ± SD (n = 3). P-values indicated.

To uncover the molecular mechanism governed by KRT14 that contributes to cisplatin resistance, we performed a transcriptomic analysis comparing control (shNC) and KRT14-depleted (shKRT14) resistant cells. Pathway analysis of the differentially expressed genes revealed that KRT14 knockdown induced significant alterations in metabolic pathways, particularly those related to lipid metabolism (Fig. 3A). Specifically, GSEA showed a significant negative enrichment of the fatty acid biosynthesis pathway in shKRT14 cells (Fig. 3B), suggesting that high KRT14 expression sustains a lipogenic phenotype. To directly test this hypothesis, we first confirmed that cisplatin-resistant cells (5637-CR, T24-CR) indeed harbor a high-lipid state, exhibiting significantly elevated levels of total cholesterol and triglycerides compared to their sensitive parental counterparts (Fig. S3A, B). The clinical relevance of this observation is underscored by public data analyses, which show a distinct lipid metabolism gene signature associated with high KRT14 expression in patients, and confirm KRT14 upregulation in independent resistant cell line models (Fig. S3C, D). We then assessed the direct impact of KRT14 on this phenotype. Silencing KRT14 in resistant cells markedly reduced intracellular levels of triglycerides (Fig. 3C), total cholesterol (Fig. 3D), and a key marker of fatty acid β-oxidation (FAβ O) (Fig. 3E). These biochemical findings were visually confirmed by Nile Red staining, which demonstrated a dramatic reduction in intracellular lipid droplets upon KRT14 knockdown (Fig. 3F). Collectively, these data establish that KRT14 is essential for maintaining the reprogrammed, high-lipid metabolic state of cisplatin-resistant cells.

Having established this KRT14-lipid metabolism link, we next performed a series of rescue experiments to determine if this metabolic function is the key mediator of KRT14’s pro-survival effects. We first assessed proliferation. As previously shown, KRT14 knockdown inhibited the proliferation of resistant cells; however, this anti-proliferative effect was significantly counteracted by either activating lipid metabolism with the PPARδ agonist GW501516 (Fig. 3G) or by directly supplementing the culture with palmitic acid (Fig. 3H). Most critically, we tested if this metabolic rescue could also overcome cisplatin sensitivity. Indeed, while KRT14 depletion re-sensitized resistant cells to cisplatin, this sensitization was markedly attenuated by treatment with either GW501516 (Fig. 3I) or palmitic acid (Fig. 3J). Taken together, these rescue experiments provide compelling evidence that KRT14 drives both cell proliferation and cisplatin resistance in BLCA primarily through its capacity to sustain a reprogrammed, lipogenic state.

To further validate this dependency using a pharmacological approach, we tested whether inhibiting a key lipogenic enzyme could reverse the resistance conferred by KRT14 overexpression. We utilized TVB-2640, a specific inhibitor of Fatty Acid Synthase (FASN) [31], and performed co-treatment experiments with cisplatin. Consistent with our findings, KRT14 overexpression in both T24 and 5637 cells resulted in significant cisplatin resistance (Fig. S3E). Strikingly, co-treatment with TVB-2640 substantially reversed this phenotype, markedly re-sensitizing the KRT14-overexpressing cells to cisplatin to a level comparable to that of control cells. This pharmacological evidence strongly reinforces the conclusion that the chemoresistance driven by KRT14 is functionally dependent on its ability to reprogram lipid metabolism, highlighting this pathway as a critical therapeutic vulnerability.

### KRT14 regulates cisplatin resistance and malignant phenotypes through the downstream effector ACOX2

**Fig. 4:**
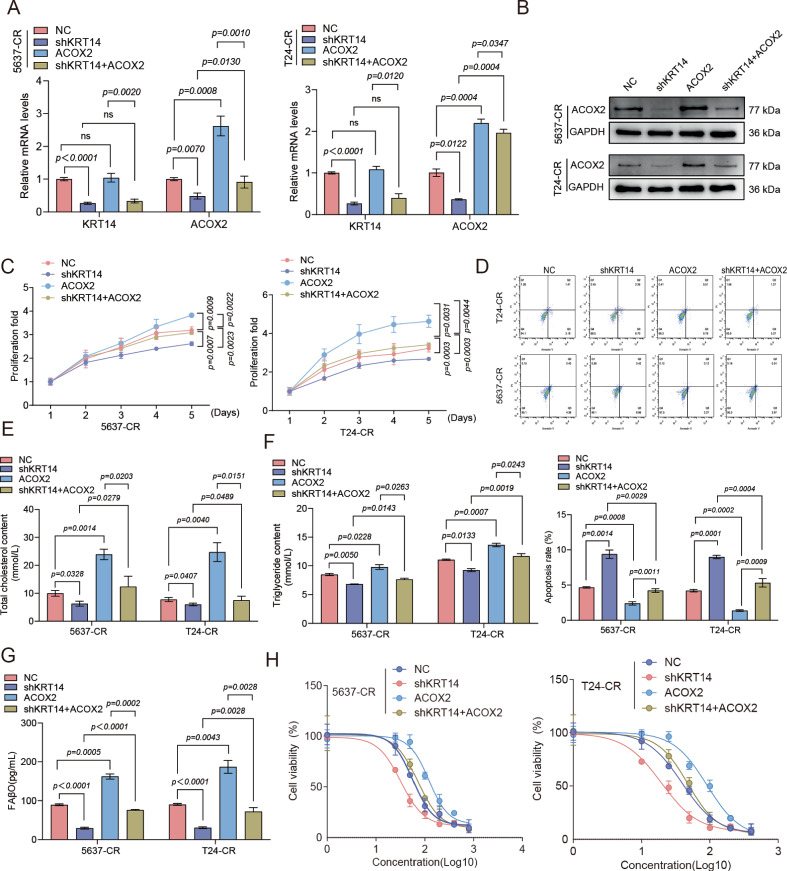
ACOX2 mediates KRT14’s pro-tumorigenic and resistance effects. Resistant 5637-CR /T24-CR cells expressed NC, shKRT14, ACOX2 OE, or shKRT14 + ACOX2 OE. **A** RT-qPCR validation of KRT14 KD and ACOX2 OE. **B** WB validation showing ACOX2 OE rescues KD effect on ACOX2 protein. **C** Proliferation curves (CCK-8, 5 days). ACOX2 OE partially rescues shKRT14 defect. **D** Apoptosis (Annexin V/PI flow cytometry). Representative plots and quantification. ACOX2 OE partially rescues shKRT14 effect. Quantification of Cholesterol (**E**), Triglycerides (**F**), and FAβO marker (**G**). ACOX2 OE partially rescues shKRT14 effects. **H** Cisplatin viability curves (CCK-8, 48 h). ACOX2 OE partially rescues shKRT14-induced sensitization.

To unravel the precise molecular mechanism by which KRT14 reprograms lipid metabolism, we returned to our transcriptomic data to identify critical downstream effectors. We specifically interrogated genes related to lipid metabolism that were downregulated upon KRT14 silencing (Fig. S4A, B). Among these candidates, ACOX2 emerged as the most robustly suppressed gene following KRT14 knockdown (Fig. S4C). Consistent with a potential role in the resistant phenotype, ACOX2 expression, like that of KRT14, was markedly elevated in cisplatin-resistant cells compared to their sensitive counterparts (Fig. S4D). We first confirmed the efficiency of our manipulations, verifying effective KRT14 knockdown and robust ACOX2 overexpression at both the mRNA (Figs. 4A and S4E) and protein levels (Figs. 4B and S4F). This established a clear KRT14-ACOX2 regulatory axis. We next performed rescue experiments to place this axis in a functional hierarchy. Critically, while KRT14 depletion suppressed proliferation and induced apoptosis, these effects were substantially reversed by the ectopic expression of ACOX2 (Fig. 4C, D).

Having established ACOX2 as a key downstream mediator of KRT14’s pro-survival effects, we next sought to determine if it was also responsible for the observed metabolic reprogramming. As shown previously, KRT14 depletion led to a marked decrease in cellular cholesterol, triglycerides, and FAβO. Remarkably, this entire lipid-depleted phenotype was fully rescued by the concurrent overexpression of ACOX2 (Fig. 4E–G). firmly establishing ACOX2 as a necessary mediator of KRT14’s effects on cellular lipid profiles. Further supporting ACOX2’s direct role, specific ACOX2 knockdown reduced lipid droplets (Fig. S4G), and notably, this effect persisted even in the context of KRT14-driven lipid accumulation (as in “KRT14+shNC” vs. “KRT14+shACOX2”; Fig. S4H), highlighting ACOX2’s essential downstream function in lipid metabolism. Finally, to determine whether the KRT14-ACOX2-lipid metabolism pathway converges on chemoresistance, we examined cellular responses to cisplatin. As established, KRT14 knockdown sensitized resistant cells to the drug (Fig. 4H). Strikingly, the increased sensitivity induced by KRT14 silencing was significantly attenuated by concurrent overexpression of ACOX2, partially restoring the chemoresistant phenotype (Fig. 4H). Taken together, KRT14, which is upregulated in resistant cells, appears to sustain high levels of ACOX2, and this KRT14-ACOX2 axis is instrumental in driving not only cell proliferation and survival, but also the specific lipid metabolic profile associated with, and ultimately contributing to, cisplatin resistance in bladder cancer.

### KRT14 promotes ACOX2 translation by directly interacting with eIF4H and recruiting it to the ACOX2 5′UTR

**Fig. 5:**
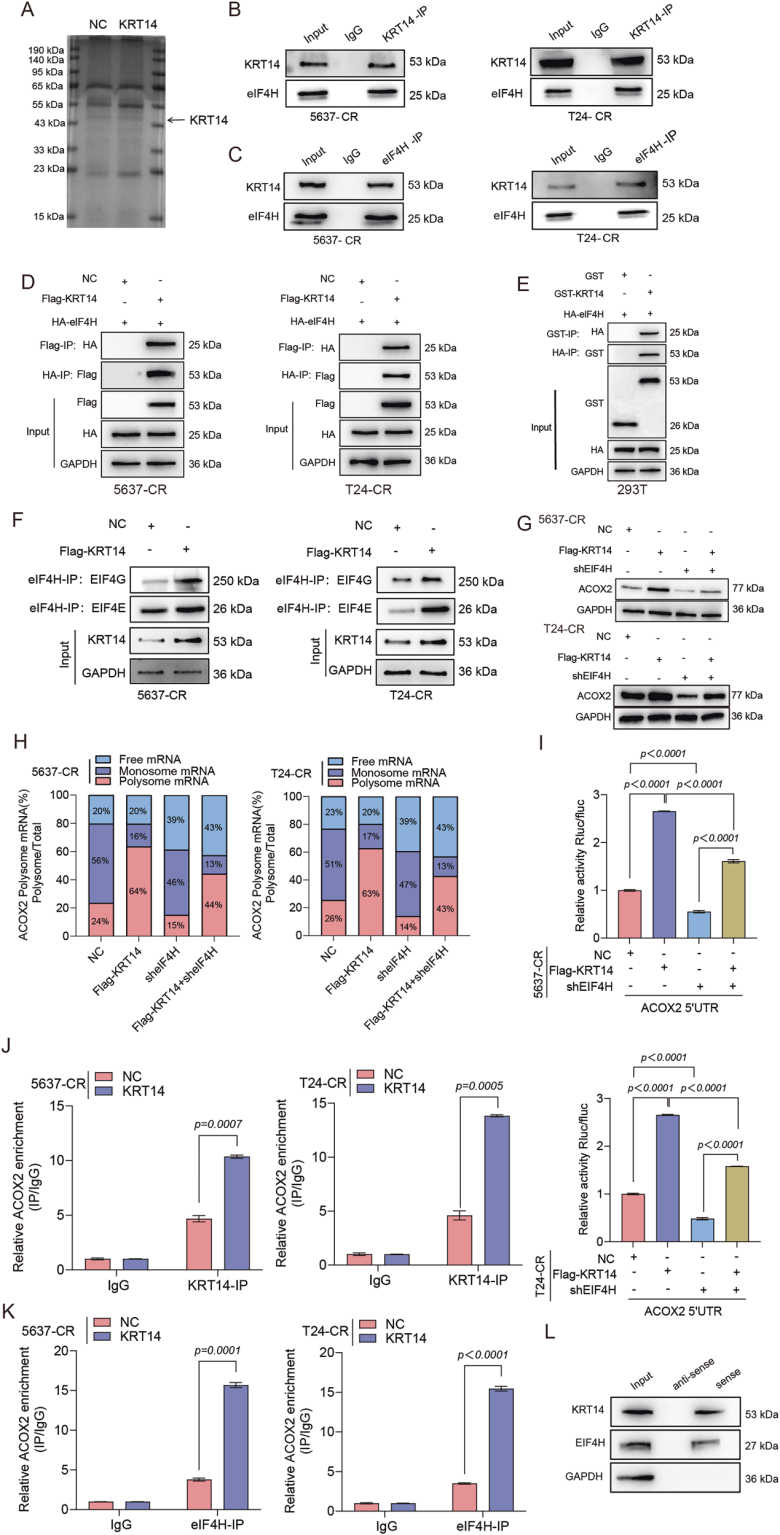
KRT14 physically interacts with eIF4H and enhances ACOX2 translation. **A** Coomassie blue stain comparing lysates (NC vs. KRT14 OE 5637- CR cells). Arrow ~KRT14. **B**, **C** Reciprocal endogenous Co-IP in resistant cells confirms KRT14–eIF4H interaction. WB for indicated proteins. **D** Reciprocal Co-IP confirms interaction of co-expressed Flag-KRT14 and HA-eIF4H. **E** In vitro GST pull-down showing HA-eIF4H binds GST-KRT14. WB for HA/GST. **F** Co-IP (eIF4H-IP) shows KRT14 OE enhances eIF4H interaction with eIF4G/E in resistant cells. **G** WB shows KRT14 OE increases ACOX2 protein, effect attenuated by shEIF4H. **H** Polysome profiling shows KRT14 OE shifts ACOX2 mRNA to heavier polysomes, partially reversed by shEIF4H. Quantification of mRNA distribution (%). **I** Dual-luciferase assay (ACOX2 5′UTR reporter) shows KRT14 stimulates activity via eIF4H. RIP-qPCR shows KRT14 OE increases ACOX2 mRNA association with KRT14 (**J**) and eIF4H (**K**). **L** RNA pull-down using biotinylated ACOX2 5′UTR sense probe captures KRT14 and eIF4H from lysate.

To elucidate the molecular mechanism by which KRT14 regulates ACOX2, we first sought to identify its direct interacting partners. An initial screen combining SDS-PAGE with mass spectrometry (MS) identified the translation initiation factor eIF4H as a prominent KRT14-associated protein (Fig. 5A). We rigorously validated this interaction through multiple lines of evidence. Reciprocal Co-IP assays demonstrated a robust, in vivo association between endogenous KRT14 and eIF4H in resistant BLCA cells (Fig. 5B, C). This finding was confirmed using tagged, overexpressed proteins (Fig. 5D). To determine if this association was direct, we performed in vitro GST pull-down assays with purified recombinant proteins, which confirmed a direct physical interaction between KRT14 and eIF4H (Fig. 5E). Consistent with this binding, immunofluorescence analysis revealed substantial co-localization of KRT14 and eIF4H within the cytoplasm (Fig. S5A).

Given that eIF4H is a component of the eIF4F translation initiation complex [32], we hypothesized that KRT14 might regulate ACOX2 at the translational level. We first confirmed that KRT14 overexpression enhances the association between eIF4H and other core components of the complex, such as eIF4G and eIF4E, suggesting a functional modulation (Fig. 5F). We next investigated the functional consequence of this interaction on ACOX2 expression. Using shRNA-mediated knockdown, we first confirmed efficient silencing of eIF4H at the mRNA level (Fig. S5B). Next, to test the functional hierarchy, we found that eIF4H is necessary for ACOX2 expression, as its depletion alone markedly reduced ACOX2 protein levels (Fig. S5C). Critically, while KRT14 overexpression robustly increased ACOX2 protein, this effect was significantly abrogated by the concurrent knockdown of eIF4H (Fig. 5G), establishing that KRT14 requires eIF4H to elevate ACOX2 protein abundance. CHX chase assays further revealed that this regulation occurs at the level of protein synthesis, as KRT14 overexpression did not alter ACOX2 degradation (Fig. S5D). This finding ruled out post-translational stability as the primary mechanism and strongly suggested that KRT14 enhances ACOX2 abundance by promoting its synthesis.

To provide definitive evidence for a translational control mechanism, we performed polysome profiling. This analysis revealed that KRT14 overexpression caused a distinct shift of endogenous ACOX2 mRNA into actively translating, heavy-polysome fractions, an effect that was reversed upon eIF4H depletion (Fig. 5H). This demonstrates that KRT14 enhances ACOX2 translation in an eIF4H-dependent manner. We then pinpointed the *cis*-regulatory element responsible. Luciferase reporter assays showed that KRT14 specifically stimulated translation through the ACOX2 5′ untranslated region (5’UTR), and this effect was strictly dependent on eIF4H (Fig. 5I). Finally, to prove that KRT14 and eIF4H form a physical complex on the target mRNA, we performed RNA-protein interaction assays. RIP and RNA pull-down assays both provided direct evidence that KRT14 promotes the assembly of a specific KRT14–eIF4H ribonucleoprotein (RNP) complex on the endogenous ACOX2 mRNA (Fig. 5J–L). Collectively, KRT14 physically recruits eIF4H to the ACOX2 5’UTR, boosting its translational efficiency and thereby elevating the protein levels required to drive the malignant phenotype.

### The head domain of KRT14 is essential for interaction with eIF4H and mediating downstream effects

**Fig. 6:**
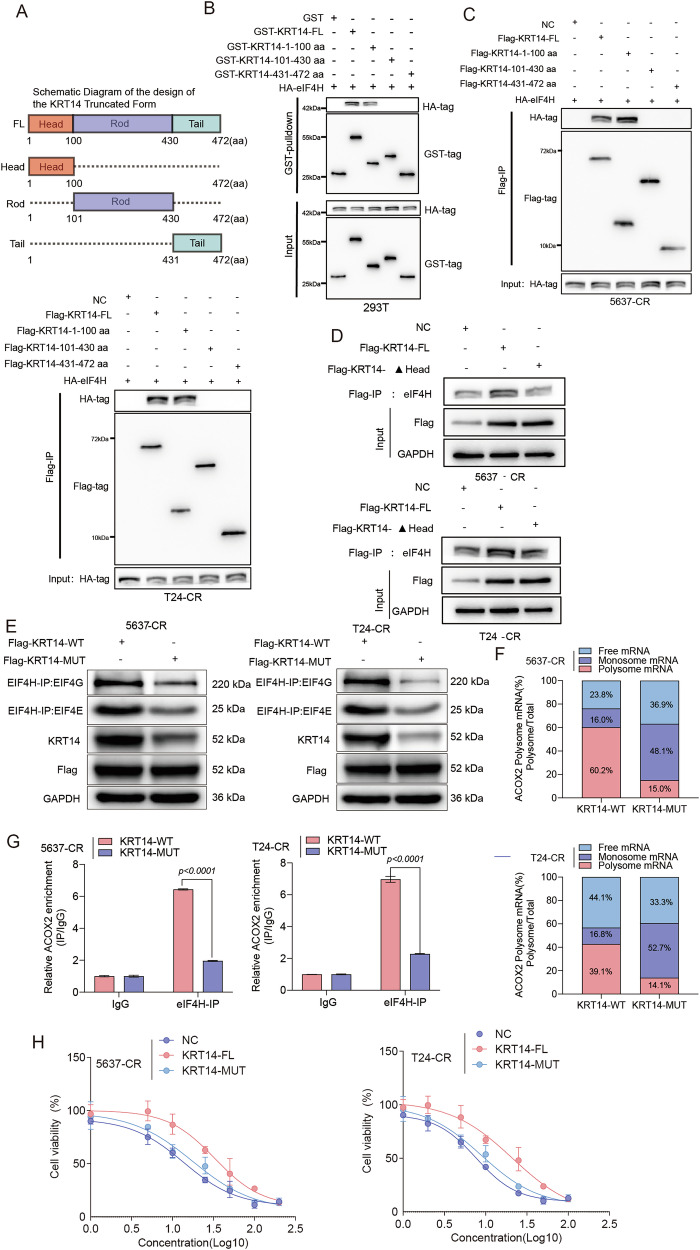
KRT14 head domain is crucial for eIF4H interaction and function. **A** Schematic of KRT14-FL and truncated domains (Head, Rod, Tail). **B** GST pull-down shows HA-eIF4H binds KRT14-FL and KRT14-Head fragments in vitro. **C** Co-IP shows HA-eIF4H co-precipitates with Flag-KRT14-FL and -Head fragments in cells. **D** Co-IP shows endogenous eIF4H fails to co-precipitate with Flag-KRT14-ΔHead. **E**–**H** Functional comparison of KRT14-WT (FL) vs. KRT14-MUT (ΔHead) in resistant cells. **E** Co-IP (eIF4H-IP) shows MUT fails to enhance eIF4H-eIF4G/E interaction. **F** Polysome profiling shows MUT fails to enhance ACOX2 mRNA polysome loading. **G** RIP-qPCR shows MUT fails to enhance eIF4H binding to ACOX2 mRNA. **H** Cisplatin viability curves (CCK-8, 48 h) show MUT fails to confer resistance.

To further dissect the molecular basis of the KRT14–eIF4H interaction, we performed a domain mapping analysis to pinpoint the specific region of KRT14 required for this binding. Guided by UniProt annotations, we engineered a series of truncation mutants corresponding to the canonical Head, Rod, and Tail domains of KRT14 (Fig. 6A). First, in vitro GST pull-down assays using purified KRT14 fragments revealed that eIF4H binding was exclusively localized to the N-terminal Head domain (amino acids 1–100); in contrast, the Rod and Tail domains failed to show any significant interaction (Fig. 6B). We then confirmed this finding in a cellular context. Reciprocal Co-IP assays in resistant BLCA cells demonstrated that only full-length KRT14 and the Head domain fragment, but not the Rod or Tail, were capable of associating with eIF4H (Fig. 6C). Finally, to definitively establish the necessity of this region, we showed that a KRT14 deletion mutant lacking the Head domain (KRT14-ΔHead) was completely unable to bind endogenous eIF4H (Fig. 6D). Collectively, these mapping experiments establish the N-terminal Head domain as both necessary and sufficient for the direct physical interaction with eIF4H.

Having established the Head domain as the critical eIF4H-binding interface, we next sought to determine if this specific physical interaction is the causal driver of the downstream molecular and functional effects. To this end, we directly compared the functional capacity of wild-type KRT14 (KRT14-WT) against the interaction-deficient mutant (KRT14-ΔHead; hereafter KRT14-MUT). We first confirmed that the KRT14–eIF4H interaction is essential for modulating the translation machinery. While KRT14-WT enhanced the association between eIF4H and the core eIF4F complex, the KRT14-MUT failed to do so (Fig. 6E). Consistent with this, KRT14-WT, but not KRT14-MUT, drove the recruitment of ACOX2 mRNA to heavy polysomes (Fig. 6F) and promoted the assembly of the eIF4H-containing RNP complex on the ACOX2 transcript (Fig. 6G). Most critically, this molecular deficit translated directly to a loss of the pro-tumorigenic phenotype. In stark contrast to KRT14-WT, the KRT14-MUT was incapable of driving cell proliferation (Fig. S6A, B), inducing the high-lipid metabolic state (Fig. S6C–E), or conferring cisplatin resistance (Fig. 6H). In summary, these experiments demonstrate that the physical binding of eIF4H to the KRT14 Head domain is the indispensable, initiating event that unleashes the entire downstream cascade, culminating in therapeutic resistance.

### KRT14 overexpression promotes BLCA tumor growth and confers cisplatin resistance in vivo

**Fig. 7:**
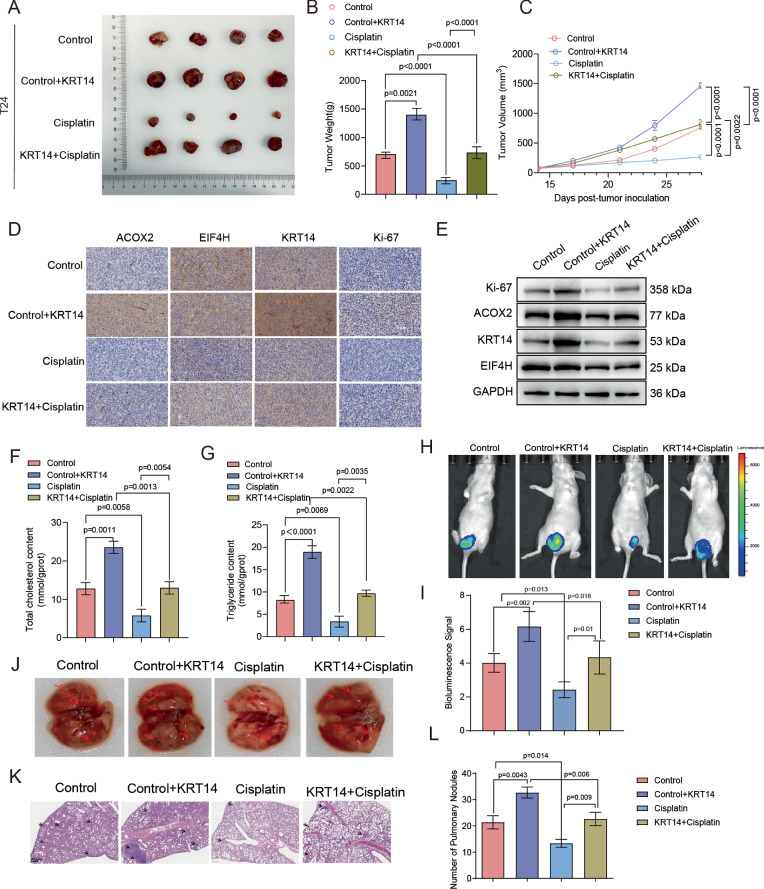
KRT14 OE promotes in vivo tumor growth and cisplatin resistance. **A**–**G** KRT14 overexpression promotes in vivo tumor growth and confers resistance to cisplatin in a T24 xenograft model. **A** Representative images of excised tumors, **B** final tumor weights, and **C** tumor growth curves consistently show that KRT14 overexpression accelerates tumor growth and mitigates the suppressive effects of cisplatin treatment. At the molecular level, **D** immunohistochemistry and **E** Western blot analyses confirm KRT14 overexpression and demonstrate a corresponding increase in the proliferation marker Ki67, as well as ACOX2 and EIF4H. Furthermore, KRT14 overexpression resulted in significantly elevated intratumoral levels of **F** total cholesterol and **G** triglycerides. (n = 4); **H**–**L** to validate these findings in a more physiologically relevant setting, an orthotopic bladder cancer model was established using the same cell groups. **H** Representative bioluminescence images and **I** quantification of total tumor burden demonstrate that KRT14 promotes tumor growth and confers resistance to cisplatin. **J** Representative gross images of lungs showing metastatic nodules (red arrows), **K** corresponding H&E staining of lung sections confirming metastatic lesions, and **L** quantification of pulmonary nodules further reveal that KRT14 potently drives metastatic dissemination and blunts the anti-metastatic effect of cisplatin. Data are presented as mean ± SD (n = 5); p-values are as indicated.

To translate our in vitro observations into a relevant physiological model, we next investigated the in vivo impact of KRT14 using subcutaneous xenografts. T24 BLCA cells stably overexpressing either KRT14 or a vector control were implanted into immunodeficient mice, which were then randomized into vehicle or cisplatin treatment groups. In the vehicle-treated cohort, KRT14 overexpression conferred a profound growth advantage. This was demonstrated by markedly larger excised tumors (Fig. 7A), a significant increase in final tumor weights (Fig. 7B), and accelerated tumor growth kinetics over the treatment course (Fig. 7C). We next assessed its role in therapeutic response. While cisplatin treatment suppressed tumor growth in both groups, the KRT14-overexpressing tumors exhibited a significantly diminished response, maintaining a much larger tumor burden compared to cisplatin-treated controls. This demonstrates that KRT14 overexpression confers substantial cisplatin resistance in vivo. IHC analysis confirmed robust KRT14 overexpression and, critically, revealed a coordinate upregulation of its downstream effectors, eIF4H and ACOX2 (Fig. 7D). This finding was quantitatively corroborated by Western blot analysis of tumor lysates (Fig. 7E). Consistent with their enhanced growth, KRT14-overexpressing tumors also showed a marked increase in the proliferation marker Ki67 (Fig. 7D). Furthermore, we confirmed that KRT14 drives metabolic reprogramming in vivo. Biochemical analysis revealed that KRT14-overexpressing tumors exhibited a profound accumulation of both total cholesterol and triglycerides, a high-lipid phenotype that was maintained irrespective of cisplatin treatment (Fig. 7F, G).

To corroborate these findings in a more clinically relevant microenvironment and to assess the impact of KRT14 on metastatic potential, we established a parallel orthotopic bladder cancer model using the same experimental groups. Real-time monitoring via bioluminescence imaging confirmed that KRT14 overexpression significantly increased the overall tumor burden and conferred robust resistance to cisplatin treatment (Fig. 7H, I). Critically, this orthotopic model enabled the evaluation of distant metastasis, a key hallmark of advanced disease. At the experimental endpoint, we observed a dramatic increase in the number of pulmonary metastatic nodules in mice bearing KRT14-overexpressing tumors (Fig. 7J, L). This was further confirmed by histological analysis of lung sections (Fig. 7K). Importantly, while cisplatin treatment effectively reduced metastasis in control tumors, its anti-metastatic effect was significantly blunted in the KRT14-overexpressing group (Fig. 7L). Taken together, these complementary in vivo models provide compelling evidence that KRT14 is a key driver of multiple facets of bladder cancer malignancy, including local growth, metabolic reprogramming, chemoresistance, and distant metastasis.

### The KRT14-ACOX2 axis is a clinical driver of cisplatin resistance in bladder cancer

**Fig. 8:**
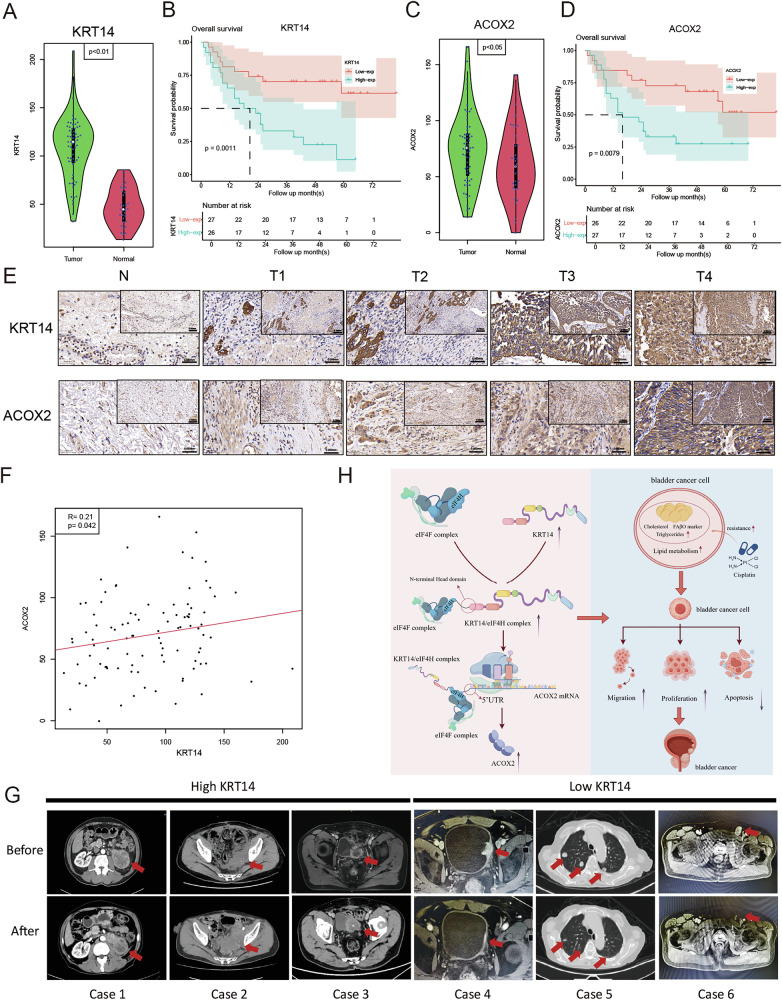
Clinical relevance and prognostic value of KRT14 and ACOX2 in BLCA. Clinical relevance and prognostic value of KRT14 and ACOX2 in bladder cancer. Violin plots show significantly higher expression of **A** KRT14 (p < 0.01) and **C** ACOX2 (p < 0.05) in tumor tissues compared to normal tissues. Kaplan–Meier survival analysis for the patient cohort. High expression of **B** KRT14 (p = 0.0011) and **D** ACOX2 (p = 0.0079) was significantly associated with poorer overall survival. **E** Representative immunohistochemistry images illustrate stronger staining for KRT14 and ACOX2 in various tumor samples (T1–T4) compared to normal tissue (N) at both 200× and 400× magnification. **F** A scatter plot reveals a significant positive correlation between the expression of KRT14 and ACOX2 in tumors (R = 0.21, p = 0.042). **G** Representative CT scans from six patients undergoing cisplatin-based chemotherapy, stratified by KRT14 expression status. Cases 1–3, with high KRT14 expression, show progressive or stable disease after treatment. In contrast, Cases 4–6, with low KRT14 expression, show a favorable partial response with significant tumor shrinkage. Red arrows indicate representative tumor lesions. **H** Schematic model illustrating the proposed mechanism by which KRT14 interacts with eIF4H to selectively promote ACOX2 translation, leading to altered lipid metabolism and ultimately conferring cisplatin resistance and promoting malignant phenotypes in bladder cancer.

To translate our mechanistic findings into a clinical context, we first evaluated the prognostic value of the KRT14-ACOX2 axis in the large, independent TCGA-BLCA dataset. Kaplan–Meier analysis revealed that high expression of either KRT14 or ACOX2 was a strong predictor of poor patient outcomes, correlating with significantly shorter overall survival, and their combined high expression identified the patient subgroup with the most dismal prognosis (Fig. S7A, B, D). While KRT14 and ACOX2 mRNA levels showed only a weak positive correlation (Fig. S7C), consistent with our finding of post-transcriptional regulation, these data from a large cohort strongly support the clinical importance of this axis. Having established the prognostic significance of the KRT14-ACOX2 axis, we next sought to validate these findings at the protein level in our own cohort of BLCA patient tissues via IHC. The baseline clinicopathological characteristics of this cohort are detailed in Table 1. We first observed a stark and significant upregulation of KRT14 protein in tumor tissues compared to paired adjacent normal tissues (Fig. 8A). Remarkably, the expression pattern of its downstream effector, ACOX2, perfectly recapitulated that of KRT14, also showing significant and specific elevation in tumors (Fig. 8C). Representative IHC images visually confirmed the marked and heterogeneous upregulation of both proteins in tumor specimens (Fig. 8E). Furthermore, expression levels of KRT14 and ACOX2 were strongly and positively correlated with each other across the patient cohort, providing clinical evidence for the co-regulation of this axis (Fig. 8F). Crucially, the clinical significance of this axis was underscored by its prognostic power. Kaplan–Meier analysis revealed that high expression of either KRT14 (Fig. 8B) or ACOX2 (Fig. 8D) was a strong predictor of poor patient outcomes, correlating with significantly shorter overall survival. These data establish the KRT14-ACOX2 axis as a robust marker of adverse prognosis in bladder cancer.

Lastly, to directly link our findings to therapeutic outcomes, we present radiological evidence from six patients that illustrates the potential of KRT14 as a predictive biomarker for cisplatin response (Fig. 8G). A clear correlation was observed: the three patients with KRT14-high tumors (Cases 1–3) all exhibited resistance to gemcitabine-cisplatin chemotherapy, with imaging showing no significant regression or progression of their lesions. In stark contrast, all three patients with KRT14-low tumors (Cases 4–6) demonstrated a favorable response, with follow-up CT scans revealing a marked reduction in their target lesions. Collectively, these results showed that high KRT14 expression correlates with a poor response to cisplatin-based therapy. They provide powerful clinical validation for our mechanistic model (summarized in Fig. 8H), positioning the KRT14-ACOX2 axis as a critical driver of therapeutic failure and a promising target for overcoming chemoresistance in bladder cancer.

## Discussion

Cisplatin-based chemotherapy remains a cornerstone of treatment for bladder cancer (BLCA); however, both intrinsic and acquired resistance substantially limit its efficacy, leading to tumor recurrence and poor patient outcomes [9, 33]. Elucidating the molecular mechanisms underlying this resistance is essential for the development of novel therapeutic strategies. In this study, we identify KRT14 as a pivotal driver of cisplatin resistance and malignant progression in BLCA. We systematically demonstrate that KRT14 is markedly upregulated in BLCA tissues, particularly in cisplatin-resistant patient samples and derived cell lines. Importantly, through complementary gain- and loss-of-function approaches conducted both in vitro and in vivo, we establish a causal link between elevated KRT14 expression and key hallmarks of cancer, including enhanced proliferation, suppressed apoptosis, increased motility, and, most notably, pronounced cisplatin resistance. These findings position KRT14 not merely as a marker, but as a functional contributor to BLCA aggressiveness and therapeutic failure.

Our investigation significantly advances the understanding of KRT14’s function beyond its established roles in maintaining epithelial integrity [34] and its association with specific molecular subtypes, such as basal-like or squamous features, in various cancers, including BLCA [35, 36]. Whereas previous studies have primarily linked high KRT14 levels to cellular differentiation status [37] or associated its expression with poor prognosis in BLCA and other malignancies [38], our work is distinguished by providing direct experimental evidence for a causative role of KRT14 specifically in mediating chemoresistance. We unequivocally demonstrate that manipulating KRT14 levels directly alters cellular sensitivity to cisplatin—a finding robustly confirmed in preclinical xenograft models, where KRT14 knockdown enhanced cisplatin efficacy and overexpression conferred resistance. This mechanistic insight extends beyond previous correlative observations and highlights KRT14 as a potential therapeutic vulnerability. This finding stands in contrast to many established resistance mechanisms in BLCA, which primarily focus on drug efflux pumps (e.g., ABC transporters) [39, 40], DNA repair pathways [41], or signaling cascades like EGFR/MAPK [42], thereby presenting a novel axis for therapeutic intervention. Furthermore, our study expands the known repertoire of KRT14-driven phenotypes in BLCA to include direct modulation of cell survival and migration, suggesting a broader and more pleiotropic role in tumor progression than previously appreciated.

A key contribution of our study is the comprehensive delineation of the downstream signaling pathways mediating the effects of KRT14. We have uncovered a novel mechanistic link between KRT14 and the regulation of cellular lipid metabolism. Specifically, cisplatin-resistant cells exhibit elevated lipid content, and KRT14 expression is required to sustain this altered metabolic phenotype. While the observed reductions in total cholesterol and triglycerides following KRT14 knockdown are statistically significant, their magnitude may appear modest. However, we argue their biological significance is substantial. Crucially, our functional rescue experiments confirmed that these metabolic alterations are functionally relevant, as partially restoring lipid metabolism was sufficient to significantly reverse the cisplatin sensitization induced by KRT14 knockdown. Furthermore, these changes in specific lipid pools should be viewed as indicators of a broader metabolic reprogramming orchestrated by KRT14, a conclusion supported by our GSEA results showing suppression of the entire fatty acid biosynthesis pathway and the dramatic downregulation of the fatty acid β-oxidation marker FAβO. Therefore, KRT14 appears to act as a crucial upstream regulator that fine-tunes a pro-survival lipidomic profile, where even modest but consistent alterations are sufficient to confer a significant survival advantage under therapeutic stress.

While metabolic reprogramming—particularly involving lipids, such as fatty acid synthesis and cholesterol metabolism—is increasingly recognized as a critical driver of cancer progression and therapeutic resistance across various tumor types [43, 44], our study uniquely identifies KRT14, a cytoskeletal intermediate filament protein, as an upstream regulator orchestrating these specific lipidomic changes (elevated cholesterol and triglycerides) in the context of BLCA cisplatin resistance. The upstream nature of KRT14 in this axis is further underscored by our findings that direct activation of downstream lipid metabolism does not, in turn, affect KRT14 expression, ruling out a reciprocal feedback loop (Fig. S8). This establishes a previously uncharacterized mechanistic connection between intermediate filament dynamics and metabolic adaptation during therapeutic stress.

Mechanistically, we identified ACOX2—an enzyme best known for its role in peroxisomal fatty acid β-oxidation and bile acid metabolism—as a critical downstream effector of KRT14 [45]. ACOX2 expression closely paralleled that of KRT14, and, importantly, rescue experiments demonstrated that ACOX2 is indispensable for KRT14-driven cellular phenotypes. Although ACOX2 dysregulation has been implicated in various other cancers, often exhibiting context-dependent functions [46], its specific regulation by KRT14 and its direct role in mediating KRT14-driven malignancy and cisplatin resistance in BLCA represent a novel discovery. The KRT14-ACOX2 axis thus defines a distinct regulatory pathway, separate from other known modulators of ACOX2 expression or activity in diverse cellular contexts.

Perhaps most strikingly, our study elucidates the molecular mechanism by which KRT14 regulates ACOX2 expression through post-transcriptional control. We demonstrate that KRT14 directly interacts with the eukaryotic translation initiation factor eIF4H, an association specifically mediated by the Head domain of KRT14. This interaction enhances the normal association of eIF4H with the eIF4F complex, thereby facilitating enhanced translational efficiency of ACOX2 mRNA. To ascertain the specificity of this mechanism, we examined whether KRT14 might function as a general enhancer of protein synthesis. However, a SUnSET assay revealed that KRT14 overexpression did not alter the rate of global translation (Fig. S9A), strongly indicating that its effect is selective. To further explore whether ACOX2 is a unique target or part of a broader regulatory program, we performed quantitative proteomic profiling. This unbiased screen revealed that KRT14 knockdown led to a significant downregulation of a cohort of proteins that were strongly enriched in lipid and cholesterol metabolic pathways (Fig. S9B, C). This finding provides powerful, independent evidence that KRT14 orchestrates a broader translational program to remodel cellular lipid metabolism. Although ACOX2 itself was not identified as a significant hit in this high-throughput screen, likely due to technical limitations inherent to discovery proteomics, its pivotal role is unequivocally supported by our targeted validation experiments (e.g., Western blot, functional rescue). Therefore, we propose a model where ACOX2 is a key, functionally essential effector within a wider network of lipid-related proteins translationally controlled by the KRT14–eIF4H complex. This discovery uncovers a previously unrecognized function of KRT14, acting as a specific regulator of translation initiation for a defined downstream target. Whereas keratin interactions with signaling adapters or scaffolds to facilitate signaling cascades are well established [47], the direct binding and functional modulation of a core translation initiation factor such as eIF4H by a keratin protein constitutes a major conceptual advance. Traditionally, translational control in cancer is centered on the eIF4E-mTOR axis or involves RNA-binding proteins that regulate mRNA stability or subcellular localization [48, 49]. Our findings reveal a distinct mechanism whereby a structural protein directly influences the translation machinery for specific mRNA targets (such as ACOX2), suggesting a previously unappreciated layer of gene expression regulation orchestrated by intermediate filaments in response to cellular stress and contributing to chemoresistance.

Despite the comprehensive nature of our findings, several limitations warrant discussion. While our use of orthotopic tumor models more closely mimics the relevant tumor microenvironment compared to subcutaneous xenografts, and our proteomic analyses have broadened our understanding of KRT14’s impact, the primary limitations of our study relate to the clinical data.

Most importantly, our clinical findings, while providing valuable preliminary support for our mechanistic discoveries, are limited by the modest sample size of our retrospective cohort. Although our expanded analysis of treatment response via CT imaging revealed a strong and consistent clinical trend, the strength of our prognostic conclusions is restricted. However, we substantially mitigated this limitation by validating our key clinical findings in the large-scale TCGA-BLCA public dataset. This independent analysis confirmed that high expression of the KRT14-ACOX2 axis is a robust predictor of poor overall survival, thus reinforcing the broad clinical relevance of our conclusions. Due to the limited sample size, we were unable to perform multivariate analyses to adjust for potential confounding factors and definitively establish KRT14 as an independent prognostic marker. Therefore, our clinical conclusions should be considered hypothesis-generating. Larger, prospective studies are essential to definitively validate the prognostic and predictive value of the KRT14-ACOX2 axis in a multicenter setting. Additionally, while our study focused on KRT14’s downstream effects, the upstream signals responsible for its upregulation in resistant BLCA remain an important area for future exploration.

Building upon our findings, several promising avenues for therapeutic exploration emerge. The identification of the KRT14–eIF4H interaction as a central driver of cisplatin resistance highlights this interface as a novel and highly attractive target. Although targeting protein–protein interactions (PPIs), particularly those involving cytoskeletal proteins lacking enzymatic pockets, has historically been challenging, the high specificity of this interaction offers a unique opportunity. Rational strategies may include high-throughput small-molecule screening guided by future structural characterization of the KRT14 head domain bound to eIF4H, as well as the design of peptide-based inhibitors such as stapled peptides to competitively disrupt the interface. More advanced approaches—such as PROteolysis-TArgeting Chimeras (PROTACs) that selectively degrade KRT14—could provide an additional layer of therapeutic innovation. These modalities, if successful, would not only serve as valuable preclinical tools but also hold translational potential to re-sensitize bladder tumors to cisplatin. In parallel, investigating the upstream signaling pathways that drive KRT14 overexpression and validating KRT14 and ACOX2 as predictive biomarkers will be critical steps to enable patient stratification and accelerate clinical translation.

In conclusion, our study identifies KRT14 as a key functional driver of cisplatin resistance and multiple malignant phenotypes in bladder cancer. We unveil a novel regulatory axis in which KRT14, through direct interaction with eIF4H mediated by its Head domain, promotes the translation of ACOX2. This, in turn, leads to altered lipid metabolism, ultimately fostering cellular proliferation, survival, and chemoresistance. Our findings not only provide important new insights into the molecular underpinnings of BLCA progression and therapeutic failure, but also highlight the KRT14–eIF4H–ACOX2 pathway as a potential source of novel prognostic biomarkers and therapeutic targets for overcoming cisplatin resistance in patients with BLCA.

## Supplementary information

Figure S1

Figure S2

Figure S3

Figure S4

Figure S5

Figure S6

Figure S7

Figure S8

Figure S9

Supplementary Figure Legend

Unprocessed Western Blot Image

Supplementary Table S1

## Data Availability

The datasets generated and analyzed during the current study are not publicly available due to data ownership and confidentiality considerations, but are available from the corresponding author on reasonable request.
