# Supplementary figures and images for: Cytoskeletal protein KRT14 governs cisplatin resistance by modulating eIF4H-dependent ACOX2 translation and lipid metabolism in bladder cancer
Source: Cell Death Dis. 2025 Dec 24;17(1):134. doi: 10.1038/s41419-025-08369-3 (PMC12847778; doi:10.1038/s41419-025-08369-3)

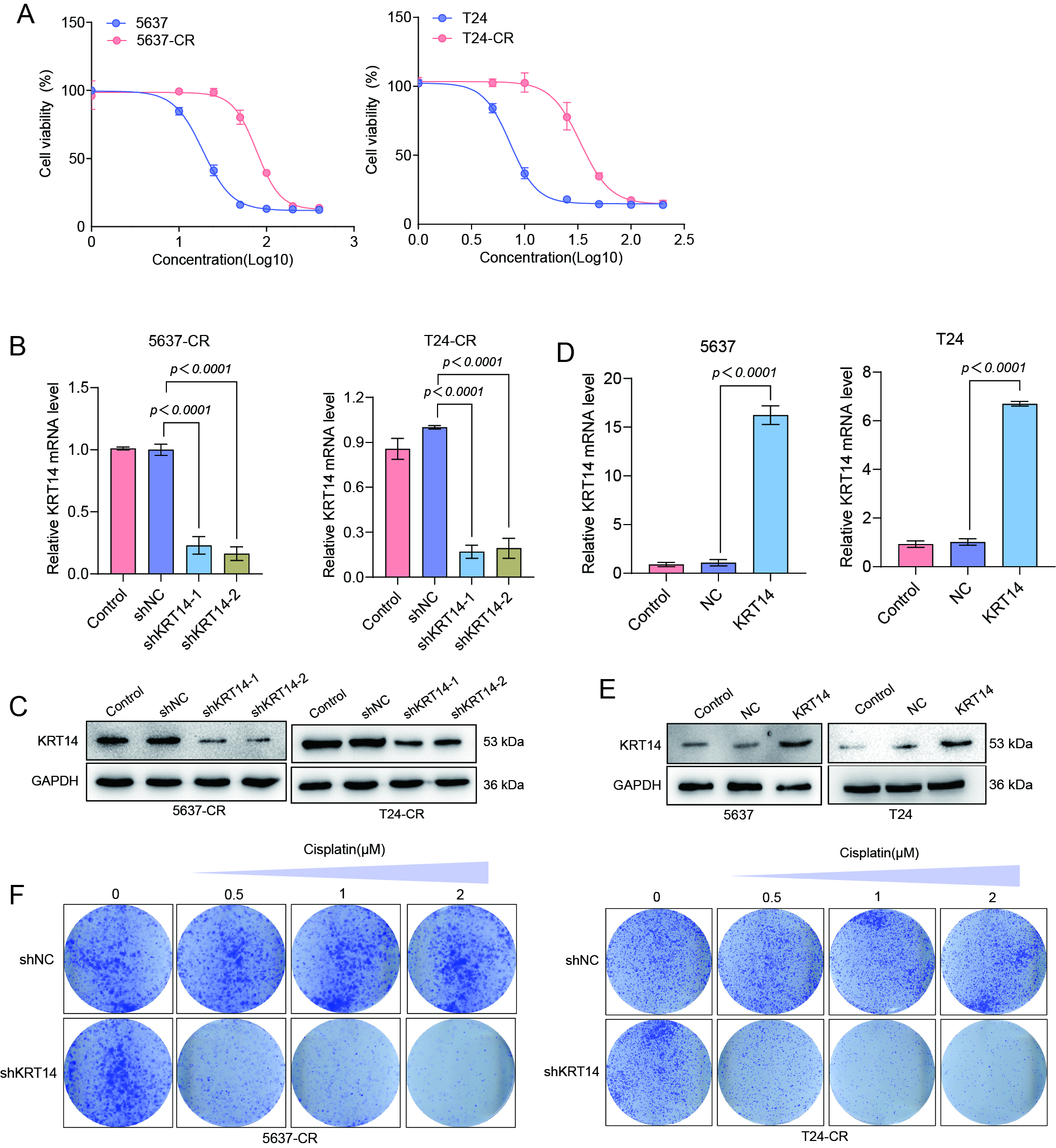

Supplement: Supplementary file 1 — Figure S1 [file 41419_2025_8369_MOESM1_ESM.tif]

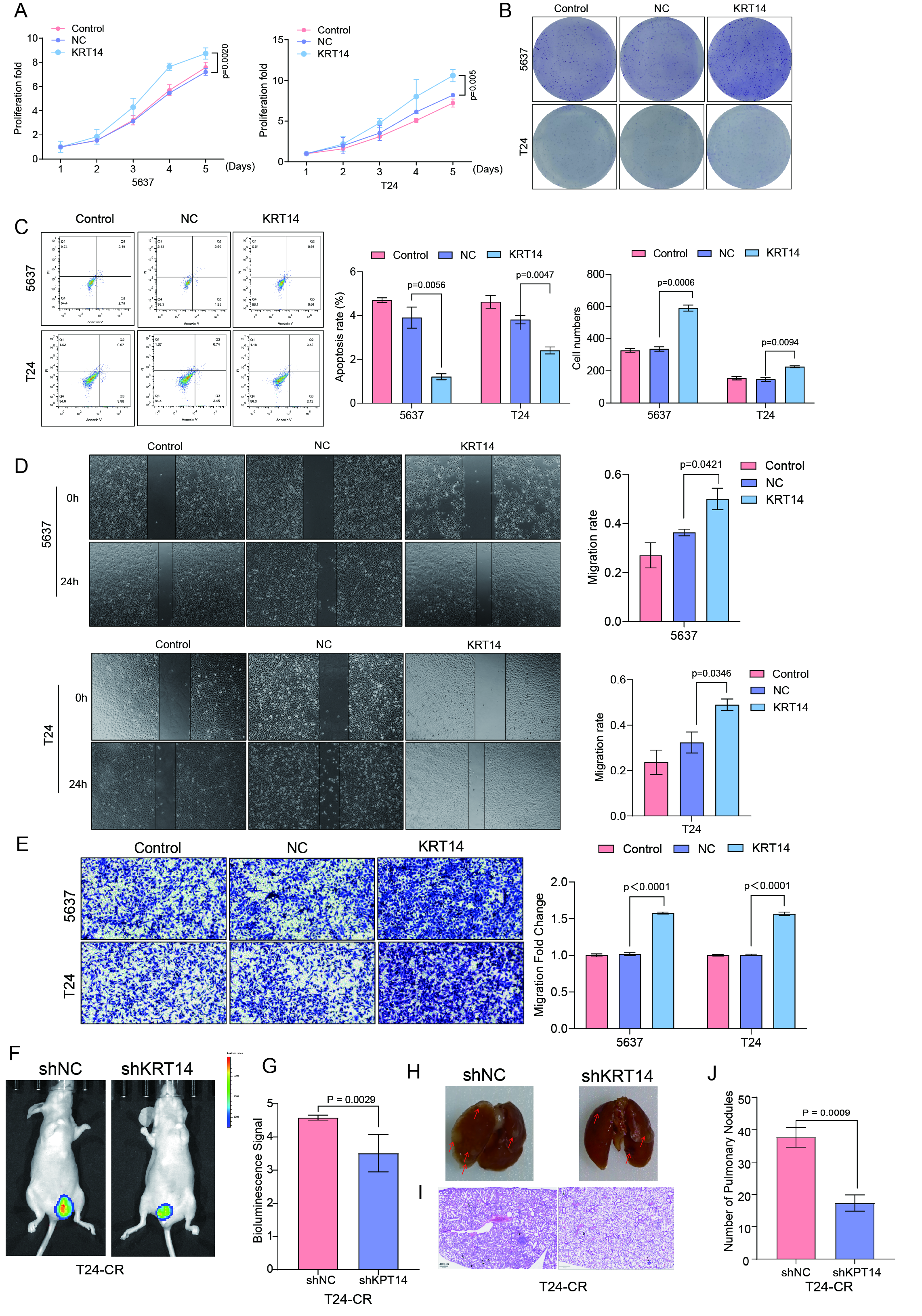

Supplement: Supplementary file 2 — Figure S2 [file 41419_2025_8369_MOESM2_ESM.tif]

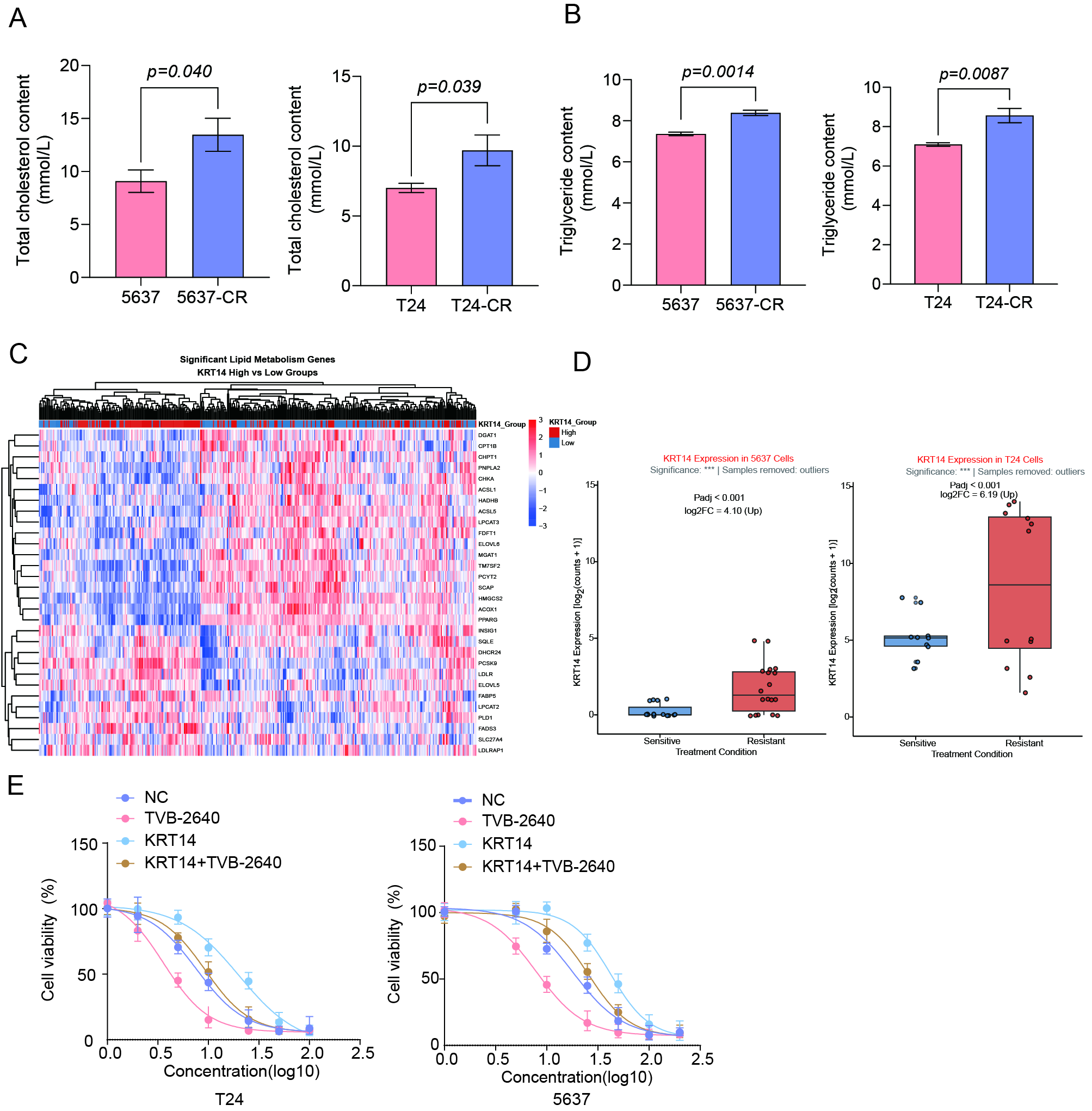

Supplement: Supplementary file 3 — Figure S3 [file 41419_2025_8369_MOESM3_ESM.tif]

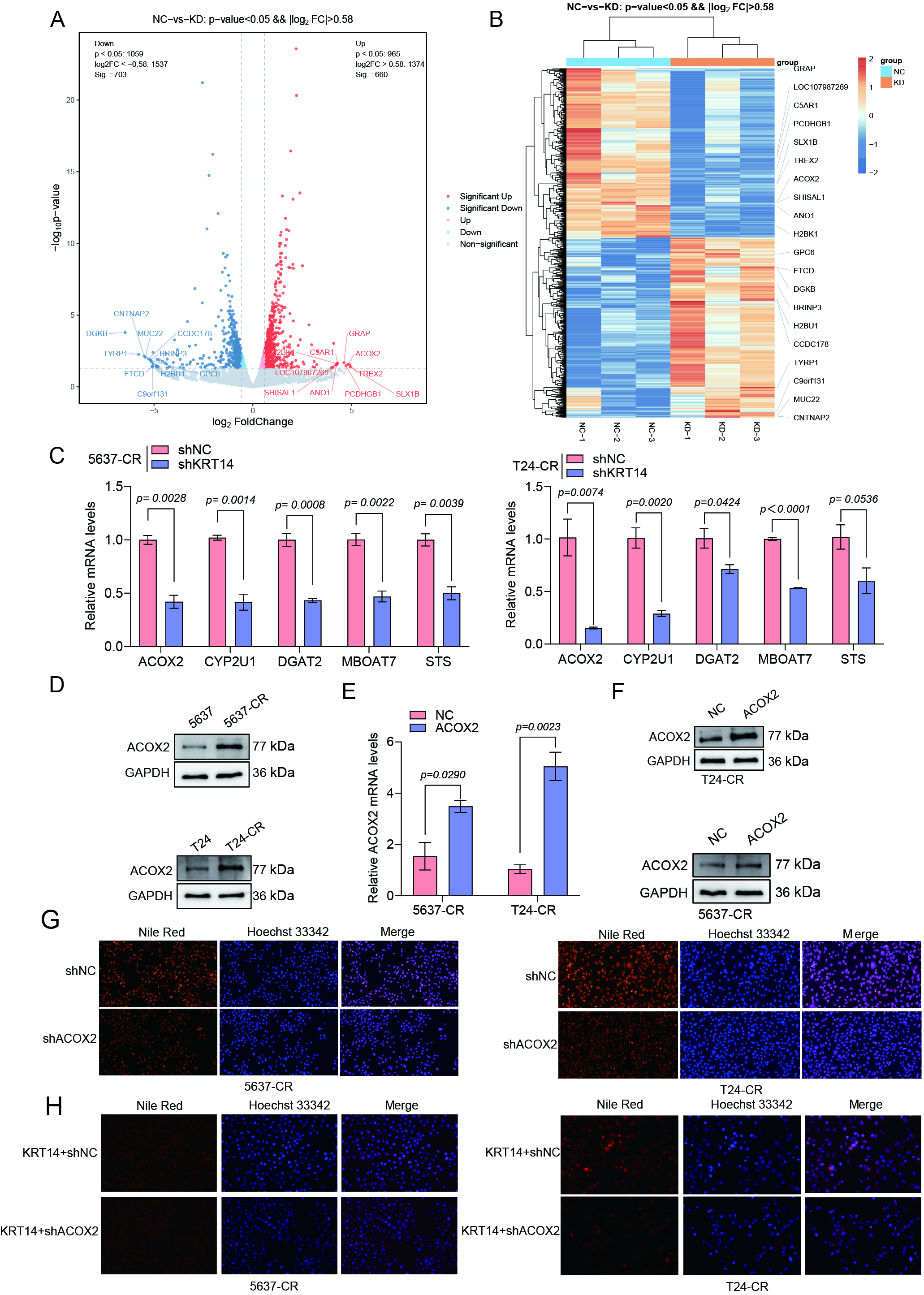

Supplement: Supplementary file 4 — Figure S4 [file 41419_2025_8369_MOESM4_ESM.tif]

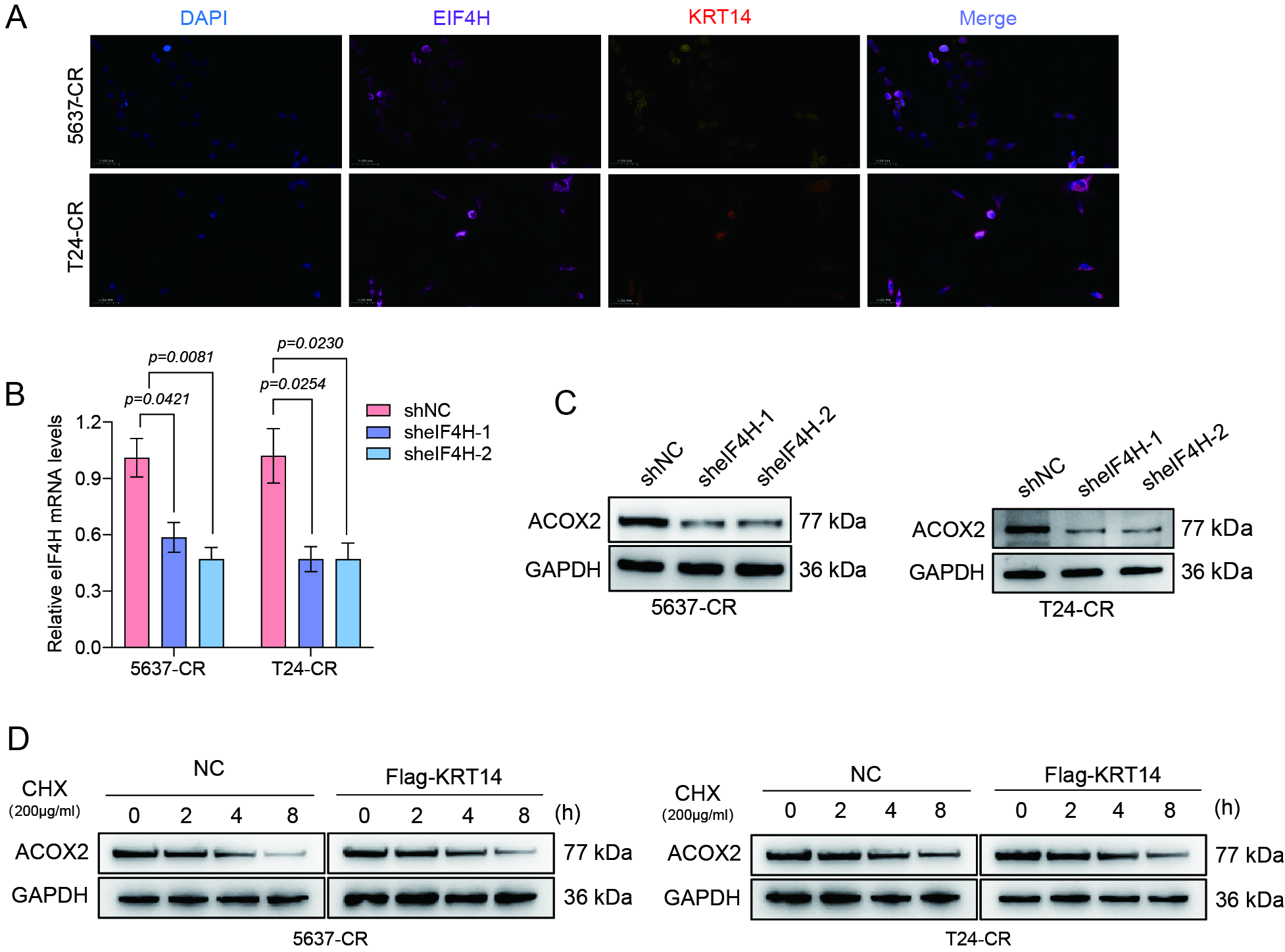

Supplement: Supplementary file 5 — Figure S5 [file 41419_2025_8369_MOESM5_ESM.tif]

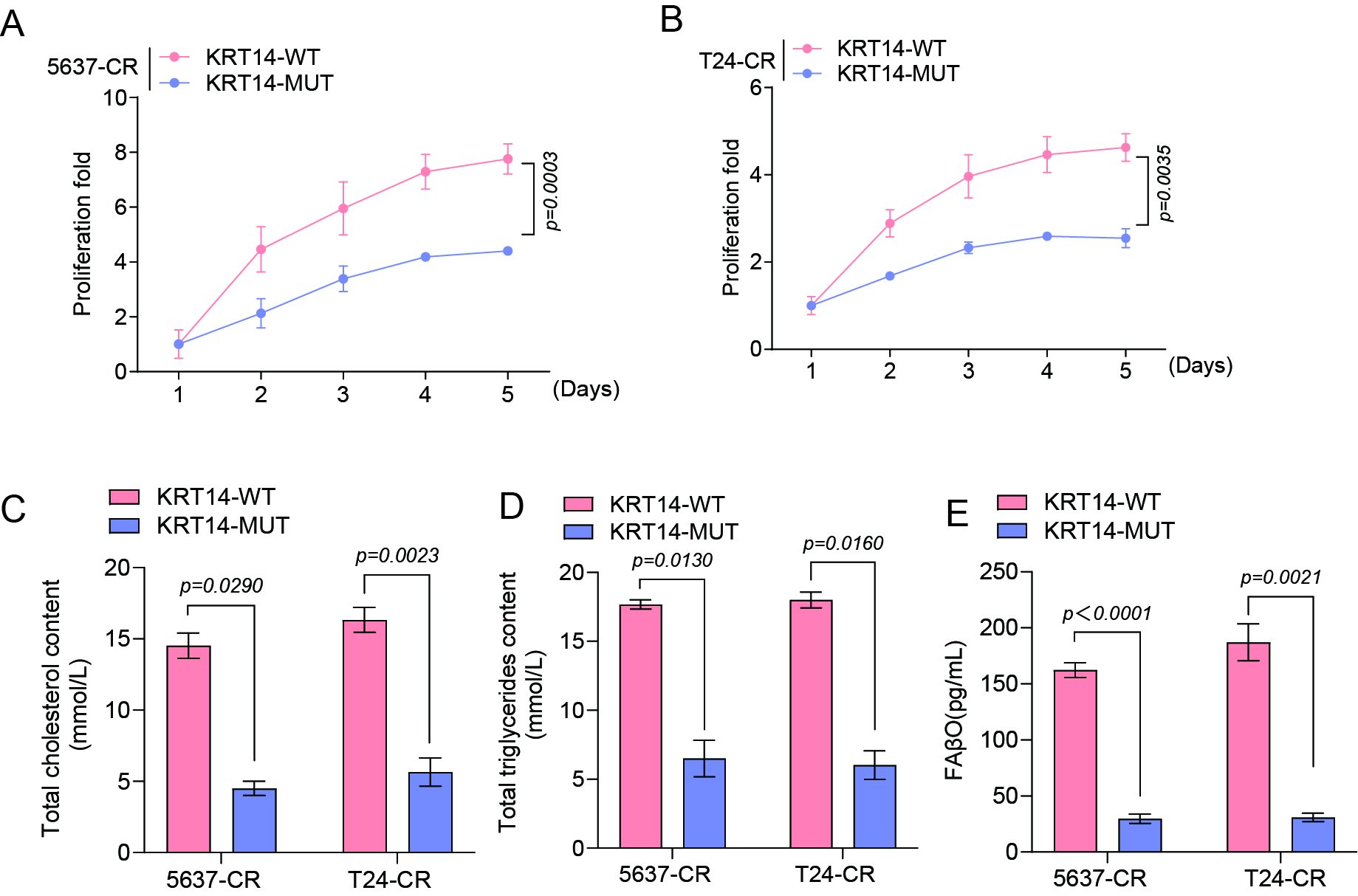

Supplement: Supplementary file 6 — Figure S6 [file 41419_2025_8369_MOESM6_ESM.tif]

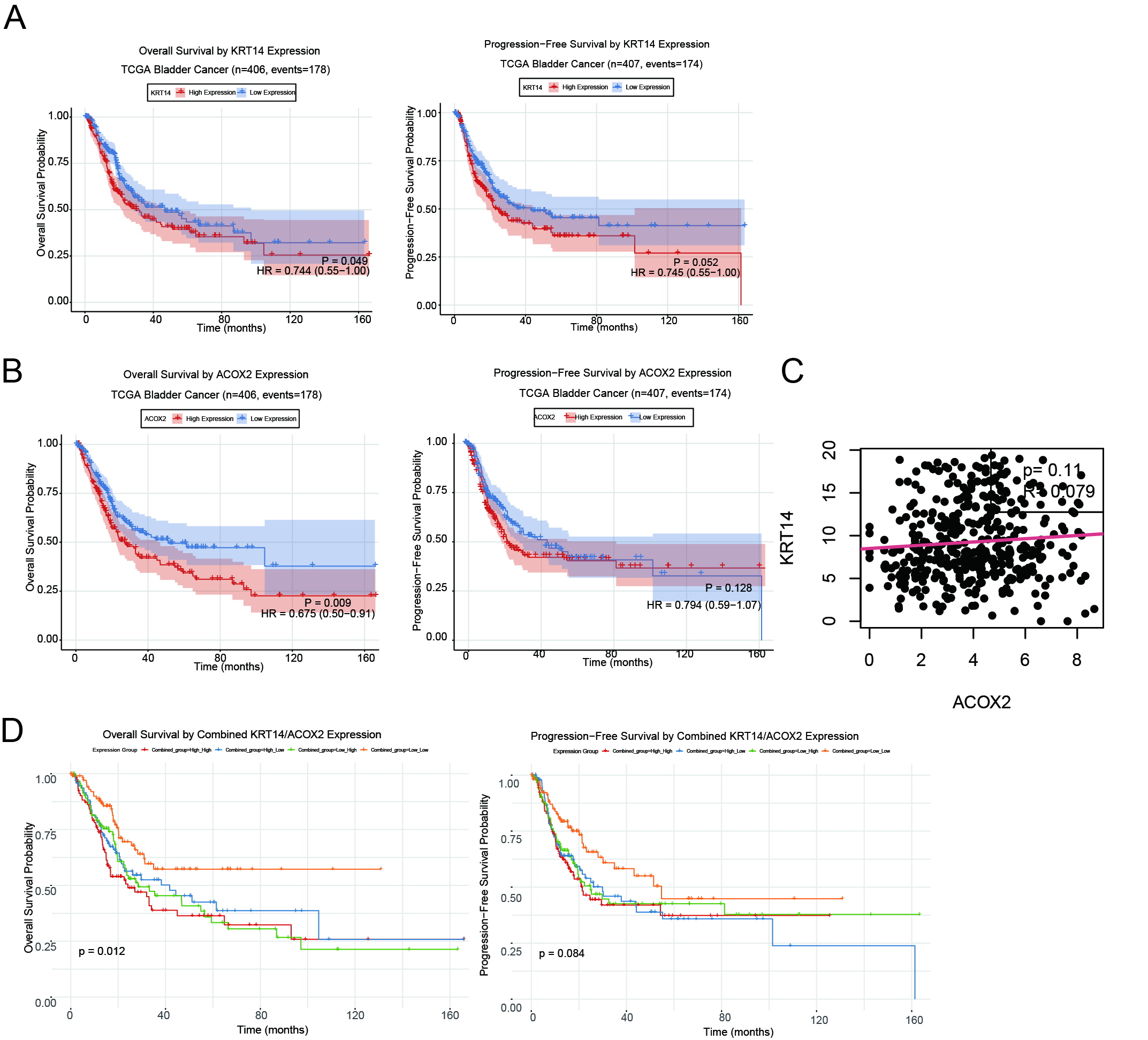

Supplement: Supplementary file 7 — Figure S7 [file 41419_2025_8369_MOESM7_ESM.tif]

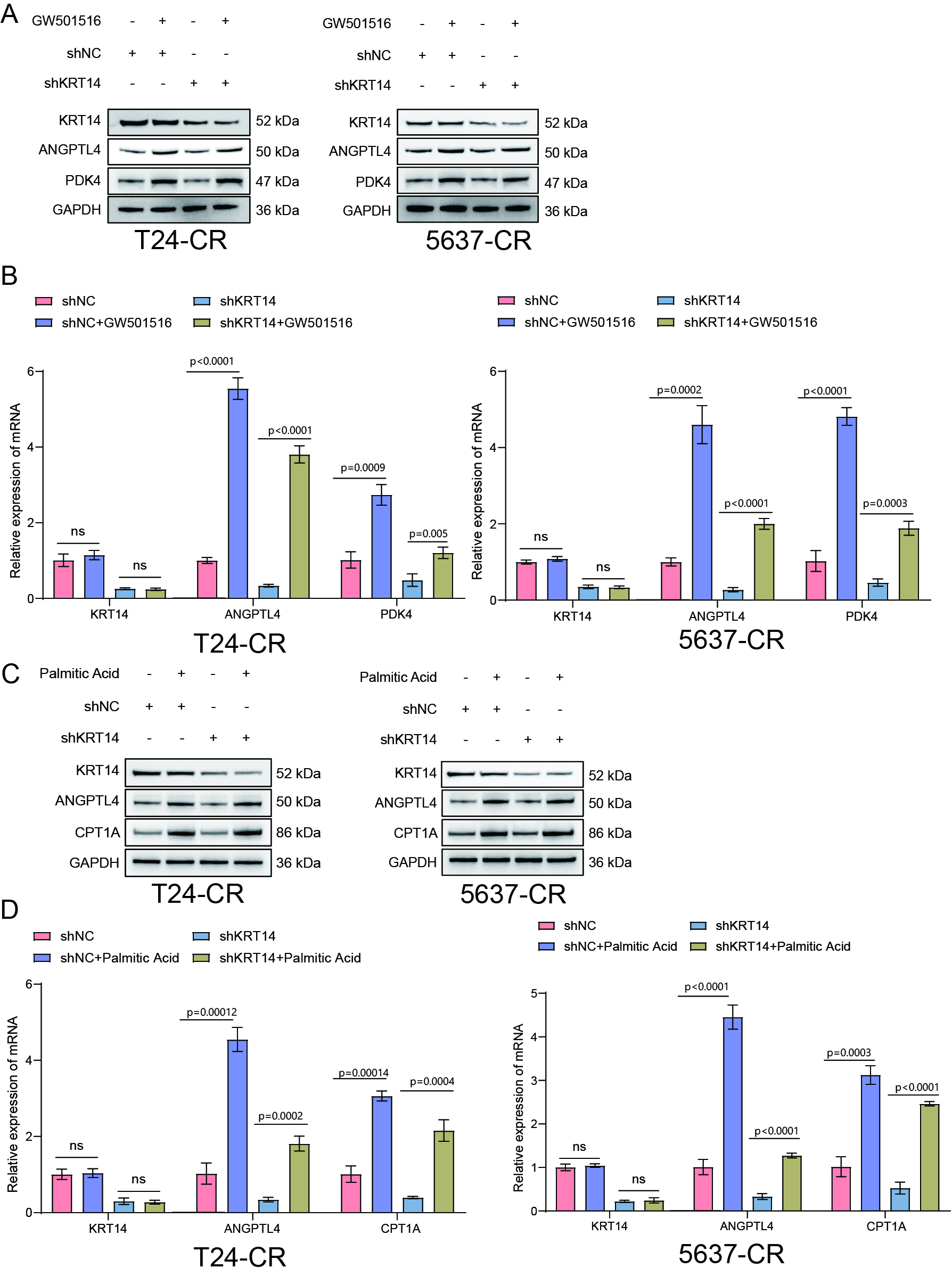

Supplement: Supplementary file 8 — Figure S8 [file 41419_2025_8369_MOESM8_ESM.tif]

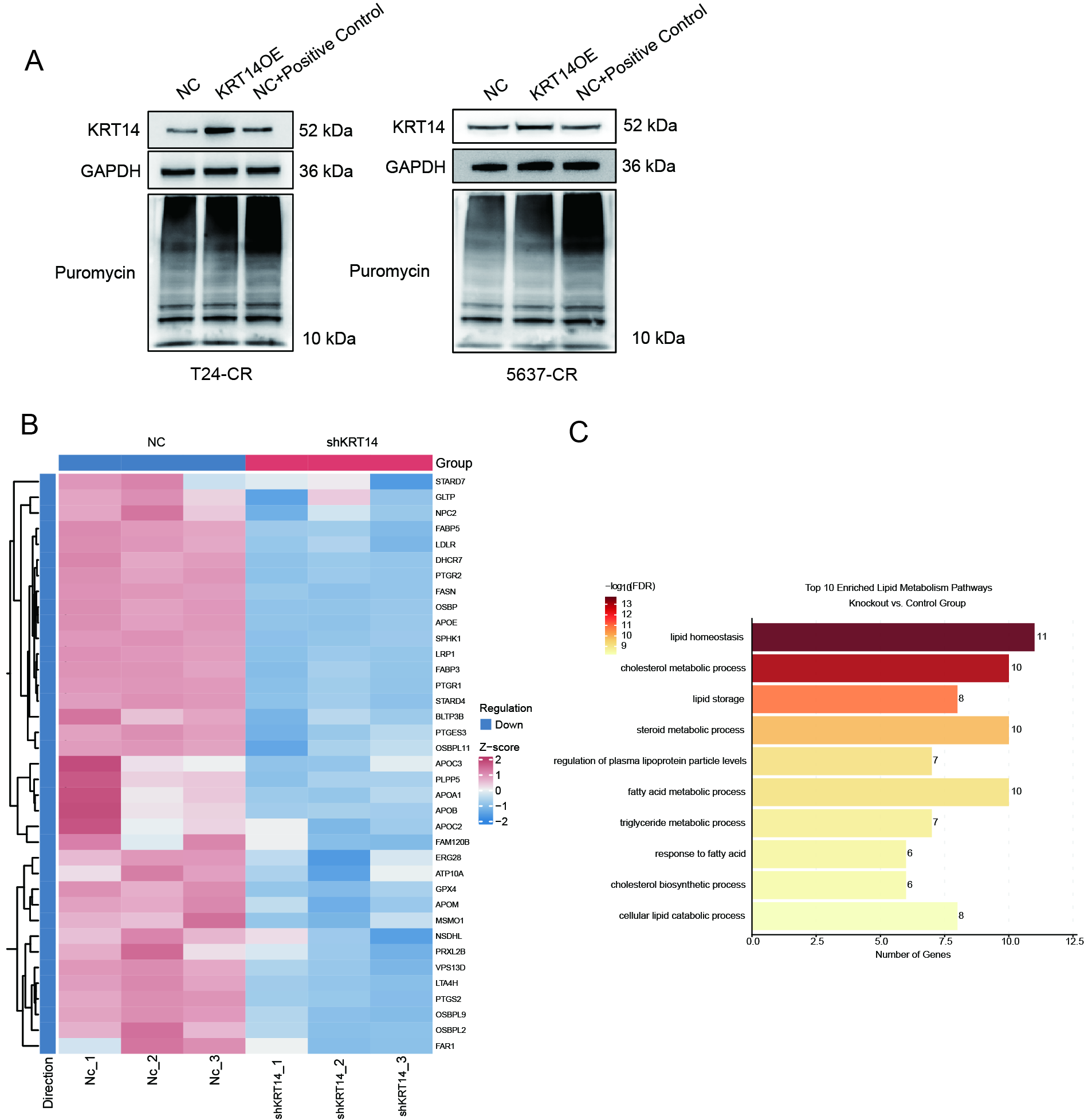

Supplement: Supplementary file 9 — Figure S9 [file 41419_2025_8369_MOESM9_ESM.tif]
