# Supplementary material for: Cytoskeletal protein KRT14 governs cisplatin resistance by modulating eIF4H-dependent ACOX2 translation and lipid metabolism in bladder cancer
Source: Cell Death Dis. 2025 Dec 24;17(1):134. doi: 10.1038/s41419-025-08369-3 (PMC12847778; doi:10.1038/s41419-025-08369-3)
Supplement: Supplementary file 10 — Supplementary Figure Legend [file 41419_2025_8369_MOESM10_ESM.docx]

**Supplementary Figure legend**

**Figure S1. Validation of KRT14 knockdown/overexpression and cell line resistance.** (A) Cisplatin viability curves (CCK-8, 48h) confirming resistance phenotype of 5637-CR/T24-CR vs. parental cells. (B, C) Validation of KRT14 knockdown by RT-qPCR (B) and WB (C) using shRNAs in resistant cells. (D, E) Validation of KRT14 overexpression by RT-qPCR (D) and WB (E) in parental cells. (F) Representative colony formation assays showing shKRT14 sensitizes resistant cells to cisplatin.

**Figure S2. KRT14 overexpression promotes malignant phenotypes *in vitro* and KRT14 knockdown suppresses tumor progression and metastasis *in vivo*.**

1. E) Overexpression of KRT14 in parental, cisplatin-sensitive 5637 and T24 cells is sufficient to drive multiple malignant phenotypes. Cells expressing a control vector, an empty vector (NC), or a KRT14 overexpression vector were assessed for: (A) Proliferation rates by CCK-8 assay over 5 days. (B) Long-term colony formation ability after 14 days. (C) Susceptibility to apoptosis, assessed by Annexin V/PI flow cytometry. Representative plots and quantification are shown. (D) Cell migration ability in a wound healing assay over 24 hours. Representative images (Scale bar=200µm) and quantification of the migration rate are provided. (E) Invasive potential in a Transwell migration assy. (F-J) Silencing KRT14 in cisplatin-resistant T24-CR cells suppresses tumor growth and metastasis in an orthotopic bladder cancer model. (F) Representative bioluminescence images of mice bearing orthotopic tumors expressing either shNC or shKRT14. (G) Quantification of the total bioluminescence signal, indicating a reduction in tumor burden upon KRT14 knockdown. (H) Representative gross images of lungs showing a marked decrease in metastatic nodules in the shKRT14 group. (I) Corresponding hematoxylin and eosin (H&E) staining of lung sections, confirming the reduction in metastatic lesions. (J) Quantification of the number of pulmonary nodules on the lung surface.Resentative images (Magnification=100×) and quantification of migrated cells are shown.Data are presented as mean ± SD, n=3. P-values are as indicated.

**Figure S3. KRT14 is associated with a lipogenic phenotype, and its chemoresistance-conferring function is dependent on lipid metabolism.**

(A, B) Cisplatin-resistant (CR) bladder cancer cells display a distinct high-lipid metabolic state, characterized by significantly elevated intracellular levels of (A) total cholesterol and (B) triglycerides compared to their parental counterparts. (C) This clinical relevance is underscored by public data analysis from the TCGA-BLCA cohort, where high KRT14 expression is associated with a distinct lipid metabolism gene signature, as shown by the heatmap. (D) Furthermore, KRT14 upregulation is confirmed as a consistent feature of chemoresistance in independent cisplatin-resistant 5637 and T24 cell line models from publicly available datasets. (E) To functionally validate this dependency, we performed a pharmacological rescue experiment. While KRT14 overexpression conferred significant cisplatin resistance in both T24 and 5637 cells, co-treatment with the Fatty Acid Synthase (FASN) inhibitor TVB-2640 substantially reversed this phenotype, re-sensitizing the cells to cisplatin. Data in A, B, and E are presented as mean ± SD n=3; p-values are as indicated.

**Figure S4. ACOX2 expression, function, and related transcriptomics.**

Volcano plot of DEGs from RNA-seq (shKRT14 vs. shNC). (B) Heatmap of significant DEGs from (A). (C) RT-qPCR validation of selected downregulated genes in resistant cells ± shKRT14. (D) WB shows higher baseline ACOX2 protein in resistant vs. parental cells. (E, F) RT-qPCR (E) and WB (F) confirm ACOX2 OE efficiency. (G) Nile Red staining shows shACOX2 reduces lipid droplets in resistant cells. Scale bar=50µm. (H) Nile Red staining shows shACOX2 reduces lipid droplets even with KRT14 OE. Scale bar=50µm with p-values indicated for each comparison.

**Figure S5. Controls and validation for KRT14-eIF4H interaction studies.**

(A) IF shows cytoplasmic co-localization of endogenous KRT14 (Red) and eIF4H (Magenta) in resistant cells. Scale bar=50µm. (B, C) Validation of eIF4H knockdown by RT-qPCR (B) and WB showing reduced ACOX2 protein (C). (D) CHX chase assay (WB, 0-8h) shows KRT14 OE does not alter ACOX2 protein stability with p-values indicated for each comparison.

**Figure S6:** **A KRT14 mutation attenuates proliferation and alters lipid metabolism in cisplatin-resistant bladder cancer cells.** This figure compares the functional consequences of expressing wild-type KRT14 (KRT14-WT) versus a mutant KRT14 (KRT14-MUT) in cisplatin-resistant cell lines. (A, B) Proliferation assays show that KRT14-WT expression leads to significantly higher cell proliferation rates compared to KRT14-MUT in both (A) 5637-CR and (B) T24-CR cells over five days. Furthermore, biochemical analysis reveals that KRT14-WT cells maintain significantly higher intracellular levels of (C, D) total cholesterol and (E) β-oxidation marker (labeled FAβO) than KRT14-MUT cells in both cell lines. Data are shown as mean ± SD, with p-values indicated for each comparison.

**Figure S7. Bioinformatic analysis of the TCGA-BLCA cohort validates the clinical relevance and prognostic value of the KRT14-ACOX2 axis.**

Kaplan-Meier survival analyses from the TCGA Bladder Cancer cohort (n≈406) show that high expression of (A) KRT14 (p=0.048) or (B) ACOX2 (p=0.009) individually correlates with significantly poorer Overall Survival (OS). (C) KRT14 and ACOX2 mRNA levels show a weak, non-significant positive correlation, consistent with the post-transcriptional nature of their regulatory relationship. (D) Critically, a combined analysis reveals that patients with concurrently high expression of both KRT14 and ACOX2 have the most unfavorable OS (p=0.012), underscoring the synergistic prognostic impact of the entire axis. P-values were determined by the log-rank test.

**Figure S8. KRT14 expression is not regulated by downstream lipid metabolism activation, confirming its upstream role.**

Cisplatin-resistant T24-CR and 5637-CR cells, stably expressing either a non-targeting control shRNA (shNC) or an shRNA against KRT14 (shKRT14), were treated as indicated. (A, B) Cells were treated with the PPARδ agonist GW501516 (10 µM) for 48 hours. KRT14, ANGPTL4, and PDK4 levels were assessed by (A) Western blot and (B) RT-qPCR. (C, D) Cells were treated with palmitic acid (100 µM) for 48 hours. KRT14, ANGPTL4, and CPT1A levels were assessed by (C) Western blot, and mRNA levels of KRT14, ANGPTL4, and PDK4 were assessed by (D) RT-qPCR. Across both cell lines and with both treatments, KRT14 expression at both the protein and mRNA level remained unchanged, ruling out the existence of a positive feedback loop. In contrast, the robust induction of downstream metabolic genes (ANGPTL4, CPT1A) served as a positive control for treatment efficacy. Data in B and D are presented as mean ± SD from three independent experiments. ns, not significant; with p-values indicated for each comparison.

**Figure S9. KRT14 selectively regulates a lipid metabolism proteome without altering global protein synthesis.**

1. A SUnSET assay demonstrates that KRT14 overexpression in T24-CR and 5637-CR cells does not alter the rate of global protein synthesis, as indicated by unchanged levels of puromycin incorporation into nascent proteins. (B) To identify specific targets, quantitative proteomic profiling was performed on T24-CR cells with KRT14 knockdown (shKRT14) versus control (NC). The heatmap displays a distinct cohort of proteins significantly downregulated upon KRT14 silencing. (C) Gene Ontology (GO) enrichment analysis of these downregulated proteins reveals a strong and significant enrichment for biological processes related to lipid homeostasis, cholesterol metabolism, and lipid storage, highlighting the specific role of KRT14 in orchestrating a lipid-centric proteome.
