# Supplementary material for: Cytoskeletal protein KRT14 governs cisplatin resistance by modulating eIF4H-dependent ACOX2 translation and lipid metabolism in bladder cancer
Source: Cell Death Dis. 2025 Dec 24;17(1):134. doi: 10.1038/s41419-025-08369-3 (PMC12847778; doi:10.1038/s41419-025-08369-3)
Supplement: Supplementary file 11 — Unprocessed Western Blot Image [file 41419_2025_8369_MOESM11_ESM.pdf]

Figure1E

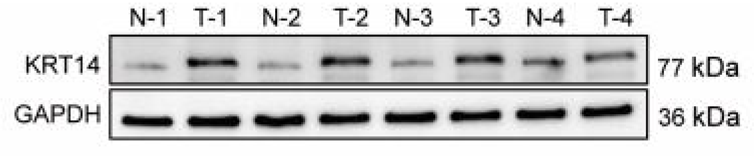

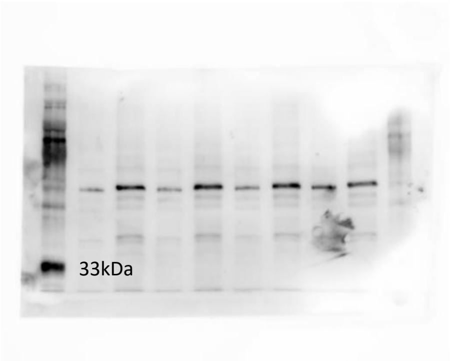

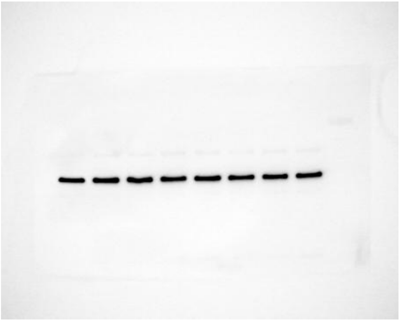

KRT14

GAPDH

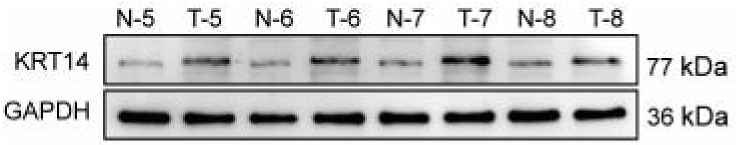

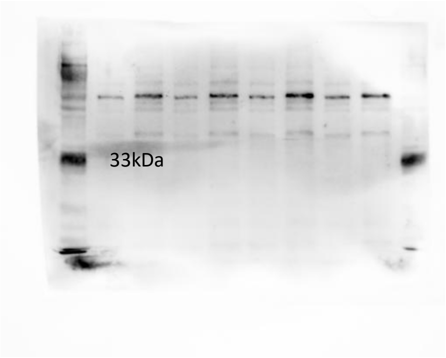

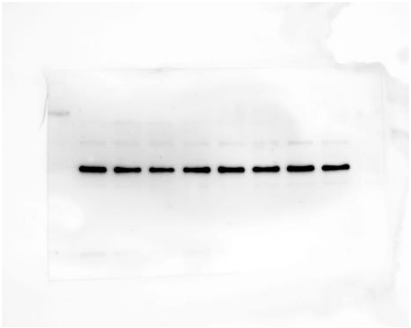

KRT14

GAPDH

Figure 1F

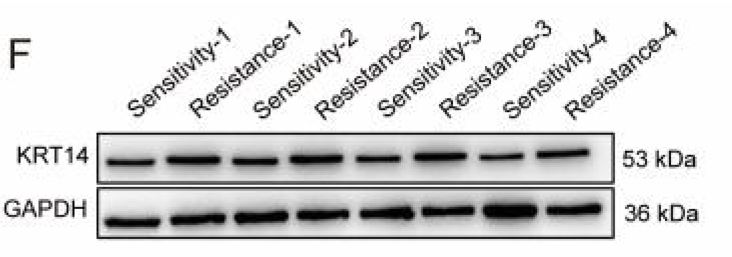

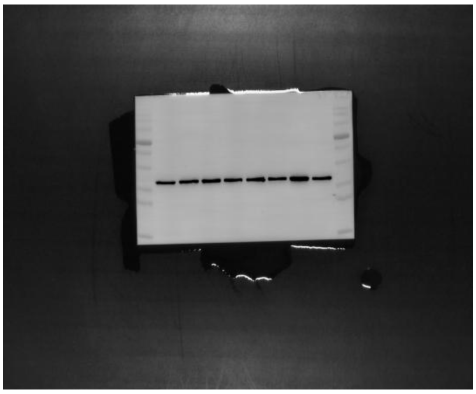

KRT14

GAPDH

Figure 1G

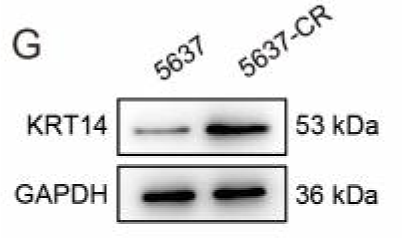

KRT14

GAPDH

Figure 1H

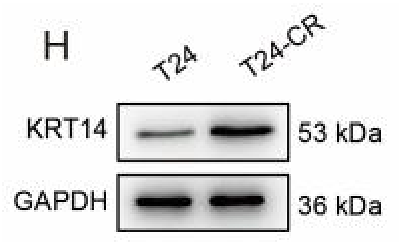

KRT14

GAPDH

Figure 4B

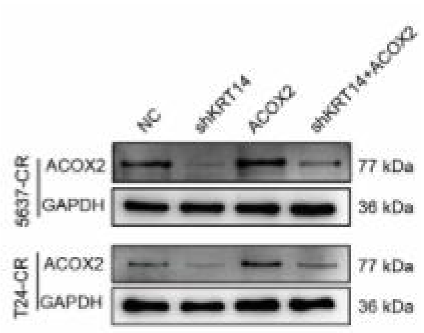

5637-CR

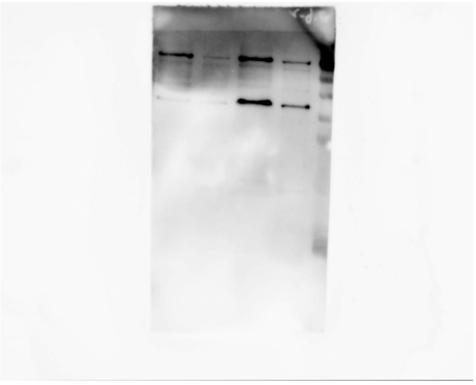

ACOX2

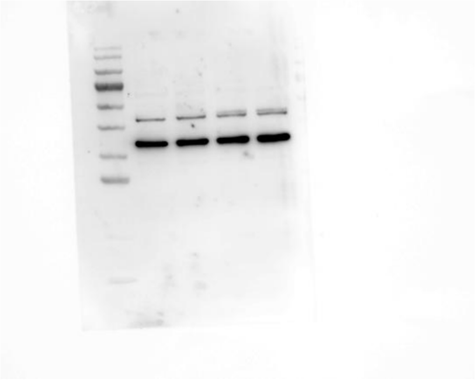

GAPDH

T24-CR

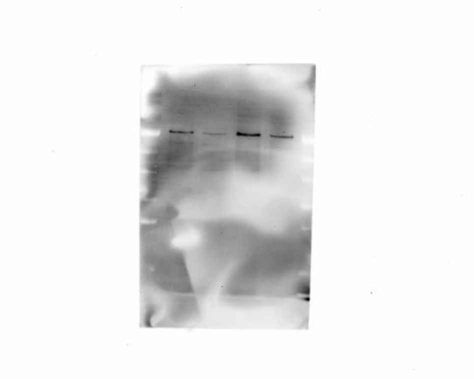

ACOX2

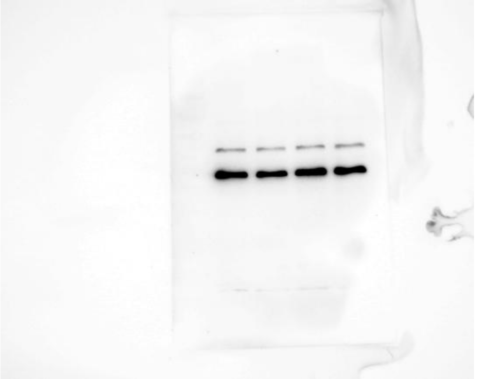

GAPDH

Figure5B

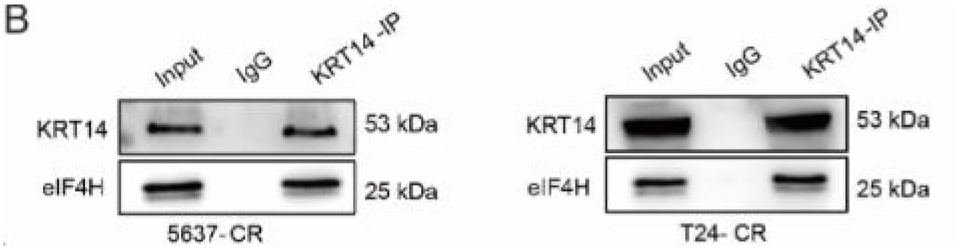

5637-CR

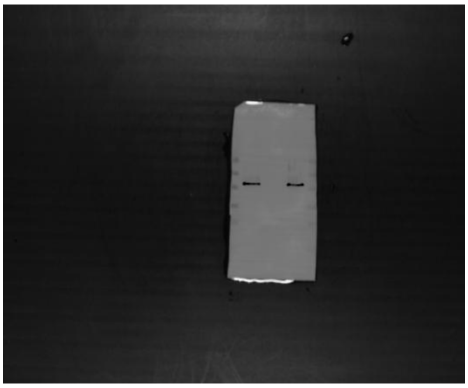

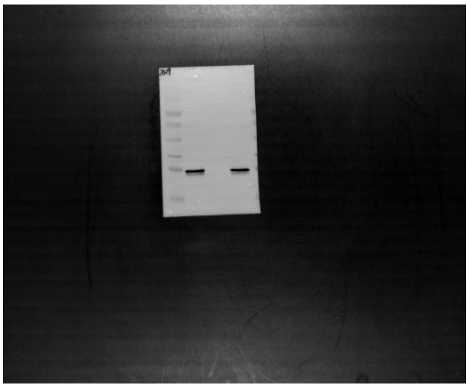

KRT14

ELF4H

T24-CR

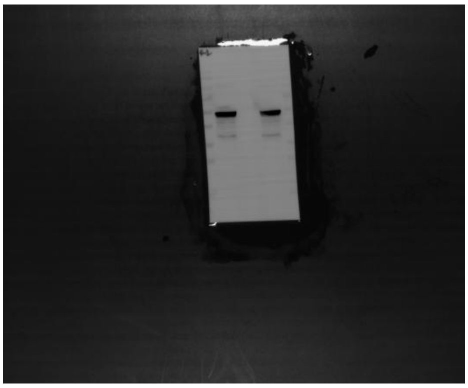

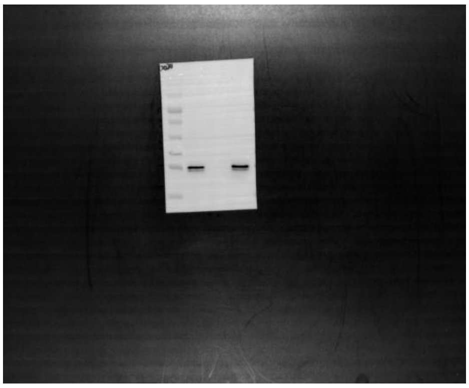

KRT14

ELF4H

Figure5C

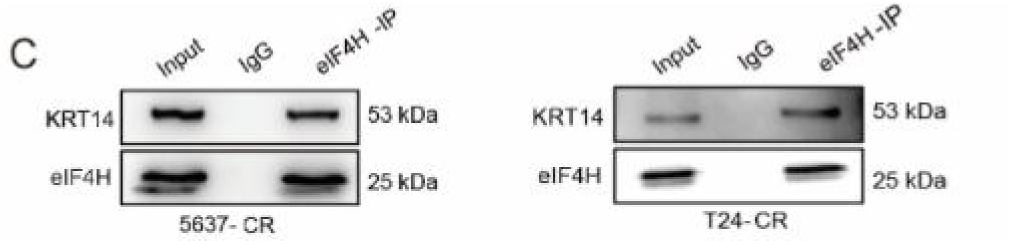

5637-CR

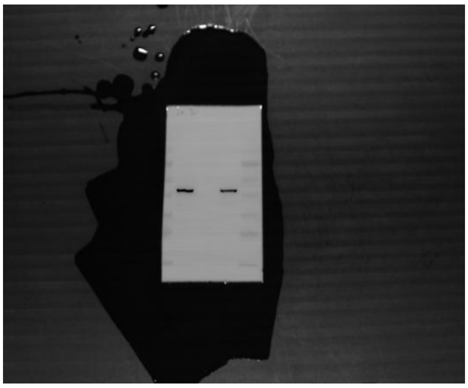

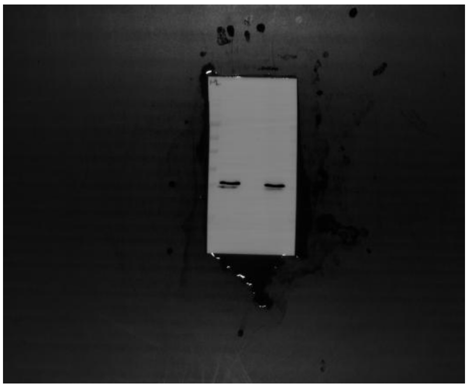

KRT14

ELF4H

T24-CR

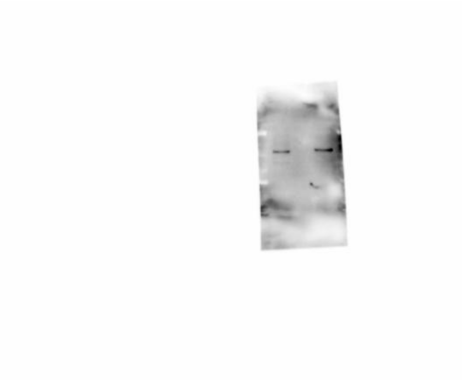

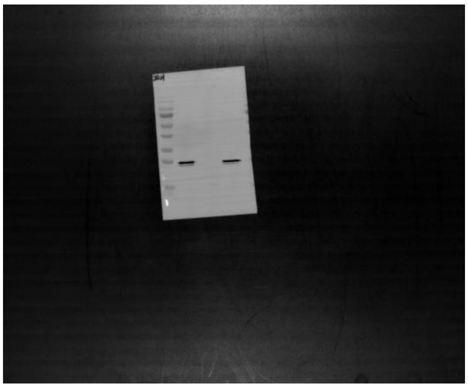

KRT14

ELF4H

Figure5D

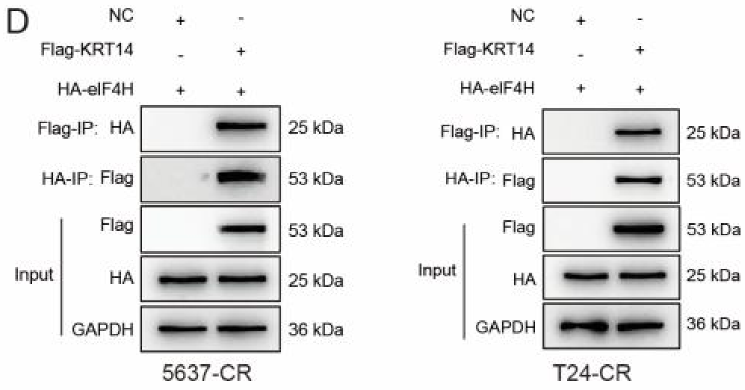

5637-CR

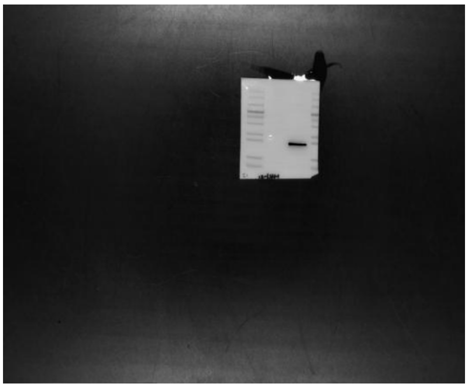

HA-IP

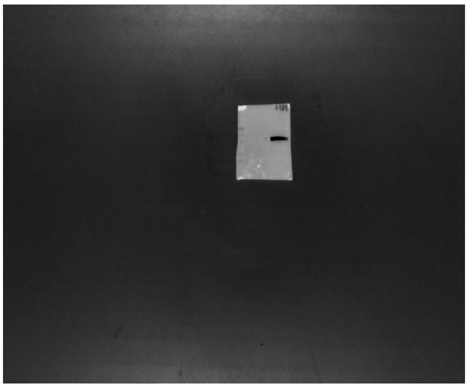

Flag-IP

Input：

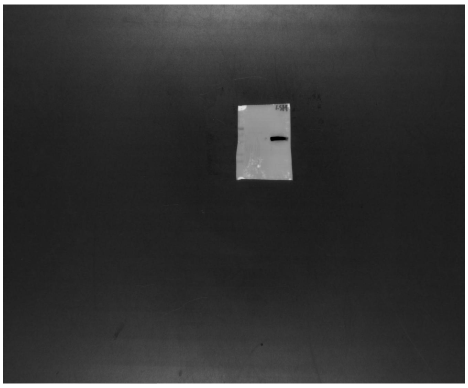

Flag-tag

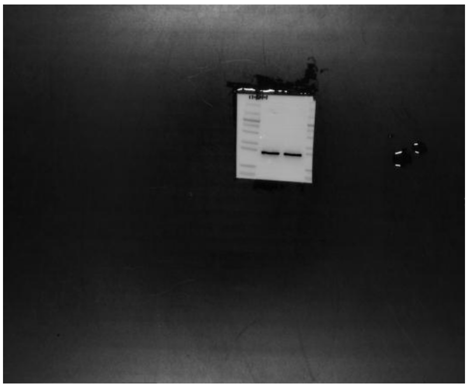

HA-tag

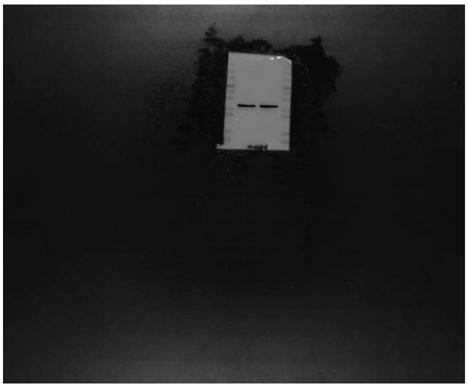

GAPDH

T24-CR

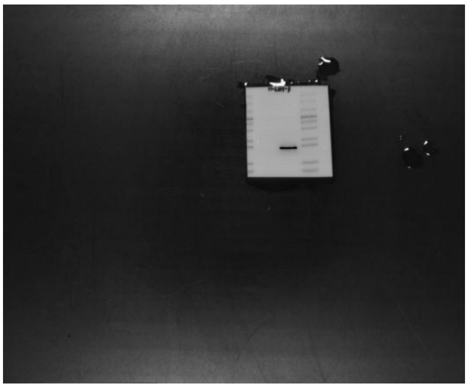

HA-IP

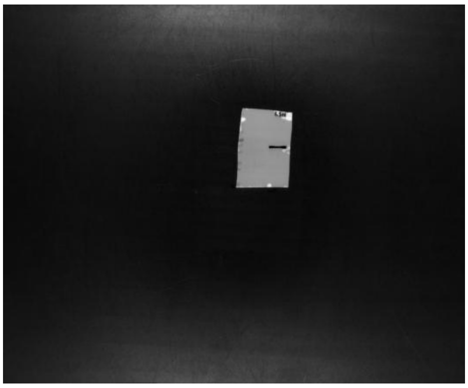

Flag-IP

Input：

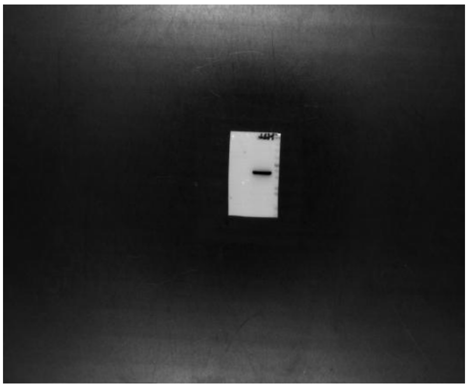

Flag-tag

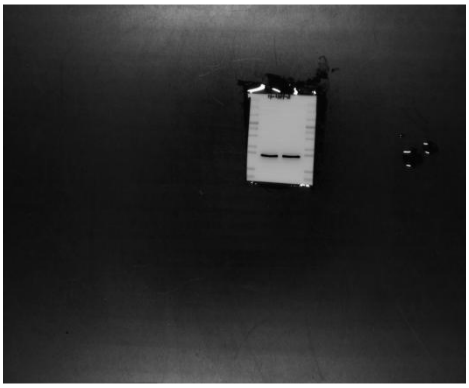

HA-tag

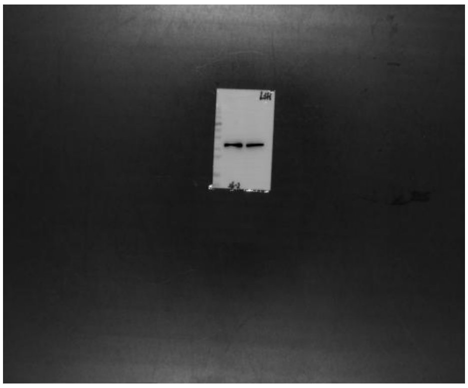

GAPDH

Figure5E

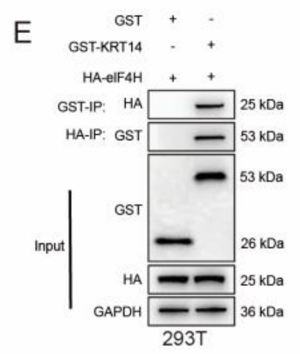

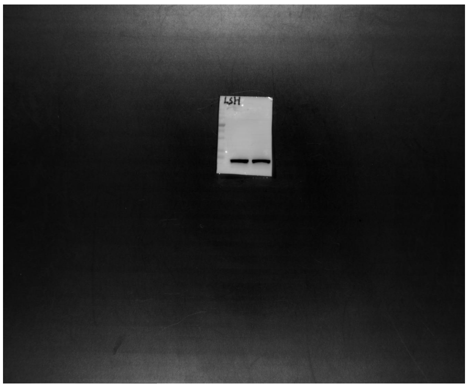

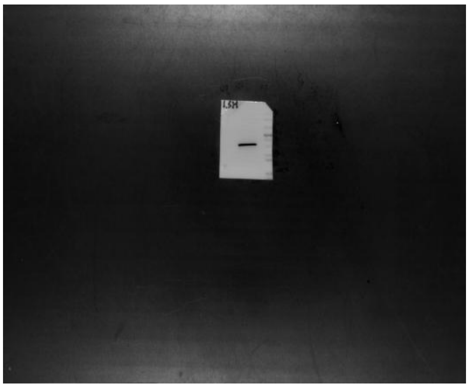

HA

GST-tag

Input

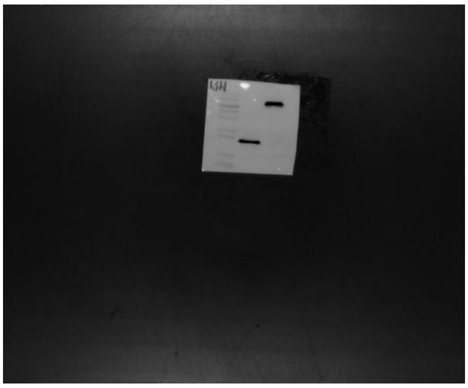

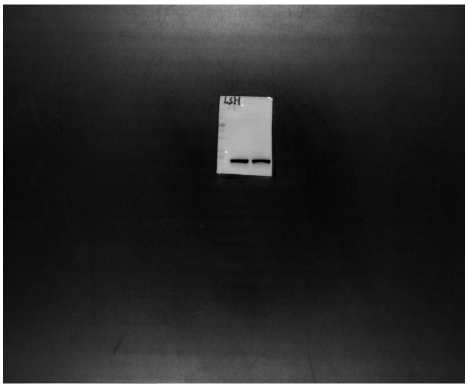

GST

HA

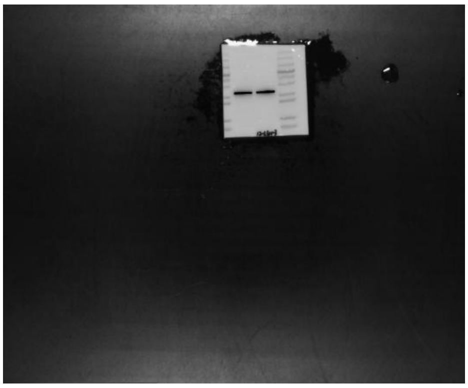

GAPDH

Figure5F

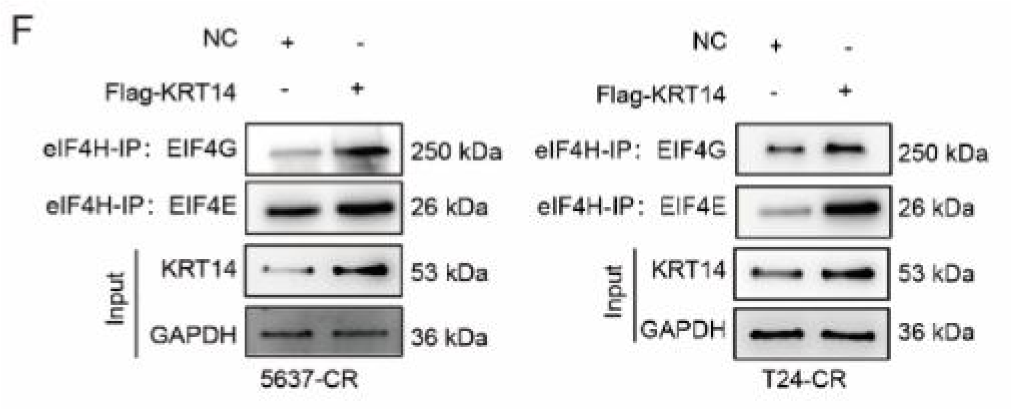

5637-CR

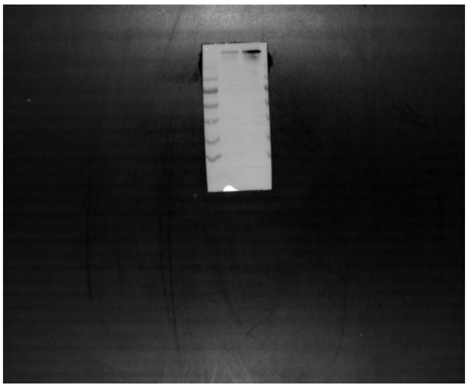

EIF4G

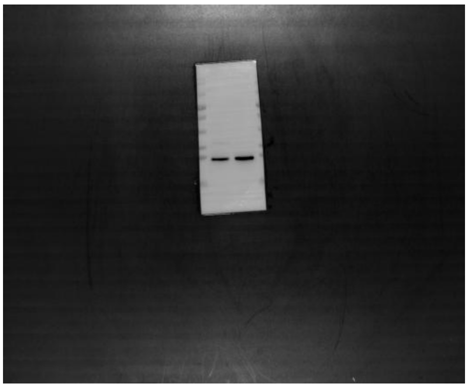

EIF4E

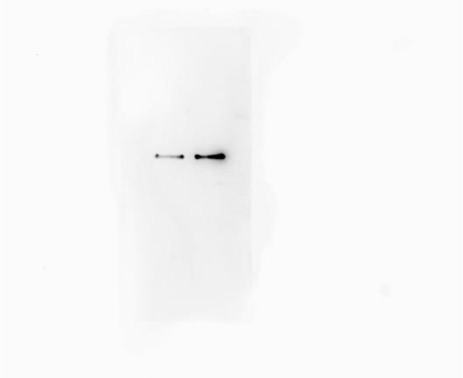

KRT14

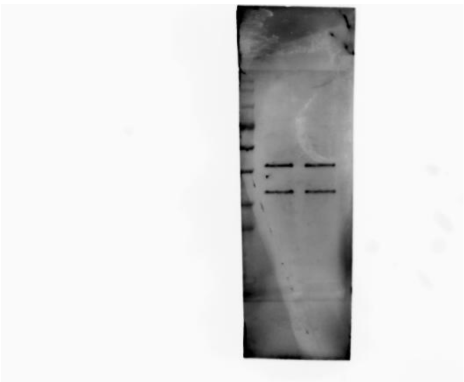

GAPDH

T24-CR

EIFG4

EIF4E

KRT14

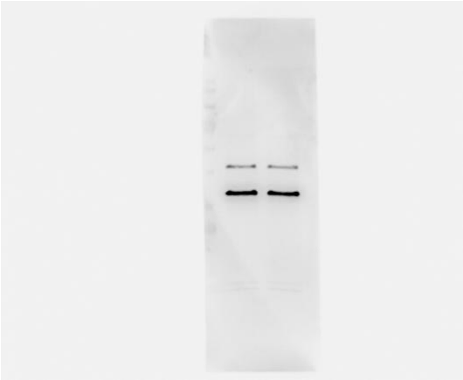

GAPDH

Figure5G

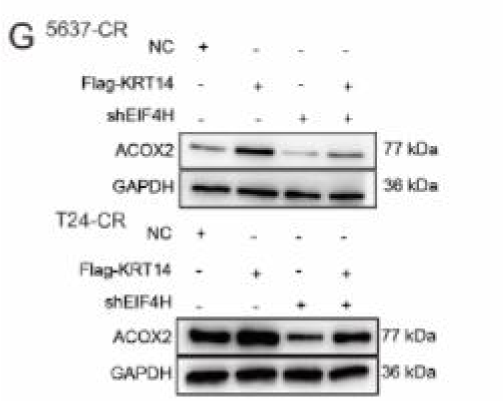

5637-CR

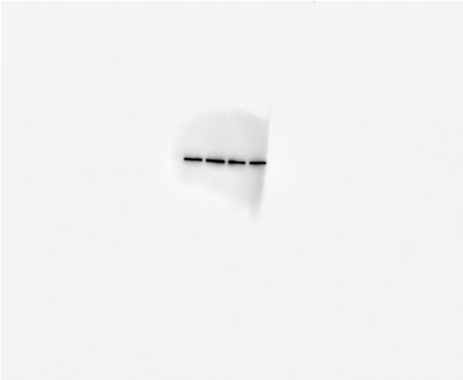

ACOX2 GAPDH

T24-CR

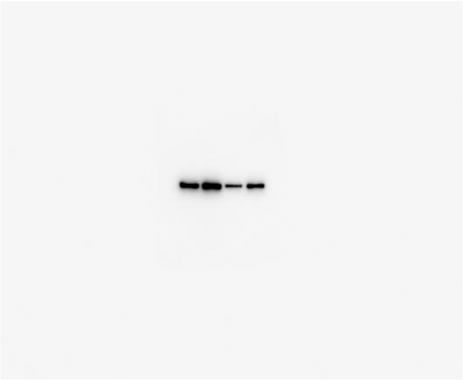

ACOX2 GAPDH

Figure5L

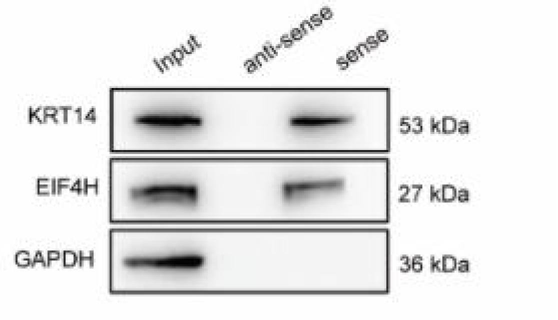

KRT14

ELF4H

GAPDH

Figure 6B

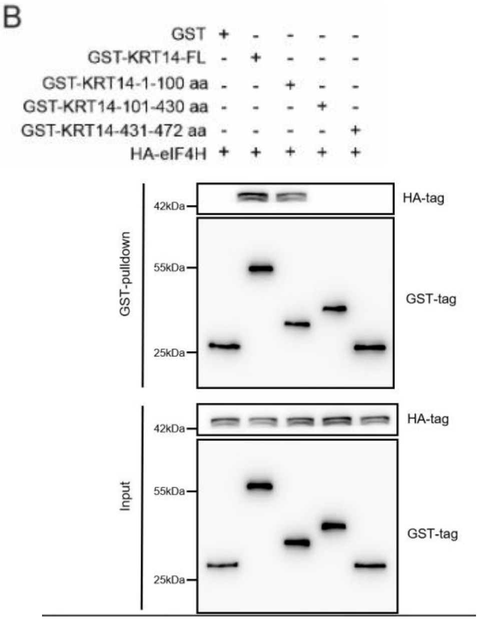

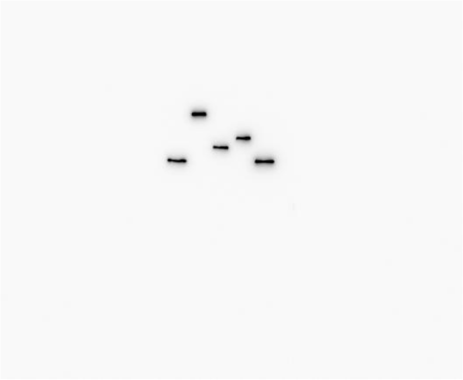

GST-tag

HA-tag

Input

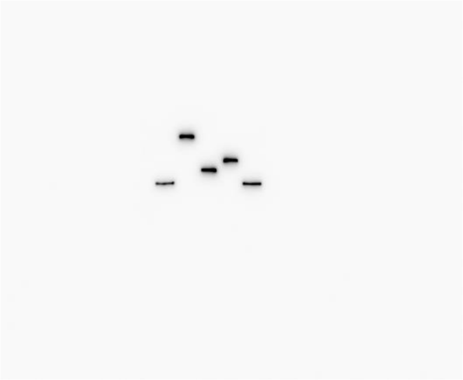

GST-tag

HA-tag

Figure 6C

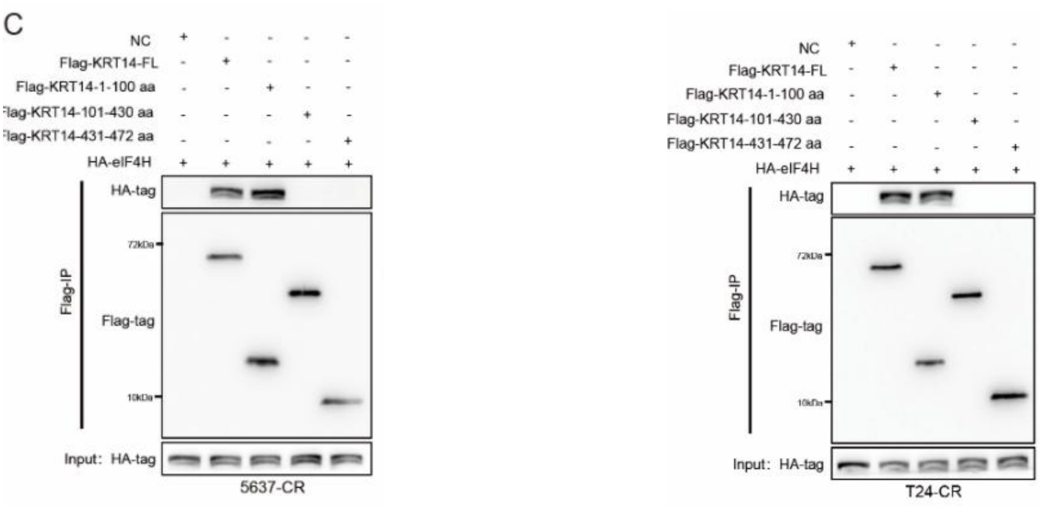

5637-CR

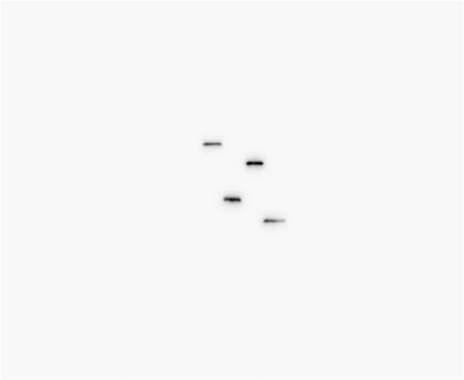

Flag-tag

HA-tag

HA-tag

T24-CR

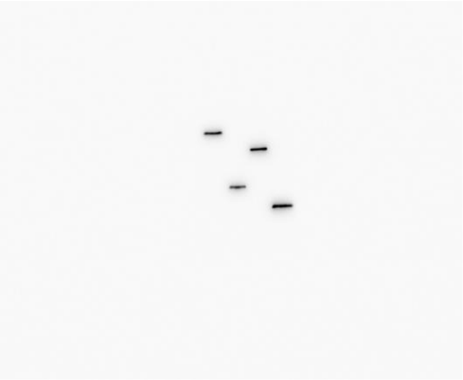

Flag-tag

HA-tag

HA-tag

Figure 6D

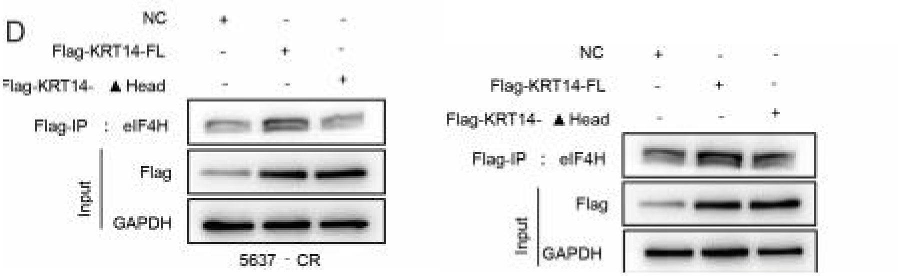

5637-CR

EIF4H

Flag

GAPDH

T24-CR

EIF4H

Flag

GAPDH

Figure 6E

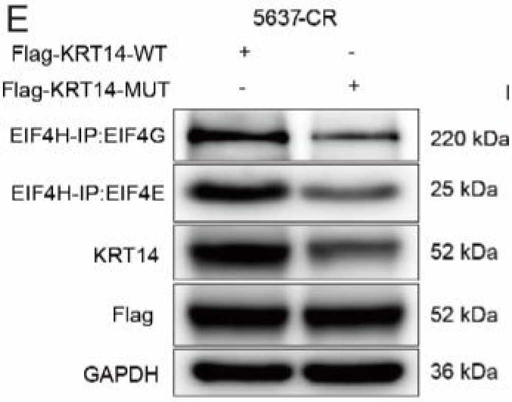

5637-CR

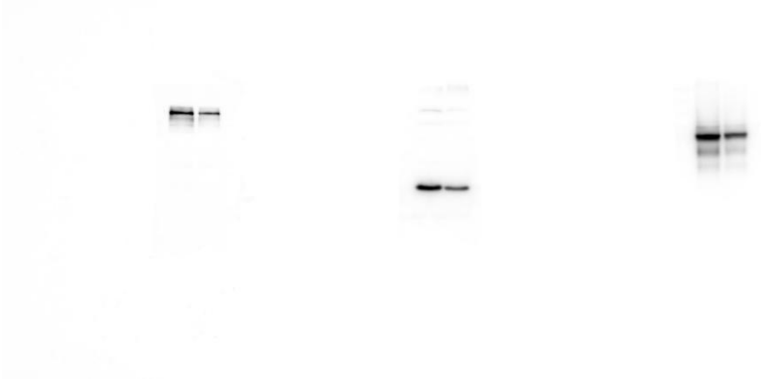

EIF4G ELF4E KRT14

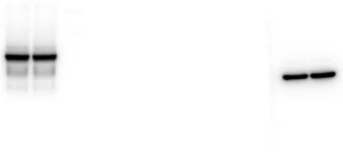

Flag GAPDH

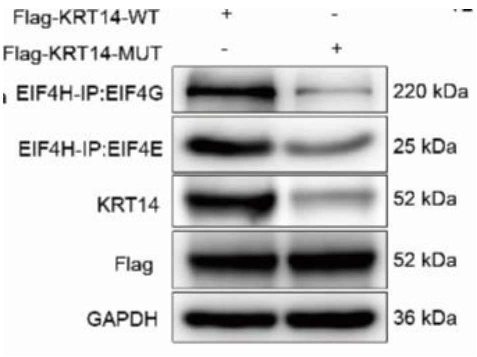

T24-CR

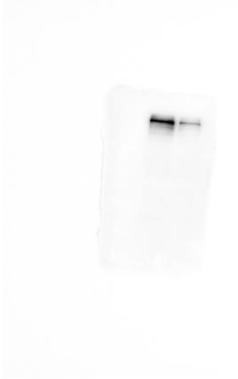

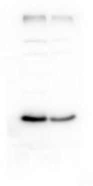

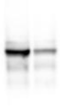

EIF4G ELF4E KRT14

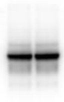

Flag GAPDH

Figure 7E

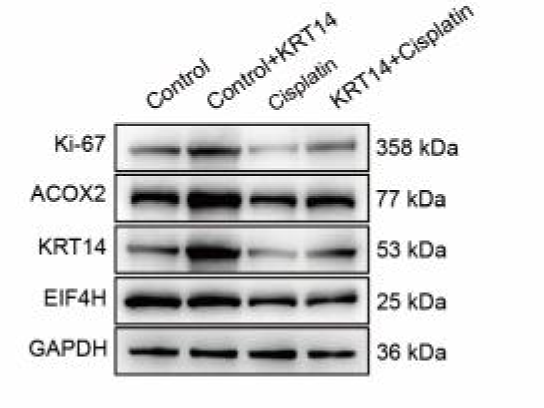

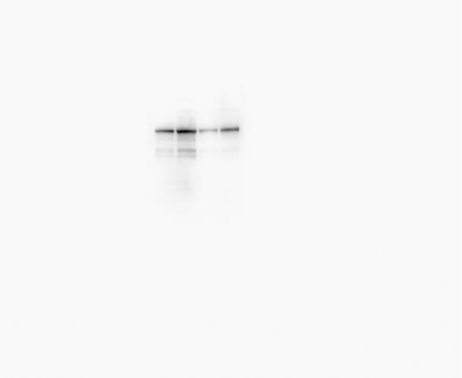

Ki-67

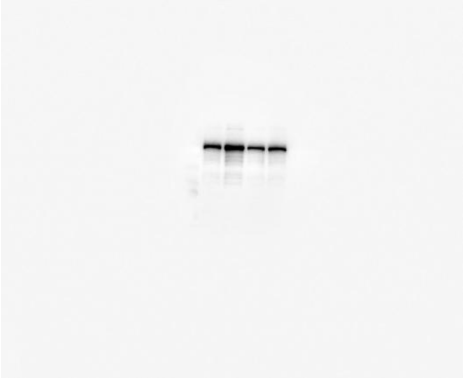

ACOX2

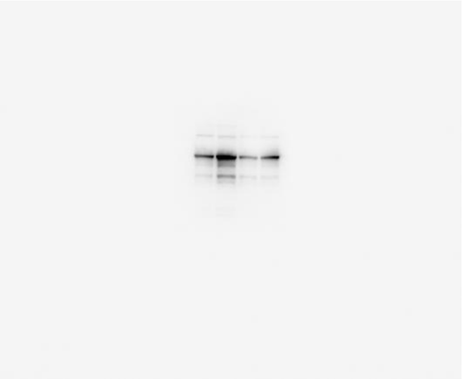

KRT14

EIF4H

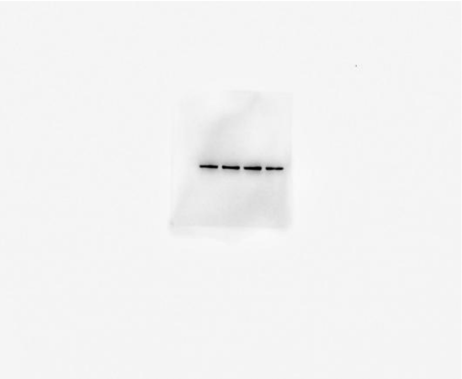

GAPDH

Figure S1C

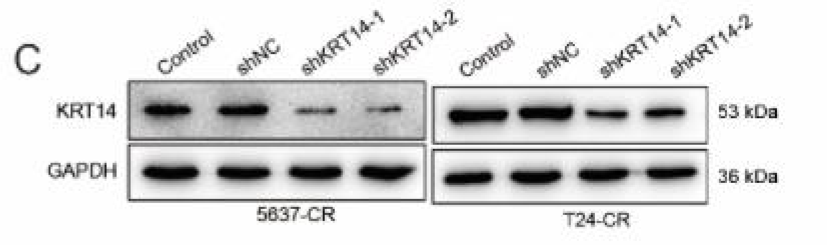

5637-CR

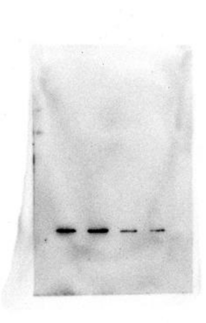

KRT14 GAPDH

T24-CR

KRT14 GAPDH

Figure S1E

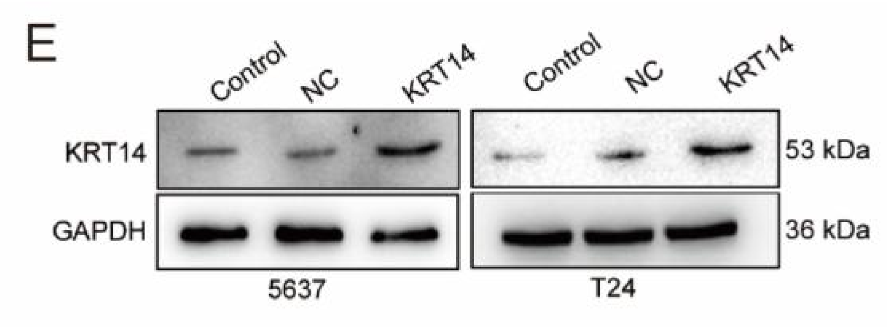

5637

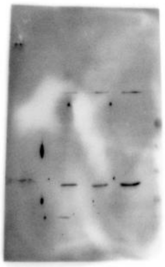

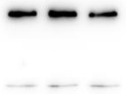

KRT14 GAPDH

T24

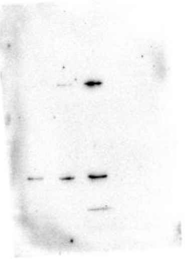

KRT14 GAPDH

Figure S4D

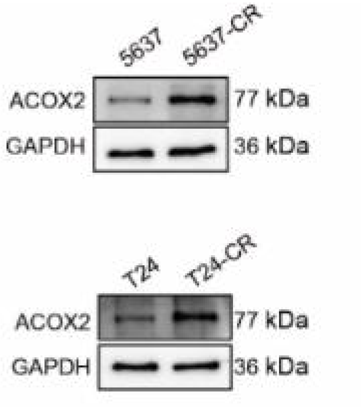

5637-CR

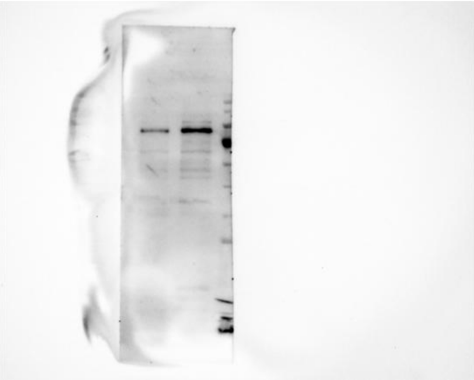

ACOX2

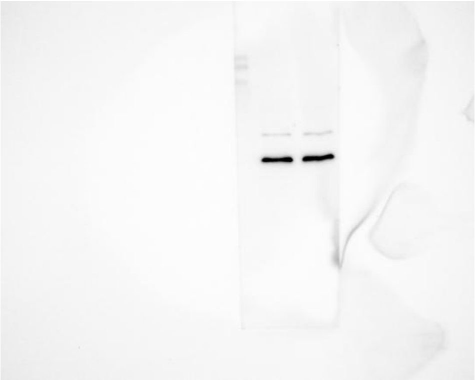

GAPDH

T24-CR

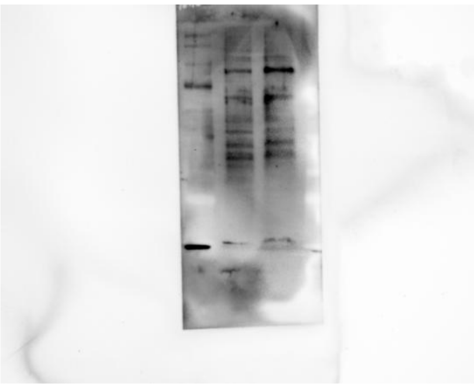

ACOX2

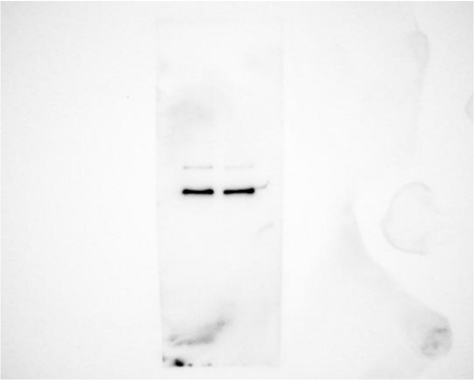

GAPDH

Figure S4F

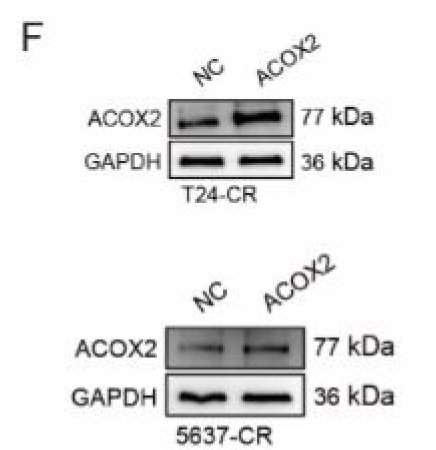

T24-CR

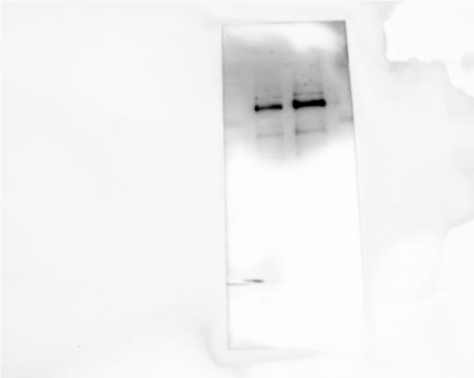

ACOX2 GAPDH

5637-CR

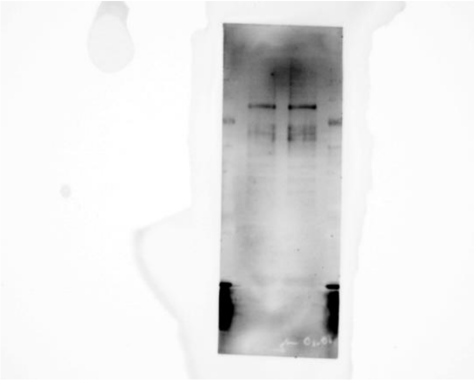

ACOX2 GAPDH

Figure S5C

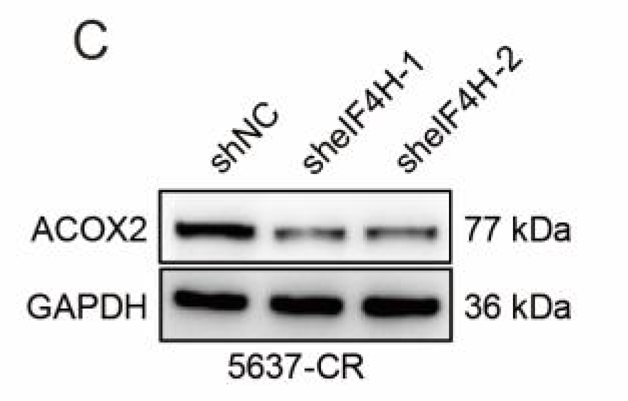

5637-CR

ACOX2

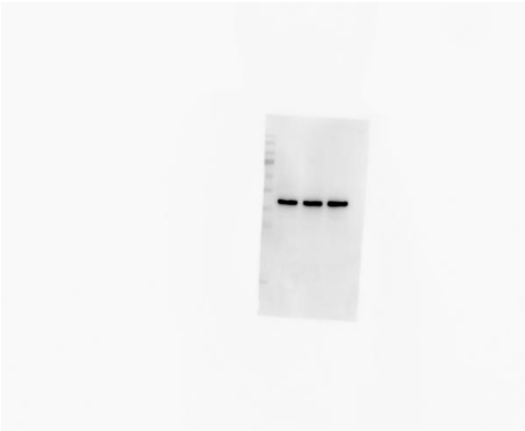

GAPDH

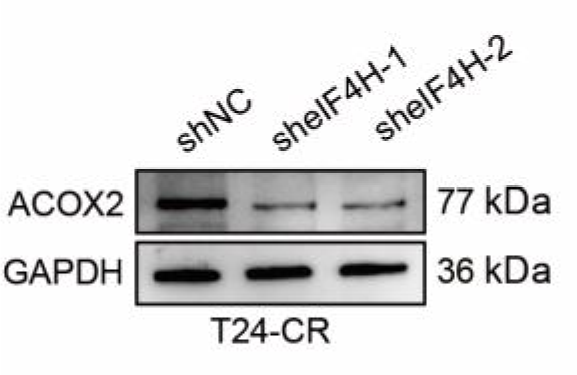

T24-CR

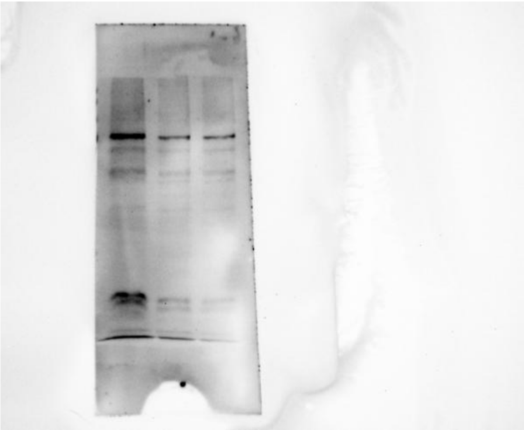

ACOX2

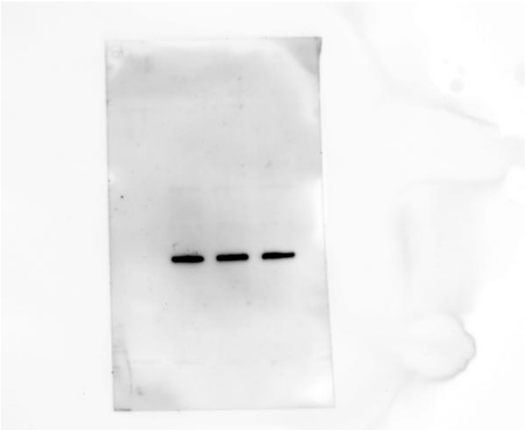

GAPDH

Figure S5D

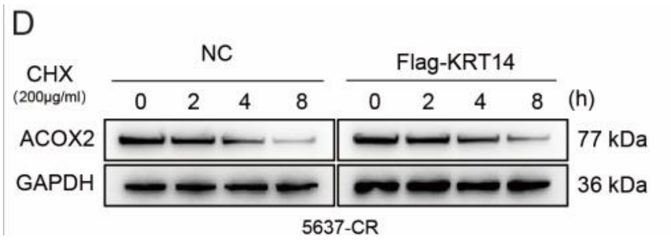

NC

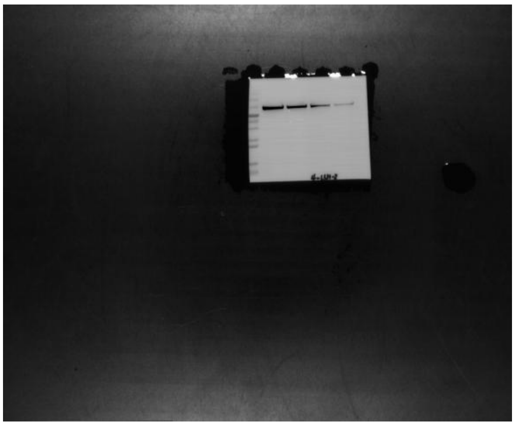

ACOX2

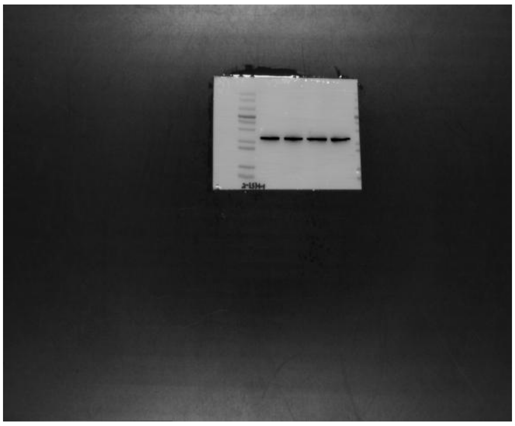

GAPDH

Flag-KRT14

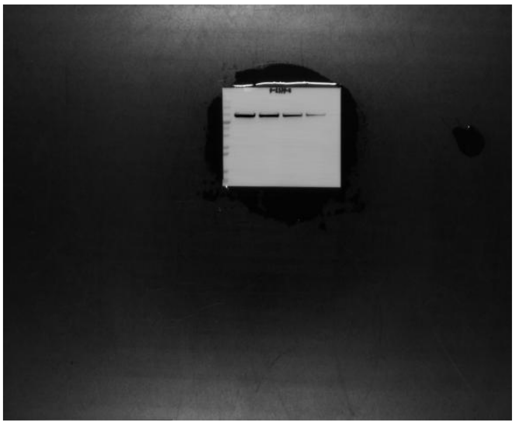

ACOX2

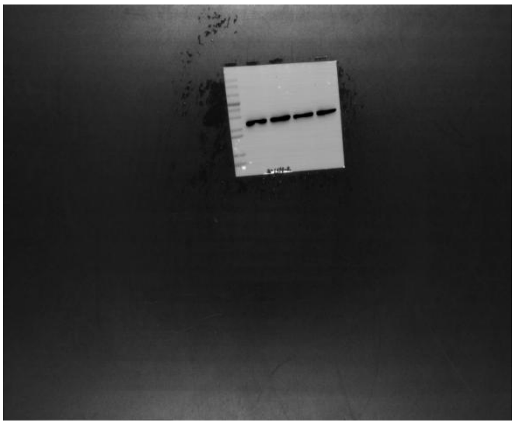

GAPDH

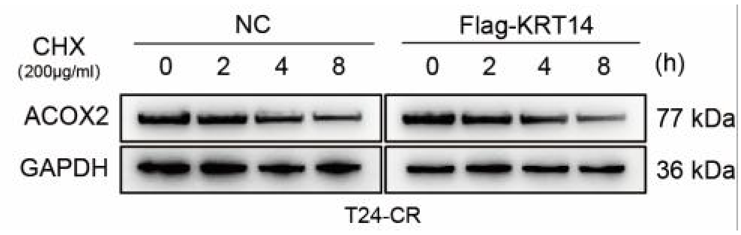

NC

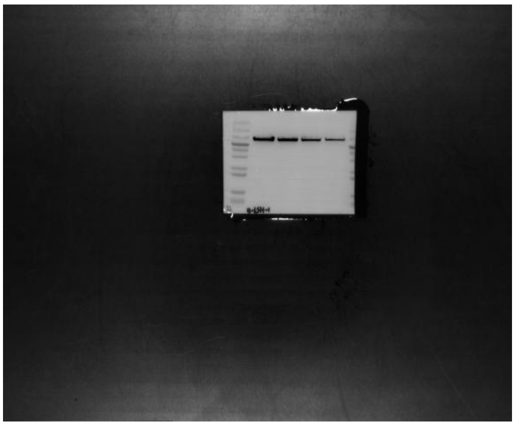

ACOX2

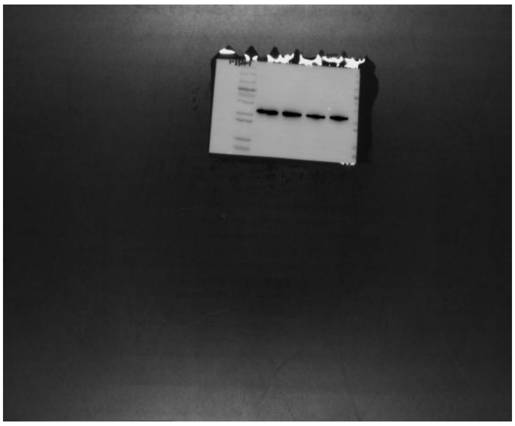

GAPDH

Flag-KRT14

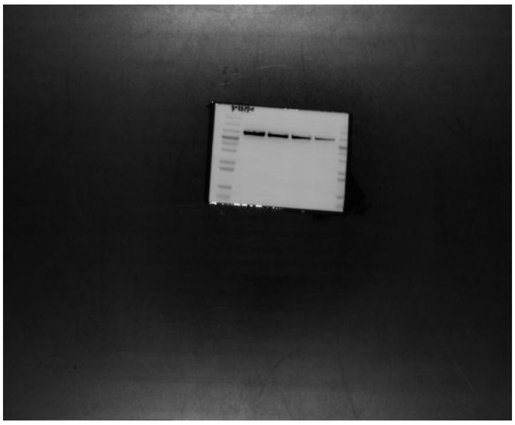

ACOX2

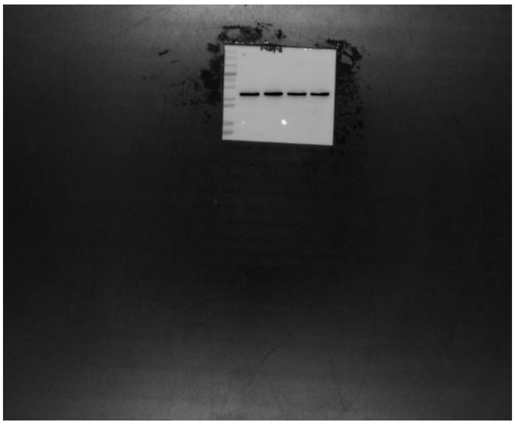

GAPDH

Figure S8A

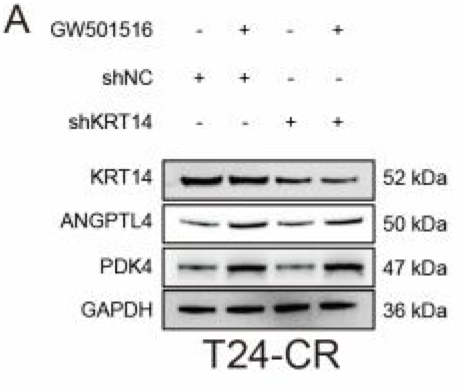

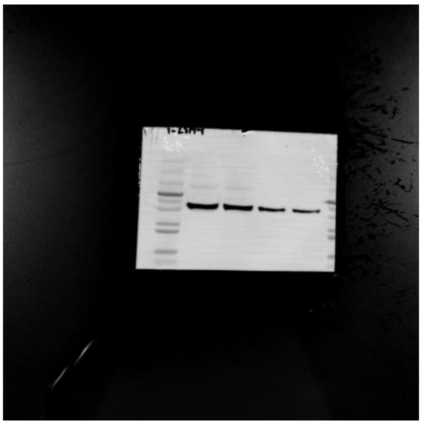

KRT14

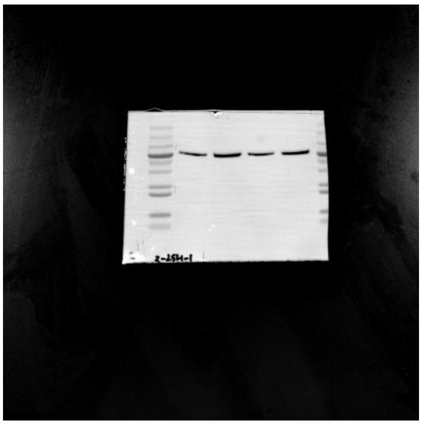

ANGPTL4

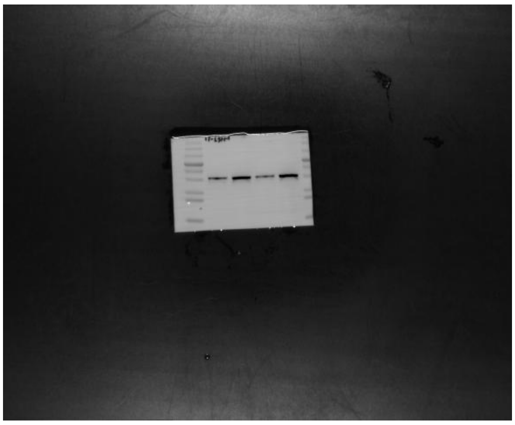

PDK4

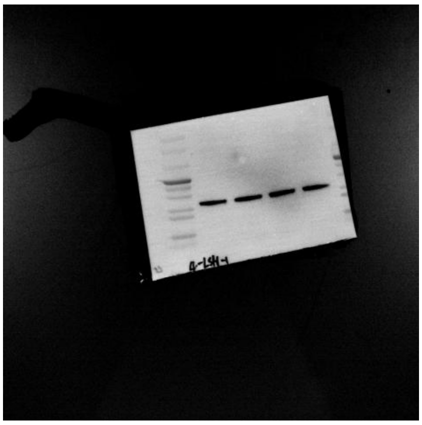

GAPDH

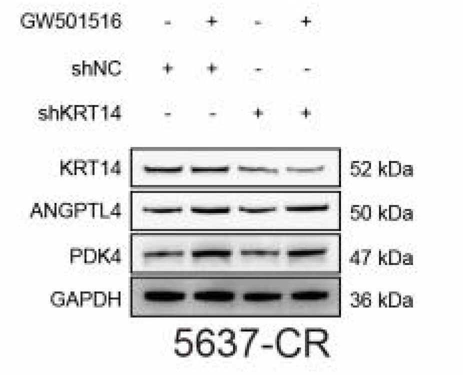

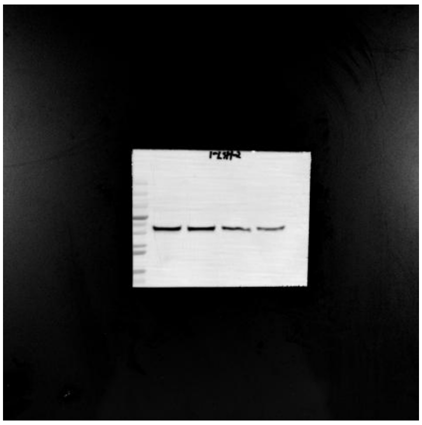

KRT14

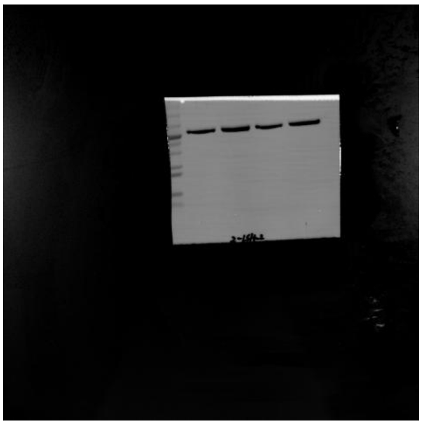

ANGPTL4

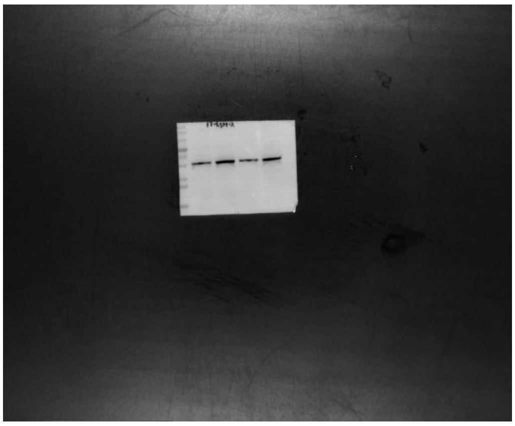

PDK4

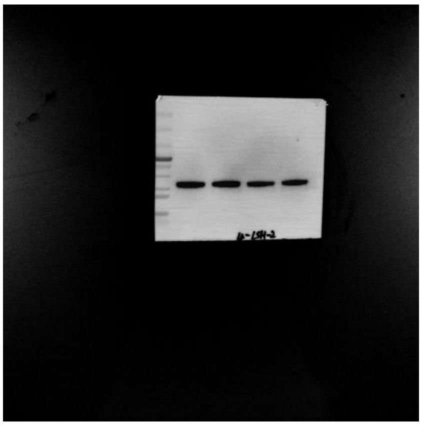

GAPDH

Figure S8C

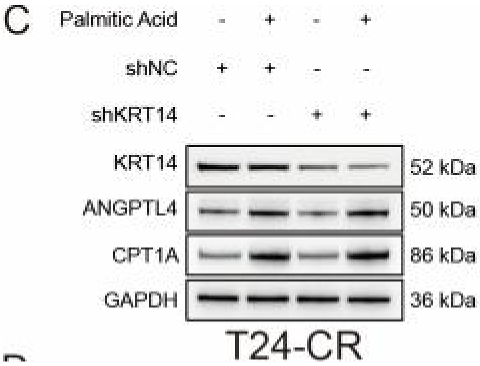

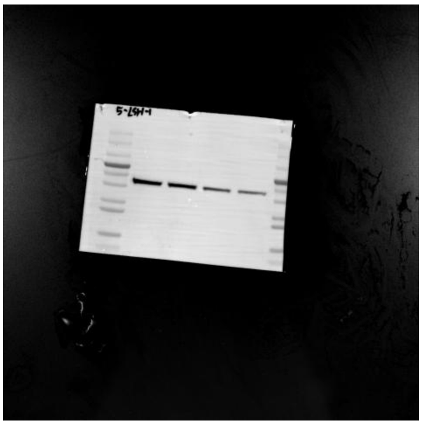

KRT14

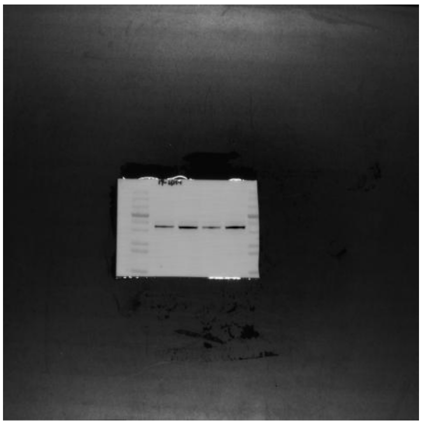

ANGPTL4

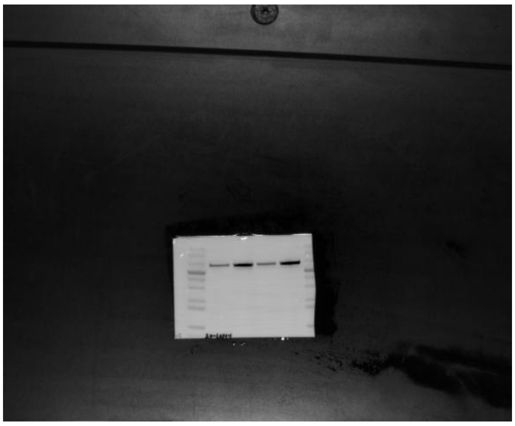

CPT1A

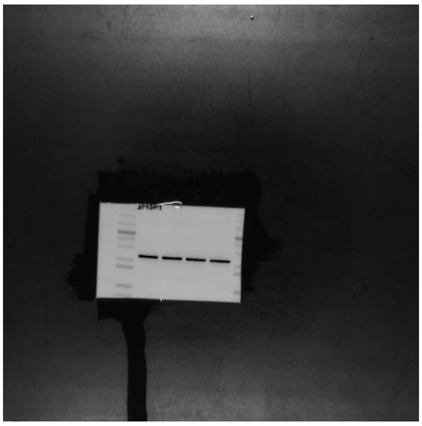

GAPDH

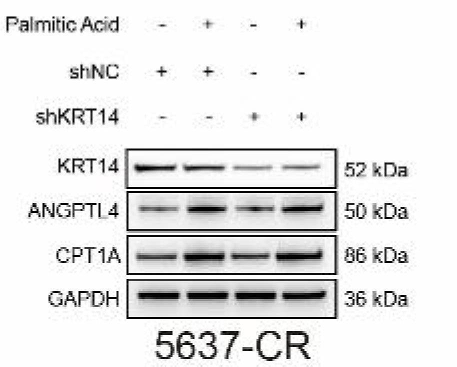

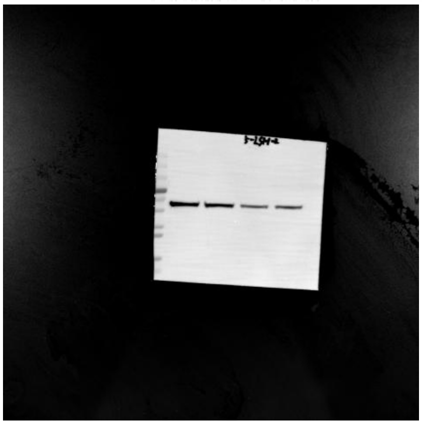

KRT14

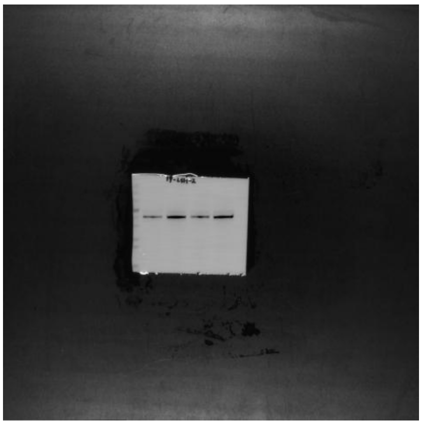

ANGPTL4

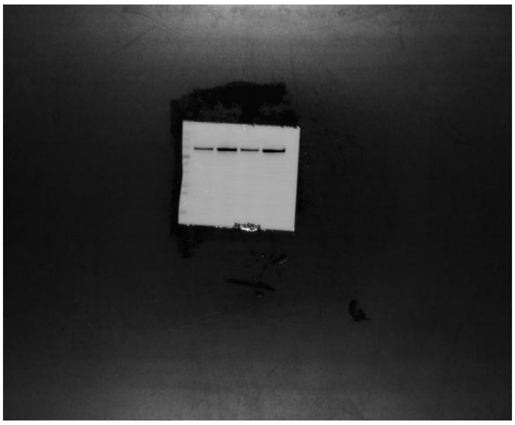

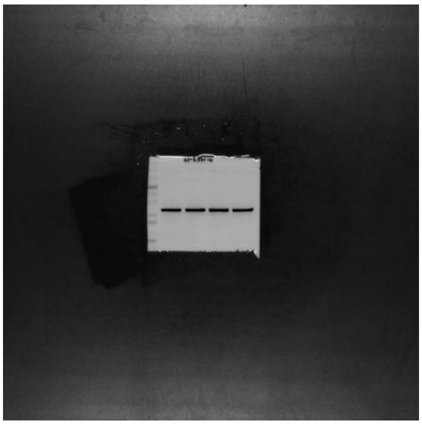

CPT1A GAPDH

Figure S9A

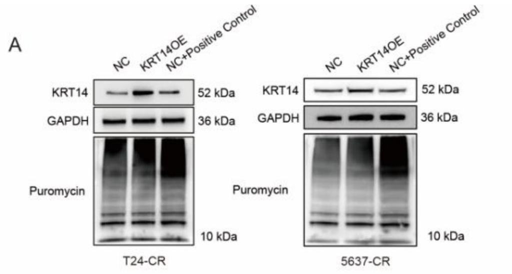

T24-CR

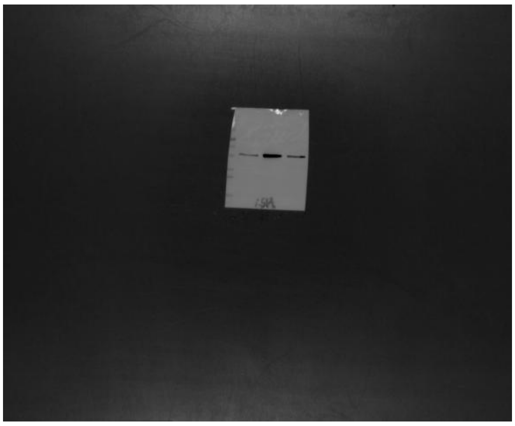

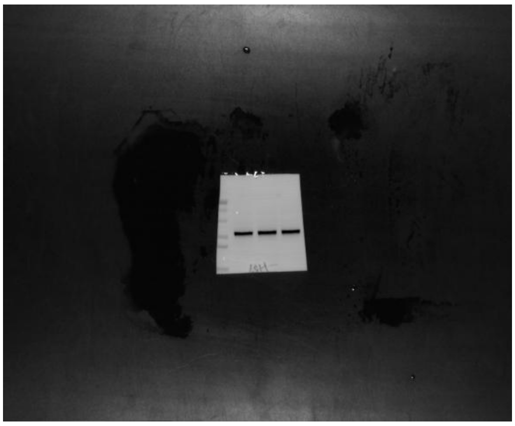

KRT14 GAPDH

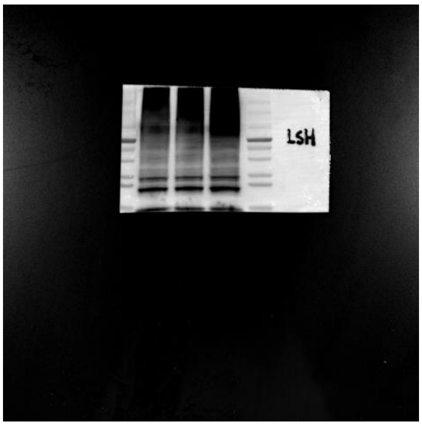

Puro

5637-CR

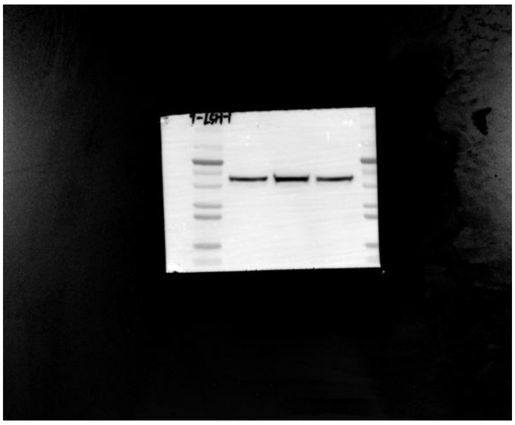

KRT14

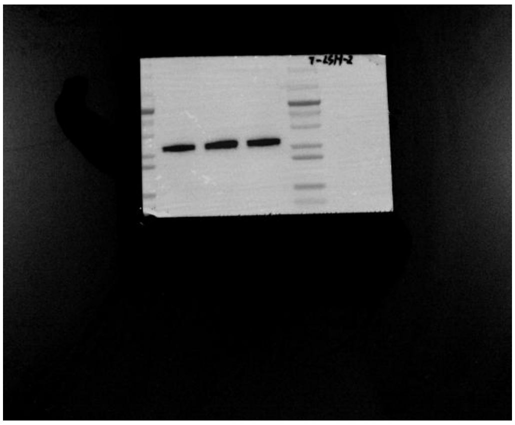

GAPDH

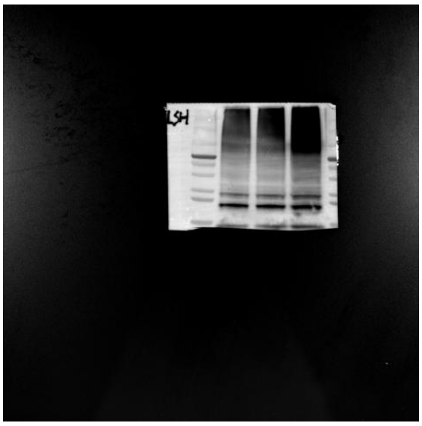

Puro
