# Supplementary material for: Cytoskeletal protein KRT14 governs cisplatin resistance by modulating eIF4H-dependent ACOX2 translation and lipid metabolism in bladder cancer
Source: Cell Death Dis. 2025 Dec 24;17(1):134. doi: 10.1038/s41419-025-08369-3 (PMC12847778; doi:10.1038/s41419-025-08369-3)
Supplement: Supplementary file 12 — Supplementary Table S1 [file 41419_2025_8369_MOESM12_ESM.docx]

Supplementary Table S1

Table 1. Detailed information of the primer sequences for qRT-PCR

| Gene | Sequence |
| --- | --- |
| Human-KRT14_ F | CTACTTCAAGACCATTGAG |
| Human-KRT14_ R | CAACTCTGTCTCATACTTG |
| Human-ACOX2_ F | GGAGACGTGGCCTTAAATAT |
| Human-ACOX2_ R | CCCTGAAGATATGTCCCATG |
| Human-eIF4H_ F | ACATGGATTTCAGAGAACCC |
| Human-eIF4H_ R | GGGATTGGCTACTTGATTGA |
| Human-GAPDH_ F | TCCATGACAACTTTGGTATC |
| Human-GAPDH_ R | CAGGGATGATGTTCTGGA |
| Human-CYP2U1_F | TTGATCTTACCCAACCTGTG |
| Human-CYP2U1_R | GTCCTTGGTCATCCAGAAAT |
| Human-DGAT2_F | ACAGAAGTGAGCAAGAAGTT |
| Human-DGAT2_R | CACGATGATGATAGCATTGC |
| Human-MBOAT7_F | GGCTTCCTTGGAGTATGAC |
| Human-MBOAT7_R | GGTGCGCTCTTGTAGATATA |
| Human-STS_F | CTTGGGCTTCCTTCATTACT |
| Human-STS_R | GGGTGAGATTGTCATAGGAC |
